# The cranial, mandibular, and hyoid anatomy of softshell turtles (*Trionychidae*): A revised character list for phylogenetic analysis

**DOI:** 10.1002/ar.70101

**Published:** 2025-12-06

**Authors:** Léa C. Girard, Walter G. Joyce

**Affiliations:** ^1^ Department of Geosciences University of Fribourg Fribourg Switzerland

**Keywords:** character performance, intraspecific variation, *Testudines*, Trionychia, *Trionychidae*

## Abstract

Softshell turtles (*Pan‐Trionychidae*) are an early branching clade of hidden‐necked turtles (*Cryptodira*) with a rich fossil record extending back to the Early Cretaceous. The evolutionary history of softshell turtles is still unresolved because of their conservative morphology combined with high levels of polymorphism related to morphological plasticity and ontogeny. We present a revised list of characters focused on the skull and hyoid of extant trionychids. To better assess the polymorphism present in extant softshell turtles, we assembled a set of photographs and μCT scans that document the morphology of 312 specimens, individually scored to better document all polymorphism. Several matrices were then assembled that variously reduce observed polymorphism. To explore which matrix performs best under which setting, we carried out numerous parsimony analyses and established phylogenetic accuracy by comparing results to the emerging molecular consensus. The matrix excluding low‐frequency polymorphism and ontogenetically related polymorphism is shown to utilize the greatest amount of character information and performs best with ordered characters and moderate implied weighting, but many relationships remain incorrectly resolved. Use of early fossil trionychids or *Carettochelys insculpta* as outgroups affects the polarity of the trees but leaves the topology largely unchanged. Although our revised list of characters is an improvement upon previous ones, we strongly advise the use of a molecular backbone or total evidence analysis, ideally within a Bayesian framework. A succinct description is provided for the skull and hyoid of all extant clades and species of softshell turtles.

## 1 INTRODUCTION

Softshell turtles (*Trionychidae*) are an early branching group of cryptodires and one of the primary crown groups of extant turtles (*Testudines*). Trionychids are easily recognized externally by their particular morphology consisting of a flattened shell, which lacks keratinous scutes and is covered with thickened skin, and a head adorned by a proboscis and fleshy lips that cover the keratinous rhamphotheca (Ernst & Barbour, 1989; Geoffroy Saint‐Hilaire, 1809; Pritchard, 1979). These characteristics allow softshell turtles to be highly aquatic freshwater ambush predators that bury themselves into the mud to catch prey (Loveridge & Williams, 1957). The skeletal characteristics of softshell turtles include a uniquely textured shell, the loss of peripherals, pygals, and suprapygals, a simplified plastron with loss of the sutural contact between most elements (Pritchard, 1979), and the visible dissociation of the endoskeleton from the exoskeleton resulting in the formation of callosities (Joyce, 2025). The skull morphology of softshell turtles is distinct from other turtles, consisting of an elongated skull, often paired with a long snout, and an open temporal fossa (Vitek & Joyce, 2015). The 35 extant species of *Trionychidae* are today distributed across Africa, North America, Asia, and Papua (TTWG, 2025), but fossils document the former presence of the clade in Europe, Australia, and South America as well (Georgalis & Joyce, 2017; Vitek & Joyce, 2015). The earliest representatives of the total group of trionychids (*Pan‐Trionychidae*) are known from the late Early Cretaceous of China (Brinkman et al., 2017; Li, Joyce, & Liu, 2015; Li, Tong, et al., 2015). These fossils already display all trionychid characteristics listed above, suggesting rapid evolution at the base of the lineage followed by morphological conservativism (Brinkman et al., 2017; Evers et al., 2023; Li, Joyce, & Liu, 2015; Li, Tong, et al., 2015).

Despite an excellent fossil record, the evolutionary history of *Pan‐Trionychidae* remains unresolved, even in the most recent studies, mostly because phylogenetic relationships remain poorly resolved (e.g., Evers et al., 2023; Girard et al., 2024; Jasinski et al., 2022; Ponstein et al., 2024). The phylogenetic position of fossil trionychids is difficult to estimate because of the aforementioned conservative nature of the general morphology of softshell turtles combined with high levels of polymorphism resulting from ontogeny, sexual dimorphism, and phenotypical plasticity (Dalrymple, 1977; Joyce, 2025; Loveridge & Williams, 1957; Meylan, 1987). Meylan (1987) was the first to build a morphological matrix aimed at resolving trionychid phylogeny. This matrix has formed the basis of every subsequent analysis of the group. In addition to a global morphological analysis, Meylan (1987) conducted a separate analysis for the shell, skull, and postcranial partitions only and concluded that the skull partition yields the best results. However, while an informal comparison of Meylan's (1987) skull tree with more recent molecular trees (e.g., Thomson et al., 2021) is overall favorable, a number of clades are incorrectly resolved, thus calling for an expansion of his original character list to help further resolve the phylogeny of fossil trionychids known from skull remains.

Joyce et al. (2025) recently expanded upon the work of Meylan (1987) with a focus on the shell. In an attempt at best practices, he separately scored 530 individuals representing all extant lineages for a revised list of 69 shell characters. He then reduced the observed polymorphism by omitting morphotypes observed in juveniles and by discarding character states with a frequency of less than 20%. In a final step, he investigated which parsimony setting yields results closest to the emerging molecular consensus. Although the revised matrix is not able to retrieve the molecular tree in all details, it performs better than previous matrices thus providing an informed basis for resulting the fossil record of the group.

Herein we apply the protocols developed by Joyce et al. (2025) to the skull anatomy of trionychids, including the hyoid system. We re‐examine characters previously used in trionychid matrices and search for new characters to build a revised matrix that both tackles the polymorphism found in trionychids and helps to better determine the position of fossil trionychids within this turtle lineage. In contrast to Joyce et al. (2025), we heavily rely on the use of micro‐CT scans, a non‐destructive imaging technique that allows access to the internal anatomy of a specimen.

**FIGURE 1:**
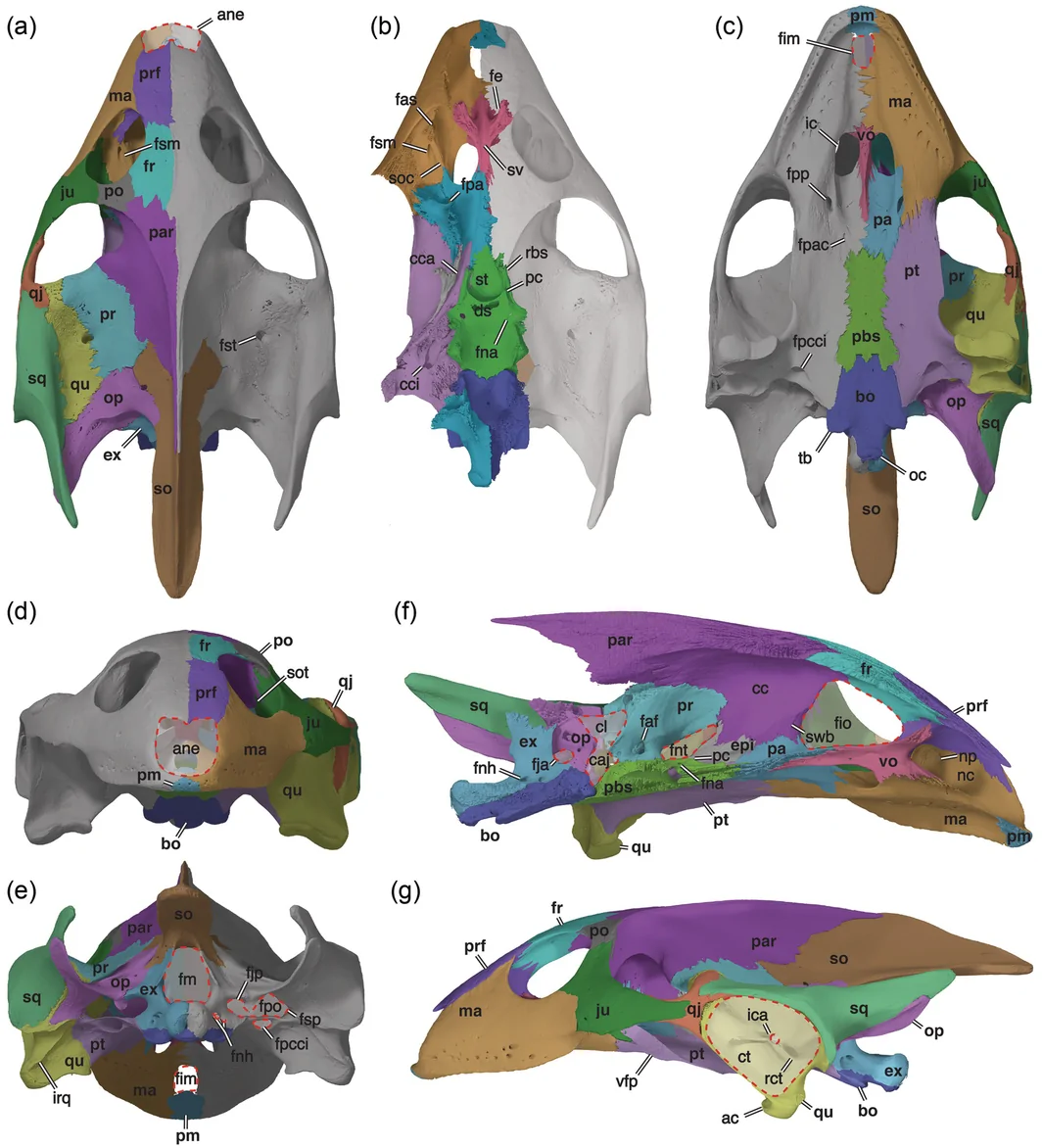
3D model of the cranium *Amyda cartilaginea* FMNH 224117 illustrating anatomical terms used herein. Images are not to scale. (a) dorsal view, (b) internal anatomy in dorsal view, (c) ventral view, (d) anterior view, (e) posterior view, (f) internal anatomy in right lateral view, (g) lateral left view. *ac* articular condyle, *an*e apertura narium externa, *bo* basioccipital, *caj* cavum acustico jugulare, *cc* cavum cranii, *cca* canalis cavernosus, *cci* canalis carotici interni, *cl* cavum labyrinthicum, *ct* cavum tympani, *ds*, dorsum sellae, *epi* epipterygoid, *ex* exoccipital, *faf* fossa accustico‐facialis, *fas* foramen alveolare superius, *fe* fissura ethmoidalis, *fim* intermaxillary foramen, *fio* foramen interorbitale, *fja* foramen jugulare anterius, *fjp* foramen jugulare posterius, *fm* foramen magnum, *fna* foramen nervi abducensis, *fnh* foramina nervi hypoglossi, *fnt* foramen nervi trigemini, *fpa* formen palatinus anterius, *fpac* formen palatinus accessorium, *fpcci* foramen posterius canalis carotici interni, *fpo* fenestra postotica, *fpp* foramen palatinus posterius, *fr* frontal, *fsm* foramen supramaxillare, *fsp* foramen stapedialis posterius, *fst* foramen stapedio‐temporale, *ic* internal choanae, *ica* incisura columellae auris, *irq* infolding ridge of the quadrate, *ju* jugal, *mx* maxilla, *nc* nasal cavity, *np* nasal pocket, *oc* occipital condyle, *op* opisthotic, *pa* palatine, *par* parietal, *pb* parabasisphenoid, *pc* processus clinoideus, *pf* prefrontal, *pm* premaxilla, *po* postorbital, *pr* prootic, *pra* prearticular, *pt* pterygoid, *qj* quadratojugal, *qu* quadrate, *rbs* rostrum basisphenoidale, *rct* ridge of the cavum tympani, *so* supraoccipital, *soc* suborbital crest, *sot* septum orbito‐temporale, *sur* surangular, *sq* squamosal, *st* sella turcica, *sv* sulcus vomeri, *swb* secondary wall of the braincase, *tb* tuberculum basioccipitalis, *v* vomer, *ptf* pterygoid flange.

*Anatomical nomenclature*: Unless indicated otherwise, we herein use the standard anatomical nomenclature of Gaffney (1972) for turtles in general and of Meylan (1987) for trionychids in particular, with additions from Evers et al. (2022, 2023) and Girard et al. (2024). Nomenclature for the neuroanatomy follows Soliman (1964), Albrecht (1967), and Rollot et al. (2021). To guide readers less familiar with trionychid anatomy, all bones and the main anatomical features of the cranium are labeled in Figure 1. Additional structures are labeled in the relevant figures.

*Institutional abbreviations*: AMNH, American Museum of Natural History, New York, USA; CUMZ, Chulalongkorn University Museum of Natural History, Bangkok, Thailand; FMNH, Field Museum of Natural History, Chicago, Illinois, USA; IBH, Institute of Biotechnology, Hanoi, Vietnam; IEBR, Institute of Ecology and Biological Resources, Hanoi, Vietnam; LSUMZ, Louisiana Museum of Natural History, Baton Rouge, Louisiana, USA; MCZ, Museum of Comparative Zoology, Cambridge, Massachusetts, USA; MNHN, Muséum national d'Histoire naturelle, Paris, France; MTD, Museum für Tierkunde Dresden, Dresden, Germany; NHMB, Naturhistorisches Museum Basel, Basel, Switzerland; NHMUK, Natural History Museum, London, UK; NMB, Museum für Naturkunde Berlin, Berlin, Germany; NMW, Naturhistorisches Museum Wien, Vienna, Austria; NSM, National Museum of Science and Nature, Tokyo, Japan; MVZ, Museum of Vertebrate Zoology, Berkeley, California, USA; PCHP, Peter C. H. Pritchard Collection at the Turtle Conservancy, Ojai, California, USA; RH, Ren Hirayama Collection at Waseda University, Tokyo, Japan; ROM, Royal Ontario Museum, Toronto, Canada; SMF, Senckenberg Naturmuseum, Frankfurt, Germany; TMM, Texas Memorial Museum, Austin, Texas, USA; UCMP, University of California Museum of Paleontology, Berkeley, California, USA; UCMVZ, Museum of Vertebrate Zoology, Berkeley, California, USA; UF, University of Florida, Gainesville, Florida, USA; UMMZ, Museum of Zoology, Ann Arbor, Michigan, USA; USNM, National Museum of Natural History, Washington DC, USA; YPM, Yale Peabody Museum of Natural History, New Haven, Connecticut, USA; ZSI, Zoological Survey of India, Kolkata, India.

## 2 MATERIALS AND METHODS

### 2.1 Sampling

*Trionychidae* is a clade of turtles with known high levels of intraspecific variation associated with growth, sexual dimorphism, and phenotypic plasticity (Dalrymple, 1977; Joyce, 2025; Meylan, 1987). To better document the anatomy of these turtles, it is therefore essential to understand inter‐ and intraspecific variation through the study of a large sample. While many natural history collections across the globe house softshell turtle specimens, taxonomic diversity and specimen numbers are universally insufficient to adequately study variation. As it was unfeasible within the confines of this study to visit collections to gather sufficient data through direct observation, we mainly scored and measured specimens using photographs supplemented by CT scans. This allowed for the repeated assessment of specimens and for making adjustments to character definitions and specimen scorings. However, the use of photographs introduces several limitations. First, the angles, lighting, and focus of the photographs available to us did not always allow for the complete scoring of all specimens as these pictures were taken by different people over the course of several years, often using suboptimal equipment and lighting and without standardized procedures. However, as our sample is large, we were able to overcome this shortcoming for most characters, but sampling remains poor for some. These difficulties are indicated in our character descriptions below. Another problem arising from the use of photographs is the distortion of morphometric characters. To minimize this issue, we developed characters that demand photographs taken in standard, orthogonal views and utilize polymorphic scoring near the threshold to address uncertainty (see Section 2.2).

We assembled a set of pictures and models from the following sources. The first source is the set of pictures uploaded by Joyce et al. (2025) to Dryad (doi.org/10.5061/dryad.jq2bvq8m2) consisting of the pictures of 608 taxonomically confirmed trionychid specimens. From this dataset, we only selected specimens with pictures that sufficiently document the skull and/or hyoid. This set of pictures was supplemented by specimens with CT scans available for download at MorphoSource, specimens CT scanned at our request, or specimens scanned by ourselves at the CT‐scanning facilities of the Department of Geosciences at the University of Fribourg. Following Joyce et al. (2025), all specimens in our sample are referred to currently recognized trionychid species (TTWG, 2025), with the exception of all representatives of *Pelodiscus*, which were not identified to the species level because the skeletal anatomy of *Pelodiscus* species has not been described, because locality data is typically unreliable, and because most specimens likely represent hybrid individuals originating from farms (Gong et al., 2022). Our total sample consists of digital photographs and/or CT scans of 312 specimens housed in 27 natural history collections across the globe (see Data S1, Supporting Information).

Among the set of CT scans available to us, we performed bone‐by‐bone segmentation on half the cranium and mandible (when available) of 29 specimens covering all recognized genera of softshell turtles using the software Materialise Mimics v.24. All scans and associated 3D models are either available for download at MorphoSource (https://www.morphosource.org/projects/000739536) or the Data Depository of NHMW (https://doi.org/10.57756/gtse5e).

### 2.2 Characters

Meylan (1987) developed a set of 68 characters to investigate trionychid relationships, of which 38 characters pertain to the cranium, mandible, and hyoid complex. Of these 38 characters, 9 characters (i.e., 32, 34, 51, 65, 68, 70, 71, 73, 98) utilize scaled coding (i.e., character states defined by the presence of polymorphism), 13 characters are multistate (i.e., 34, 41, 53, 60, 64, 65, 68, 70, 71, 73–75, 94), and 2 characters are morphometric (i.e., 74, 78). This list of skull and hyoid characters was later supplemented by 5 skull characters from Joyce and Lyson (2011), 1 hyoid character from Brinkman et al. (2017), 1 cranial character from Vitek et al. (2018), and 26 skull characters from Evers et al. (2023).

In an attempt to convert as much morphological information as possible into phylogenetic characters, we reviewed all character complexes and bones individually, while taking all previously developed characters into full consideration. As the use of multistate and morphometric characters had proven useful for the shell (Joyce, 2025), we placed efforts into developing characters that can be measured objectively and utilize the full spectrum of measurements through the use of morphoclines. A number of characters were discarded after scoring, either because we did not feel confident about the reproducibility of particular measurements, or because our sample size was too small, or because the available character distribution had questionable phylogenetic relevance. We list some of the more pertinent omitted characters below, especially characters used by previous authors (see Section 3.2). We checked the literature for authorship of all characters we developed herein, but given the vast nature of the literature, we may have inadvertently overlooked previous iterations. Our final matrix consists of 92 characters, including 77 cranial characters, 12 mandibular characters and 3 hyoid characters. Among these characters, 18 are morphometric characters and 35 are multistate characters. The full list of figured characters is provided below.

### 2.3 Scoring of individuals and the coding of terminal taxa

The aim of this work is to provide an updated morphological matrix of the skull and hyoid of softshell turtles that accounts for the particularly high levels of intraspecific variation (i.e., polymorphism) found in this group of turtles (Dalrymple, 1977; Joyce, 2025; Meylan, 1987; Webb, 1962). For this purpose, we follow Joyce et al. (2025) by initially scoring all characters for all individuals separately. We hereby liberally score individuals as polymorphic, if they display left/right asymmetry or if they exhibit intermediate morphologies, in particular morphometric measurements near a given threshold, typically 2 percentage points (see Section 3.1). Morphometric measurements were obtained from pictures of specimens or screenshots of 3D models in orthogonal views using ImageJ (Schneider et al., 2012). All morphometric measurements are available in Data S1. The scoring of specimens is provided in Data S2. The scoring of individual specimens has several benefits, including the coding of species after all primary observations have been collected, the simplified addition of further individuals to the matrix, and the exploration of alternative coding schemes.

As many of the characters presented in this study exhibit considerable intraspecific variation, we follow Joyce et al. (2025) and reduce observed polymorphism when coding species by disregarding all character states with a frequency below 20%. In the case that our initial scoring introduced polymorphism to reflect right/left asymmetry, we regard both sides of the skull for the full sample as two separate data points to establish frequencies (i.e., if one out of five skulls shows left/asymmetry for a particular character and the remaining sample scores consistently absent, the frequency is 10%). In the case that our initial scoring introduced polymorphism to address intermediate observations that cross a threshold, we initially reduce polymorphism by assigning specimens spanning a threshold to the majority state and the remaining polymorphism if it occurs below a frequency of 20%. We name the matrix with reduced polymorphism *RedPoly*. To assess the effect of our coding strategy upon phylogeny, we create a second matrix, named *MaxPoly*, that conserves all polymorphism found in the primary data. We only list character distributions for the *RedPoly* matrix in our character descriptions below. Both matrices are available in Data S3 and S4.

Joyce et al. (2025) documented significant polymorphism in the shell of trionychids related to ontogeny that he secondarily reduced in his taxon matrix by removing character states exhibited by juveniles. An informal analysis of our data using the same methods as employed by Joyce et al. (2025) (i.e., exploring for correlations between the relative size of specimens and its scoring) concludes that the relationship of ontogeny seems more complex for the cranium than what had been observed for the shells (see Section 3). In particular, while Joyce et al. (2025) observed that most species exhibit similar positive or negative correlations for a given character, we observed strongly positive to strongly negative correlations for the same character in skulls. This complicates reducing ontogeny‐based polymorphism, as the scoring of each species needs to be adjusted individually, which is not possible, as not all terminal taxa are sufficiently sampled. To better assess the effect of ontogeny on phylogenetic assessment, we build two additional matrices that disregard polymorphism below two select size thresholds as a proxy for ontogeny. Our *Size60* matrix only includes in our scoring of terminal specimens with a cranium length of at least 60% of the maximum known cranium length for a terminal, and our *Size70* matrix only includes specimens that are at least 70% of the maximum cranium length. In both cases, polymorphism is further reduced using the protocols described in the previous paragraph. As some characters obviously correlate with ontogeny, this approach automatically favors the morphotype of adult specimens, regardless of whether the correlation with ontogeny is positive or negative. The *Size60* and *Size70* matrices are available in Data S5 and S6, respectively. The absolute and relative size of each specimen are reported in Data S1. It should be noted that some trionychids, in particular *Apalone* spp., exhibit pronounced sexual size dimorphism (Dalrymple, 1977; Meylan, 1987; Webb, 1962), so this coding strategy categorically removes the morphology of males from the sample. We are not able to adjust for this bias, however, as most individuals in our sample lack sex data.

### 2.4 Phylogenetic analyses

We conducted all of our parsimony analyses using TNT 1.5 (Goloboff & Catalano, 2016). The number of maximum trees in memory was set to 99,999. The analyses were performed using a traditional search with 100 random seeds and 1000 replications followed by a round of tree bisection reconnection (TBR). Several analyses were performed that differ in the use of unordered versus ordered characters (i.e. Characters 4, 6–11, 13, 17, 21, 28, 31, 34–37, 44–46, 49–52, 55, 56, 63, 69, 72, 74, 75, 77, 79, 82, 91, using regular counter starting at 1) and the use of equal and implied weights (K = 3 and 6; Ezcurra, 2024; Goloboff et al., 2018). To assess which parsimony settings yield the most accurate results, we follow Joyce et al. (2025) by comparing our results to a molecular consensus reference tree built from Engstrom et al. (2004), Le et al. (2014), and Thomson et al. (2021) that includes the same terminal taxa as used in our analyses (also see Joyce, 2025). The trees were compared in RStudio version 4.4.2 (Posit Team, 2024) using the Quartet package version 1.2.7 (Smith, 2019). This package allows us to measure the symmetric difference between quartets (four‐taxon statements) between two trees. As many analyses yielded more than a single most parsimonious tree (MPT), we ran separate quartet analyses for each MPT and report averages for all MPTs in Table 1. Individual results of all MPTs are provided in Data S7.

## 3 RESULTS AND DISCUSSION

### 3.1 List of characters

Character 1: Premaxilla 1

**FIGURE 2:**
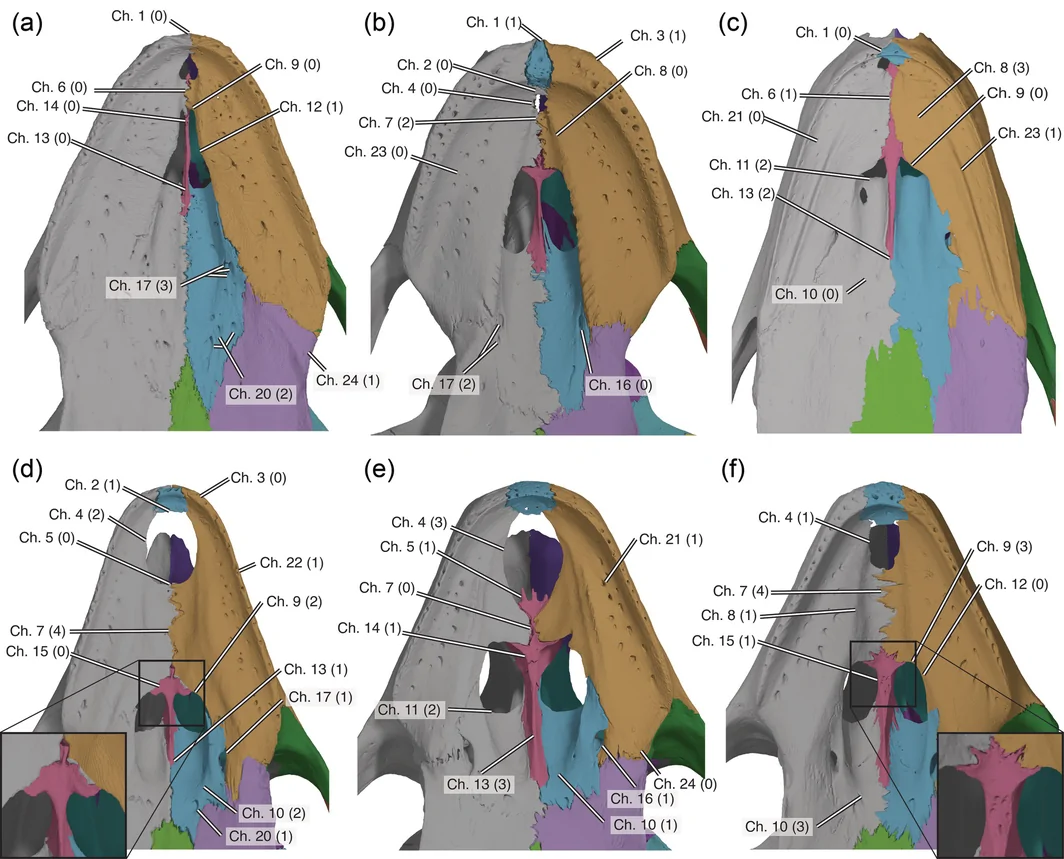
3D models of crania of select trionychid species in ventral view, focusing on the palatal area, illustrating character states. (a) *Cycloderma frenatum* NHMUK 84.2.4.1, (b) *Cyclanorbis elegans* NHMW 157, (c) *Chitra chitra* PCHP 4995, (d) *Trionyx triunguis* PCHP 4559, (e) *Rafetus euphraticus* NHMW 132, (f) *Amyda cartilaginea* FMNH 224117. Images are not to scale.

*Definition*—Premaxilla (modified from Meylan, 1987, 65): 0 = absent (Figure 2a), or very reduced (Figure 2c); 1 = present (Figure 2b).

*Comment*—This character aims to document the genuine absence of a premaxilla, not its post mortem loss, as this bone easily disconnects from the skull and is therefore often missing in extant and fossil specimens. However, while a missing premaxilla leaves an empty notch, an absent premaxilla is compensated for by a complete median contact of the maxillae.

We document the variable absence of a premaxilla in *Pelochelys bibroni*, *Chitra indica*, and *Cycloderma* spp. We find no single taxon that systematically lacks a premaxilla, but a clear majority of specimens attributable to *Cycloderma* spp. lack this bone (Figure 2a), while a premaxilla is highly reduced in the remainders (Figure 2c). We, therefore, expand the absence character state to include the highly reduced condition. As with many other characters, we implement a 20% threshold for establishing the final scoring of species, which means that species are only scored polymorphic if at least one in five specimens exhibits the second character state. This differs from Meylan (1987), who distinguished between taxa that “occasionally” lack a premaxilla, versus those that “usually” lack this bone, cutoffs that we found to be neither practical, nor supported by the available data. Indeed, Meylan (1987) scored *Chitra* spp. (his *Chitra indica*) and *Cycloderma frenatum* as often lacking a premaxilla and *Pelochelys bibroni* as usually lacking one, which varies from our result. We speculate that this difference is based on Meylan's (1987) inclusion of specimens that taphonomically lost their premaxilla as lacking this bone, as we note that many chitrines lose the premaxilla bone postmortem.

Character 2: Premaxilla 2

*Definition*—Contribution of the premaxilla to the anterior margin of the foramen intermaxillare (new character): 0 = absent (Figure 2b); 1 = present (Figure 2d).

*Comment*—The premaxilla contributes to the foramen intermaxillare in the vast majority of trionychids (Figure 2c–f), but this contribution is variably absent in our sample of *Chitra chitra*, *Cyclanorbis senegalensis*, and *Lissemys punctata*, absent in our single specimen of *Lissemys scutata*, absent in the only individual of *Cycloderma aubryi* that exhibits a premaxilla, and consistently absent in our sample of *Cyclanorbis elegans* (Figure 1b). Species and specimens lacking a premaxilla are scored as non‐applicable (Figure 2a). Polymorphism is scored implementing our standard scoring protocol (see Section 2). As the premaxilla is only rarely excluded from the foramen intermaxillare in *Lissemys punctata*, we have low confidence that the scoring of our single specimens of *Lissemys scutata* is representative of this species.

Character 3: Maxilla

*Definition*—Thickening of the labial ridge of the maxilla near its contact with the premaxilla (new character): 0 = absent (Figure 2d); 1 = present (Figure 2b).

*Comment*—As in most turtles, the labial ridge of trionychids has an even thickness, but a noticeable thickening is apparent to the labial ridge of the maxilla just posterior to the contact of the maxilla with the premaxilla in some specimens (Figure 2b). This condition is consistently observed in *Cyclanorbis elegans*. It is additionally present in some specimens of *Amyda* spp. (Figure 2f), but as these specimens constitute less than 20% of the total sample, we do not score *Amyda* spp. as polymorphic.

Character 4: Foramen intermaxillare 1

**FIGURE 3:**
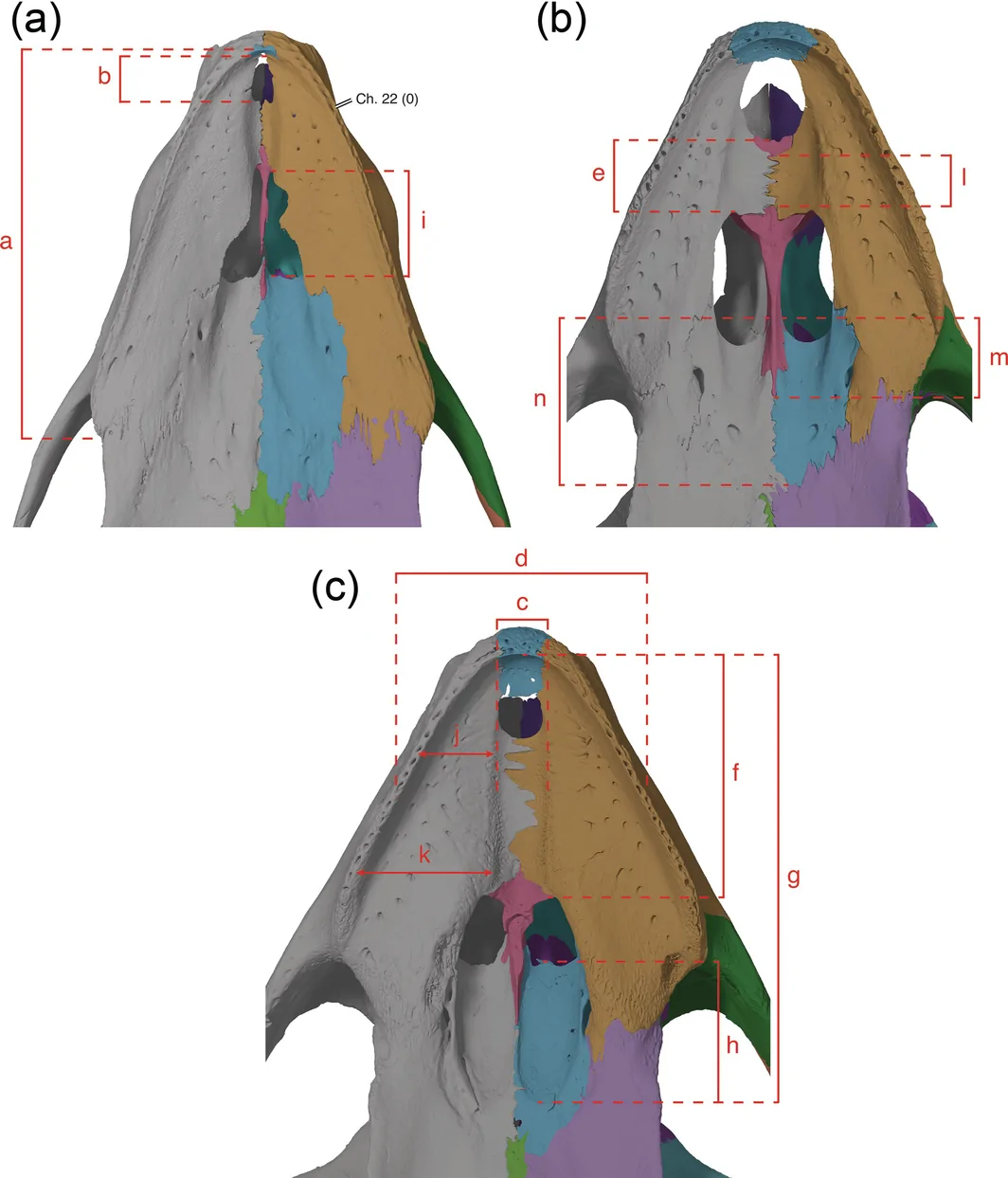
3D models of crania of select trionychid species in ventral view, focusing on the palatal area, illustrating measurements used herein. Images are not to scale. (a) *Cycloderma aubryi* NMB 18041, (b) *Apalone spinifera* TMM 3817, (c) *Nilssonia formosa* NHMW 38631. Images are not to scale. *a* median anteroposterior length of triturating surface, as defined by the rhamphotheca; *b* median anteroposterior length of foramen intermaxillare; *c* width of tongue groove at midpalate, as defined by the rhamphotheca; *d* width of jaw at midpalate, as defined by the labial ridge; *e* median anteroposterior length of space between posterior margin of foramen intermaxillare and anterior edges of internal nares; *f* median anteroposterior distance from the anterior margin of triturating surface to the anterior margin of choanae; *g* median anteroposterior distance from the anterior margin of triturating surface to the posterior margin of narial passage; *h* sagittal anteroposterior length of palatine within internal choana; *i* sagittal anteroposterior length of internal choana; *j* perpendicular width of triturating surface, as defined by the rhamphotheca, at the anterior third; *k* perpendicular width of triturating surface, as defined by the rhamphotheca, at the posterior third; *l* median anteroposterior length of the intermaxillary contact; *m* median anteroposterior distance from the anterior contact of palatine with the vomer to the posterior terminus of the vomer; *n* median anteroposterior distance from the anterior contact of palatine with the vomer to the posterior terminus of the palatine.

*Definition*—Anteroposterior length of the foramen intermaxillare (Figure 3a) relative to the anteroposterior length of the triturating surface, as defined by the rhamphotheca (Figure 3a) (modified from Meylan, 1987, 74): 0 = 0–10% (Figure 2b); 1 = 10–20% (Figure 2f); 2 = 20–30% (Figure 2d); 3 = >30% (Figure 2e).

*Comment*—This character addresses the relative size of the foramen intermaxillare. It is based on a character of Meylan (1987, 74), who used the length of the intermaxillary foramen relative to the length of the “primary palate,” but did not clarify what exact measurement he used. We use the anteroposterior length of the triturating surface, as defined by the rhamphotheca (Figure 3a), instead of the “primary palate,” as the length of the primary palate, if conceived as the distance between the anterior margin of the triturating surfaces and the internal choanae (see Gaffney, 1979, fig. 199c), strongly depends on the length of the foramen intermaxillare, as it is included in the measurement. The rhamphotheca is not preserved in the vast majority of specimens, but typically leaves clear traces on the ventral surface of the skull. Polymorphism is scored implementing our standard protocols (see Section 2). Interestingly, even though we utilize other measurements than those of Meylan (1987), our results strongly correlate with his, with apalonines and *Trionyx triunguis* having the largest intermaxillary foramen (e.g., Figures 2d,e and 3b) and *Chitra* spp. and *Cyclanorbis elegans* the smallest (Figure 2a–c). This character is ordered.

Character 5: Foramen intermaxillare 2

*Definition*—Contribution of the vomer to the posterior margin of the foramen intermaxillare (modified from Meylan, 1987, 49): 0 = absent (Figure 2d); 1 = present (Figure 2e).

*Comment*—This character describes the absence (Figure 2b,d,f) versus presence (Figure 2a,c,e) of a vomer contribution to the posterior margin of the foramen intermaxillare. The vomer consistently does not participate in this margin in amydines, *Cyclanorbis elegans*, *Cycloderma aubryi*, *Lissemys ceylonensis*, *Lissemys scutata*, and *Trionyx triunguis*. A contribution is consistently present in *Apalone* spp., *Chitra indica*, *Cyclanorbis senegalensis*, *Pelochelys* spp., and *Rafetus euphraticus*. All other species exhibit both character states, but we only score them polymorphic if a given character state occurs in at least 20% of the population. Our results broadly replicate those of Meylan (1987). A contribution of the vomer to the foramen intermaxillare somewhat depends on the length of the intermaxillary contact (see Character 7 below), but our observations suggest that these characters do not correlate tightly.

Character 6: Primary palate 1

*Definition*—Anteroposterior length of the bony palate (Figure 3b) relative to the triturating surface (Figure 3a) (new character): 0 = 0–20% (Figure 2a); 1 = 20–40% (Figure 2c); 2 = >40%.

**FIGURE 4:**
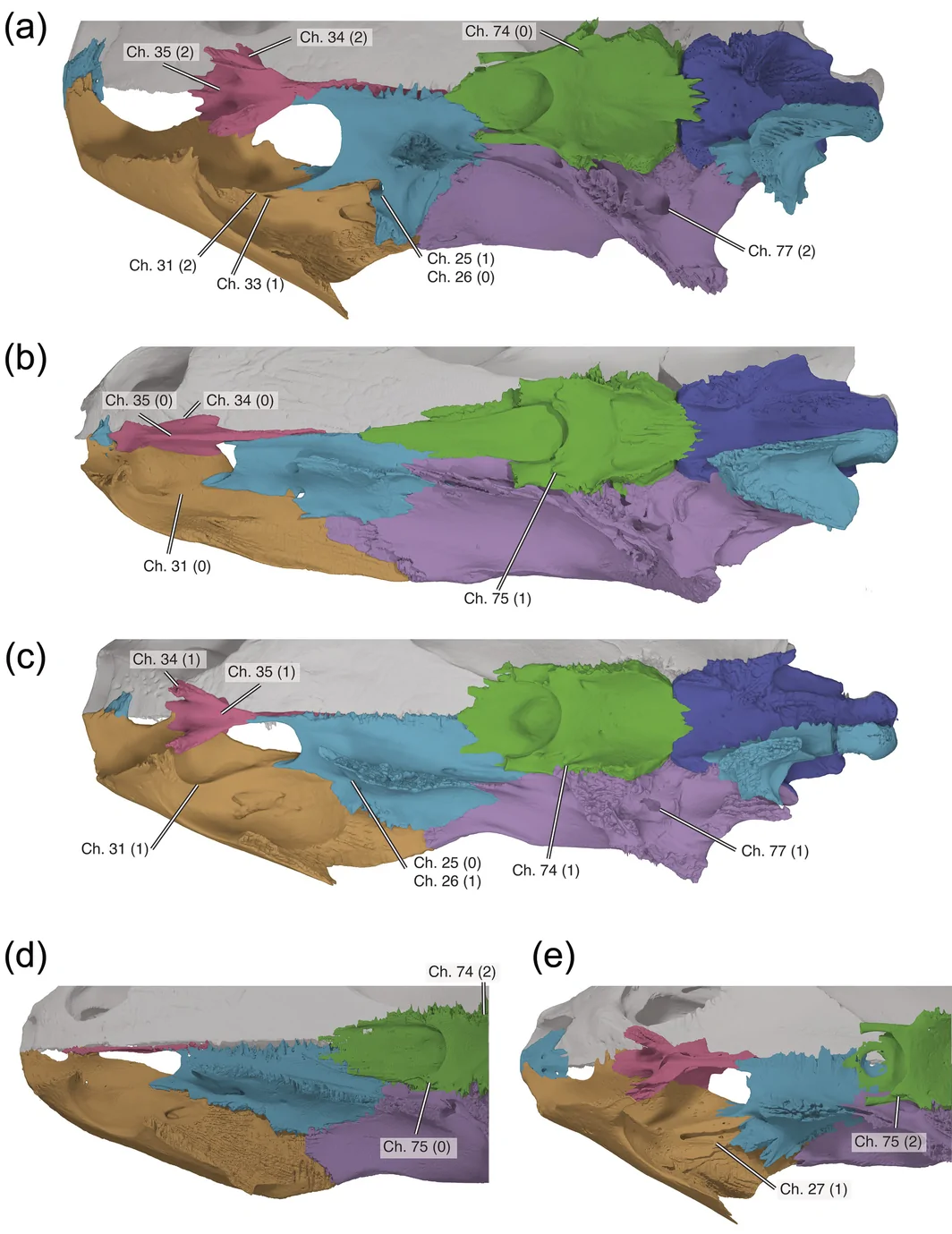
3D models of the internal anatomy of crania of select trionychid species in dorsal view illustrating character states. (a) *Rafetus euphraticus* NHMW 132, (b) *Chitra chitra* PCHP 4995, (c) *Lissemys ceylonensis* NKMB 2398, (d) *Cycloderma frenatum* NHMUK 84.2.4.1, (e) *Nilssonia formosa* NHMW 38631. Images are not to scale.

*Comment*—We establish a new character that measures the length of the “bony palate” relative to the length of the triturating surface, as defined by the rhamphotheca (Figure 3a). The bony palate is defined as the space between the posterior margin of the foramen intermaxillare and the anterior margin of the internal choanae (Figure 3b). Although the term “bony palate” is used in other groups of animals to refer to other structures, we chose this term for a lack of better alternatives. As conceived, this measure is not the same as the “primary palate” (see Character 4 above). Polymorphisms are scored implementing our standard protocols (see Section 2). Among extant species, amydines and chitrines have the longest bony palate (Figures 2c,d,f and 4c), while apalonines and cyclanorbines have the shortest (Figures 2a,b,e and 4a,b). The bony palate is extremely long in some fossil trionychids, particularly representatives of the Late Cretaceous to Paleogene *Gilmoremys/Plastomenus* lineage (Evers et al., 2023; Joyce et al., 2016; Joyce & Lyson, 2011). This character is ordered.

Character 7: Primary palate 2

*Definition*—Anteroposterior length of the intermaxillary contact in the bony palate (Figure 3b) relative to the triturating surface as defined by the rhamphotheca (Figure 2a) (modified from Meylan, 1987, 48): 0 = contact absent (Figure 2e); 1 = <10%; 2 = 10–20% (Figure 2b); 3 = 20–30%; 4 = >30–40% (Figure 2d); 5 = >40%.

*Comment*—This character utilizes the relative length of the contact of the maxillae with its counterpart in the bony palate (see Character 6 for definition) relative to the length of the triturating surface as defined by the rhamphotheca (Figure 3). The intermaxillary contact always forms ventral to the vomer. The median of the bony palate is thus formed by the vomer whenever this contact is absent, an observation utilized by Meylan (1987, 48) as the basis of his character. The presence and length of the intermaxillary contact are highly variable in trionychids, but we observe trends that appear to be both driven by taxonomy and feeding mechanism. Polymorphism is scored implementing our standard scoring protocol (see Section 2). *Pelochelys cantorii* is the only species that consistently lacks an intermaxillary contact in the bony palate (not figured). The greatest intermaxillary contacts among extant trionychids are found in amydines and *Trionyx triunguis* (Figures 2d,f and 4c). Some fossil trionychids, in particular plastomenids, show even greater values (Evers et al., 2023; Joyce et al., 2016; Joyce & Lyson, 2011). The length of the intermaxillary contact is related to, but does not fully correlate with the relative length of the bony palate (see Character 6 above). This character is ordered.

Character 8: Palatal groove 1

*Definition*—Width of the palatal groove (=“tongue groove”) at mid bony palate (Figure 3c) relative to width of triturating surface at mid bony palate (Figure 3c) (new character): 0 = 0–20% (Figure 2b); 1 = 20–40% (Figure 2f); 2 = 40–60%; 3 = >60% (Figure 2c).

*Comment*—This character makes use of the relative width of the palatal groove (=tongue groove) taken at mid length of the bony palate (see Character 6 for definition) relative to the width of the triturating surfaces, also measured at mid bony palate (Figure 3c). The palatal groove is a distinct space in the skull of most trionychids that is partially roofed by the bony palate and laterally defined by the margins of the rhamphotheca. This space accommodates a buccal lining involved in the pharyngeal respiration (Gage & Gage, 1885; Pritchard, 1979). *Pelochelys* spp. and *Chitra* spp. have very wide, but flat palatal grooves (Figure 2c), while *Cyclanorbis elegans* and *Cycloderma frenatum* have the tightest palatal grooves (Figure 2a,b). *Pelodiscus* sp. is found to be especially polymorphic as it covers the whole spectrum of variation seen for this character. This is either perhaps because this taxon encompasses several distinct species with different feeding adaptations or because this species complex shows more variation due to its partial domestication (see Section 3). This character is ordered.

Character 9: Narial passage 1

*Definition*—Anteroposterior position of the anterior limit of the internal choanae (Figure 3c) relative to the triturating surface (Figure 3a) (new character): 0 = 0–30% (Figure 2a); 1 = 30–60% (Figure 2c); 2 = >60% (Figure 2d).

*Comment*—This character measures the anteroposterior length between the anterior margin of the triturating surfaces and the anterior margin of the internal choanae (Figure 3c) relative to the anteroposterior length of the triturating surfaces as defined by the rhamphotheca (Figure 3a) as a proxy to assess the anteroposterior position of the narial passage relative to the palate. Therefore, the higher the scoring, the more posteriorly the internal choanae are located. Polymorphism is addressed using our standard scoring methods (see Section 2). The anterior edge of the internal choanae is found to be located the most anteriorly in cyclanorbines (Figures 2a,b and 4a) and the most posteriorly in amydines and *Trionyx triunguis* (Figures 2d,f and 4c). This character is ordered.

Character 10: Narial passage 2

*Definition*—Anteroposterior position of the posterior limit of the bony narial passage (Figure 3c) relative to the triturating surface (Figure 3a) (new character): 0 = 0–60% (Figure 2c); 1 = 60–80% (Figure 2e); 2 = 80–100% (Figure 2d); 3 = >100% (Figure 2f).

*Comment*—This character measures the length between the anterior margin of the triturating surfaces and the posterior margin of the bony narial passage, as defined by the rounded marks left by the narial passage in the palate (Figure 3c), relative to the triturating surface, as defined by the rhamphotheca (Figure 3a). This serves as an additional proxy for assessing the anteroposterior position of the narial passage relative to the palate (also see Character 9 above). Higher values correspond to more posteriorly located bony narial passages. Indeed, as the posterior limits of the bony narial passages can be located behind the triturating surfaces, some taxa have values greater than 100%. Polymorphism is scored implementing our standard methods (see Section 2). Among extant trionychids, the posterior limit of the bony narial passage is located the furthest anteriorly in *Chitra* spp., *Cycloderma* spp., *Pelochelys* spp., and *Lissemys ceylonensis* (Figures 2a,c and 3a), and the furthest posteriorly in *Amyda* spp. (Figure 2f). The relative position of the anterior (see Character 9 above) and posterior limits of the narial passage is tightly related, but does not correlate fully. This character forms a morphocline that can be ordered.

Character 11: Narial passage 3

*Definition*—Anteroposterior contribution of the palatine to the roof of the posterior narial passage (Figure 3a) (new character): 0 = <50% (Figure 3b); 1 = 50–75% (Figure 2e); 2 = >75% (Figure 2c).

*Comment*—This character utilizes the relative anteroposterior contribution of the palatine to the roof of the internal choanae relative to the anteroposterior length of the internal choanae (Figure 3). As the roof of the posterior narial passage is only formed by the palatine, the remainder being a fenestra that communicates with the orbits, this character allows assessing the level of ossification to this portion of the narial canal. As with the previous character, polymorphism is scored implementing our standard scoring method (see Section 2). The posterior narial passage is the least ossified by the palatine in apalonines (Figures 2e and 3b). The greatest values are seen in *Chitra indica* (Figure 2c). This character is ordered.

Character 12: Narial passage 4

*Definition*—Free medial flange of the maxilla flooring the narial passage (new character): 0 = absent (Figure 2f); 1 = present (Figure 2a).

*Comment*—This character pertains to the presence of a medially oriented sheet of bone that is formed by the maxilla and that partially floors the narial passage. This flange is only found in *Chitra* spp., *Cycloderma* spp., and *Pelochelys* spp. (Figures 2a,c and 3a). With minor exceptions, polymorphism is not apparent for this character.

Character 13: Vomer 1

*Definition*—Anteroposterior extension of the vomer relative to the anteroposterior length of the palatine (modified from Meylan, 1987, 51): 0 = 0–20% (Figure 2a); 1 = 20–40% (Figure 2d); 2 = 40–60% (Figure 2c); 3 = 60–80% (Figure 2e); 4 = 80–100%.

*Comment*—This morphometric character attempts to capture how far posteriorly the vomer protrudes between the main body of the palatines. For this purpose, we measure the median anteroposterior distance from the anterior contact of the palatine with the vomer to the posterior terminus of the vomer (Figure 3b) over the median anteroposterior distance from the anterior contact of the palatine with the vomer to the posterior terminus of the palatines (Figure 3b). As the primary observations of most species span our thresholds, most taxa score polymorphically following our protocols (see Section 2), but we observe trends nonetheless. In particular, cyclanorbines typically exhibit low values, meaning that the vomer does not extend far posteriorly between the palatines (Figures 2a,b and 3a). In contrast, the vomer reaches the farthest posteriorly between the palatines in *Rafetus* spp., *Pelodiscus* sp., and *Pelochelys* spp. (Figure 2e). Indeed, in two‐thirds of our sample of *Pelochelys bibroni* and half of our sample of *Rafetus euphraticus*, the vomer separates the palatines fully to posteriorly contact the pterygoids or parabasisphenoid (not figured). The latter contact was found by Meylan (1987, 51) to be an autapomorphy of *Pelochelys bibroni*. This character is ordered.

Character 14: Vomer 2

*Definition*—Flat anteroventral extension of the vomer at the contact with the maxillae (new character): 0 = absent (Figure 2a); 1 = present (Figure 2e).

*Comment*—This character documents the presence of an anteroventral extension of the vomer in the palate, which forms a flat surface that contrasts with the remaining, narrow, rod‐like portion of the bone. Polymorphism is scored implementing our standard scoring method (see Section 2). An anteroventral extension of the vomer is found in most trionychids (Figure 2b–f), is polymorphically absent in *Lissemys punctata*, *Palea steindachneri*, and *Trionyx triunguis*, and consistently absent in *Cycloderma* spp. (Figures 2a and 3a).

Character 15: Vomer 3

*Definition*—Foramina on the ventral surface of the vomer (new character): 0 = absent (Figure 2d); 1 = present (Figure 2f).

*Comment*—One or multiple foramina are variably developed on the flat anteroventral extension of the vomer (see Character 14). Polymorphism is scored implementing our standard scoring method (see Section 2). These foramina are absent in all apalonines, chitrines, and cyclanorbines (Figure 2b–e), but are variably present in amydines, particularly *Amyda* spp. (Figure 2f).

Character 16: Foramen Palatinum Posterius 1

*Definition*—Contribution of the maxilla and/or pterygoid to the foramen palatinum posterius (reworded from Meylan, 1987, 54): 0 = absent (Figure 2b); 1 = present (Figure 2e).

*Comment*—Meylan (1987) developed a character that distinguishes between the formation of the foramen palatinum posterius by the palatine only or by the palatine in conjunction with the maxilla and/or pterygoid (Figure 2). We reworked this character to only pertain to a contribution of the maxilla to the formation of the foramen palatinum posterius as preliminary scorings reveal that a contribution of the pterygoid to the foramen occurs only sporadically at a rate far below our threshold of 20%. Right/left asymmetry is commonly found and is reported as polymorphic for the affected specimens. Polymorphism is scored using our standard scoring method, but the sample size doubled by regarding the right and left side as independent data points (see Section 2). In cyclanorbines, the foramen palatinum posterius is formed by the palatine only (Figures 2a,b and 3a). The maxilla contributes consistently to the foramen in most trionychines (Figures 2c–f and 3b,c), but an eclectic sample scores polymorphic.

Character 17: Foramen Palatinum Posterius 2

*Description*—Number of foramina palatinum posterius per side (modified from Meylan, 1987, 53): 1 = one foramen (Figure 2d); 2 = two foramina (Figure 2b); 3 = three or more foramina (Figure 2a).

*Comment*—The foramen palatinum posterius of trionychids is small compared to other turtles (Meylan, 1987) and uniquely serves as the posterior opening to the canalis intrapalatinus (Albrecht, 1967; Ogushi, 1911). Meylan (1987) proposed a character for trionychids that distinguishes between the presence of a single, small foramen versus a divided, small foramen, or several small openings. As we approach polymorphism by first scoring individuals separately, we rephrased this character as pertaining to the number of foramina apparent in a given specimen. This character is highly polymorphic in some species and often shows right/left asymmetry, which is reported as polymorphic for a given specimen (e.g., Character 74; Figure 4c). As with the previous character, polymorphism is scored implementing our standard scoring method, but our sample is doubled by regarding the right and left sides of the skull as separate data points (see Section 2). While *Amyda* spp., *Apalone spinifera*, *Dogania subplana*, *Palea steindachneri*, *Pelochelys* spp., *Pelodiscus* sp., *Rafetus* spp., and *Trionyx triunguis* consistently have one foramen (Figures 2d,e and 3b), *Chitra indica*, *Lissemys* spp., and *Nilssonia leithii* exhibit two foramina (Figure 2c), and *Cycloderma frenatum* has three or more (Figure 2a). *Cyclanorbis elegans* is found to be highly variable showing all three character states. This character is ordered.

Character 18: Canalis intrapalatinus 1

*Description*—Length of the canalis intrapalatinus (new character): 0 = canal reduced to a fenestra; 1 = canal well formed (Figure 2).

*Comment*—In trionychids, the foramen palatinum posterius of other turtle groups is extended into a bony canal, the canalis intrapalatinus (Albrecht, 1967; Ogushi, 1913), which is formed by the palatine with occasional contributions from the maxilla (see Character 19 below). The foramen palatinus posterius and the foramen palatinus anterius are the posterior and anterior openings to this canal, respectively. The canalis intrapalatinus houses the intrapalatine artery and the anterior opening of the canalis nervus vidianus (Ogushi, 1913; Rollot et al., 2018). The canal is secondarily reduced to a fenestra in *Pelochelys* spp. The foramina palatinus posterius and palatinus anterius are thus essentially confluent (not figured, but clearly seen in 3D models).

Character 19: Canalis intrapalatinus 2

*Description*—Contribution of the maxilla and/or pterygoid to the canalis intrapalatinus (new character): 0 = absent; 1 = present.

*Comment*—The maxilla and/or pterygoid variably contribute to the formation of the canalis intrapalatinus in trionychids. This character can easily be assessed in 3D models by removing the maxilla and observing if the canalis intrapalatinus is fully formed by the palatine (not figured). Our observations indicate that this character partly correlates with Character 16 and Character 25, but not fully. The canalis intrapalatinus is only formed by the palatine in cyclanorbines, *Apalone ferox*, *Apalone spinifera*, and *Pelochelys bibroni*. This character is developed polymorphically in *Amyda cartilaginea*, *Dogania subplana*, *Pelochelys cantorii*, and *Pelodiscus* sp. A contribution of the maxilla and/or pterygoid is otherwise found in all other trionychines.

Character 20: Foramen palatinum accessorium 1

*Definition*—Number of foramina palatinum accessorium (new character): 1 = one foramen developed (Figure 2d); 2 = two or more foramina developed (Figure 2a).

*Comment*—We herein use the nomenclature introduced by Albrecht (1967) to refer to foramina located on the ventral surface of the palatines of trionychids and connected to the canalis intrapalatinus as the foramina palatinum accessorium (see Character 18; Figure 2). We find much polymorphism for this character, including right/left asymmetry, which is reported as polymorphic for affected specimens and counted as two different data points for the species' final scoring (see Section 2). In *Pelodiscus bibroni*, *Chitra* spp., and *Cycloderma* spp., gap suction feeders with elongated skulls, several foramina are developed consistently (Figure 2a,c). This character is otherwise developed highly polymorphically in other trionychid species.

Character 21: Triturating surface 1

*Definition*—Relative width of the triturating surface at its posterior third (Figure 3c) relative to its anterior third (Figure 3c) (new character): 0 = <100%, triturating surface clearly narrowing towards the posterior (Figure 2c); 1 = 100–200%, triturating surface moderately widening towards the posterior (Figure 2e); 2 = >200%, triturating surface strongly widening towards the posterior.

*Comment*—This character captures the shape of the triturating surface of trionychids as either posteriorly narrowing, posteriorly expanding moderately, or posteriorly expanding strongly. To score this character, we measured the perpendicular width of the triturating surface, as defined by the rhamphotheca, at the posterior third of the length of the rhamphotheca and the perpendicular width of the triturating surface at the anterior third of the rhamphotheca (Figure 3). Polymorphism is scored implementing our standard protocols (see Section 2). Most trionychids have triturating surfaces that expand posteriorly moderately (Figures 2b,d–f and 3b,c). Triturating surfaces that expand posteriorly strongly are found in *Dogania subplana*, *Nilssonia leithii*, and *Palea steindachneri*. A posterior narrowing of the triturating surface is found in suction feeders with long skulls, in particular *Chitra* spp., *Cycloderma aubryi*, and *Pelochelys* spp. (Figures 2a,c and 3a). This character is ordered.

Character 22: Triturating surface 2

*Definition*—Labial ridge (new character): 0 = highly reduced to absent (Figure 3a); 1 = present and distinct (Figure 2d).

*Comment*—This character utilizes the absence or presence of a labial ridge (Figures 2 and 3). Among extant trionychids, only *Pelochelys* spp. and *Cycloderma aubryi* have a flat triturating surface lacking a labial ridge (Figure 3a, but best seen in the 3D models). Polymorphism is not apparent for this character.

Character 23: Triturating surface 3

*Definition*—Presence of an accessory ridge in the upper triturating surfaces (Joyce & Lyson, 2011, 79): 0 = absent (Figure 2b); 1 = present (Figure 2c).

*Comment*—This character scores the variable presence of an accessory ridge on the triturating surface of the maxilla (Figure 1c). In extant species, this anatomical feature, running along the full length of the triturating surface, is only found in *Chitra* spp. However, among fossil trionychids, accessory ridges are also observed in the plastomenid *Gilmoremys lancensis*, though they are much shorter than in *Chitra* spp. (Joyce et al., 2016; Joyce & Lyson, 2011).

Character 24: Triturating surface 4

*Definition*—Participation of the pterygoid in the triturating surface, as defined by the rhamphotheca (new character): 0 = absent (Figure 2e); 1 = present (Figure 2a).

*Comment*—This character addresses the variable participation of the pterygoid to the triturating surfaces as defined by the rhamphotheca. Polymorphism is scored using our standard methods (see Section 2). A participation of the pterygoid to the triturating surface is found consistently in amydines and cyclanorbines (Figures 2a,b,f and 3c), except for *Cycloderma aubryi* (Figure 3a), which is scored polymorphic. Apalonines and chitrines consistently lack a participation of the pterygoid to the triturating surface (Figures 2c–e and 3b).

Character 25: Foramen palatinum anterius 1

*Definition*—Maxilla contribution to the foramen palatinum anterius (new character): 0 = absent (Figure 4c); 1 = present (Figure 4a).

*Comment*—This character documents the variable contribution of the maxilla to the foramen palatinum anterius (Girard et al., 2024), which is the anterior opening of the canalis intrapalatinus housing the inframaxillary artery (Albrecht, 1967). This character can only be observed in pictures taken with good lighting at a specific angle, but images with these qualities are rare in our sample. Our scoring is therefore mostly restricted to specimens for which we have CT scans (Figure 4). As the resulting sample size is small and unrepresentative, we retained all observed polymorphism instead of implementing a 20% cutoff. This character correlates partially, but not fully, with other characters pertaining to the canalis intrapalatinus (see Characters 18 and 19 above). The maxilla contributes consistently to the foramen palatinum anterius in *Apalone ferox*, *Nilssonia formosa*, *Nilssonia nigricans*, and *Rafetus euphraticus* and polymorphically in *Chitra chitra*, *Pelochelys cantorii*, and *Trionyx triunguis* (Figure 4a,b). In the other species scored, the maxilla does not participate in the foramen palatinum anterius (Figure 4c,d). As presented, this character does not appear to have a meaningful phylogenetic signal. We retain it, nonetheless, as it may help place fossils in the tree, but suggest reevaluating it in the future by reference to a larger sample.

Character 26: Foramen palatinum anterius 2

*Definition*—Position of foramen palatinum anterius (new character): 0 = lateral to secondary braincase wall (Figure 4a); 1 = within secondary braincase wall (Figure 4c).

*Comment*—This character pertains to the position of the foramen palatinum anterius relative to the secondary wall of the braincase, which is formed by the palatine and parietal in trionychids. Polymorphism is scored implementing our standard protocols (see Section 2). The foramen palatinum anterius is found lateral to the secondary wall of the braincase (Figure 1d) in most trionychines and *Cycloderma* spp. (Figure 4a,b) and at the base of, or medial to, the secondary braincase wall (Figure 4c) in the remaining cyclanorbines and the small‐sized trionychines *Apalone mutica* and *Palea steindachneri*. It is expressed polymorphically in *Amyda cartilaginea*, *Apalone ferox*, *Apalone spinifera*, and *Pelodiscus* sp.

Character 27: Foramen palatinum anterius 3

*Definition*—Confluence of the foramen palatinum anterius with a groove leading to the foramen supramaxillare (new character): 0 = absent (Figure 4a–c); 1 = present (Figure 4d,e).

*Comment*—This character describes a condition in which the foramen palatinum anterius is connected to the foramen supramaxillare by a sulcus running in the floor of the orbit. According to Albrecht (1967), the infraorbital artery splits in the floor of the orbit into the posteriorly directed inframaxillary artery, which supplies the canalis intrapalatinus, and the anteriorly directed alveolar‐nasal arteries, which supply the foramina supramaxillare and alveolare superius. In some species of trionychids these arteries leave a sulcus in the bone that connects the foramen palatinum anterius and the foramen supramaxillare. Within our sample, this condition is consistently observed in *Chitra indica*, *Cyclanorbis elegans*, *Cycloderma frenatum*, and *Lissemys punctata*, (Figure 4d), but occurs polymorphically in *Lissemys ceylonensis* and *Nilssonia* spp. (Figure 4e). A canal is absent in the remainder of our sample.

Character 28: Crista cranii

**FIGURE 5:**
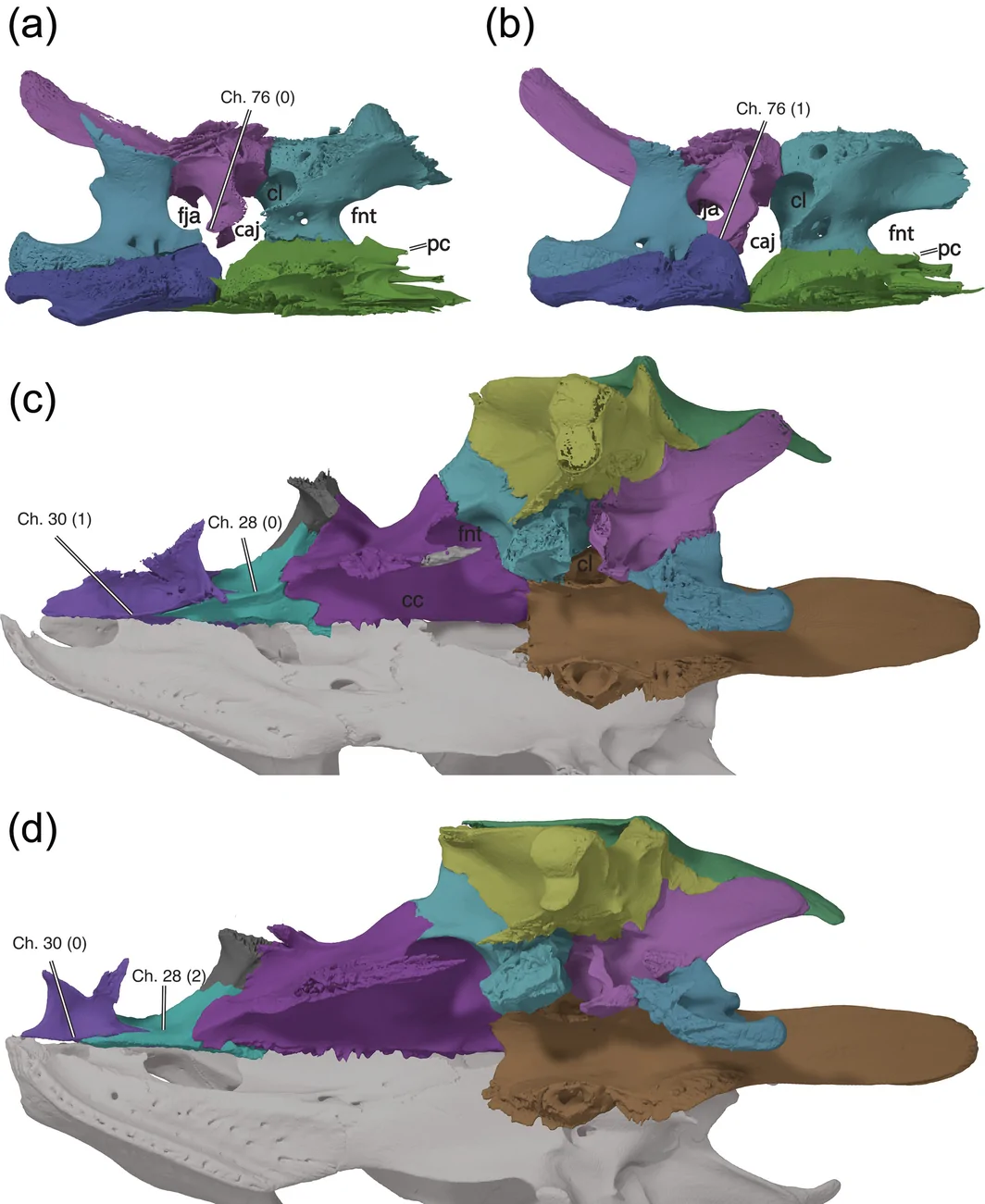
3D models of the internal anatomy of crania of select trionychid species illustrating character states with focus on the area around hiatus acusticus in lateral (a, b) and ventral view (c, d). (a) *Rafetus euphraticus* NHMW 132, (b) *Nilssonia formosa* NHMW 38631, (c) *Trionyx triunguis* PCHP 4559, (d) *Cyclanorbis senegalensis* PCHP 2627. Images are not to scale. *caj* cavum acustico jugulare, *cc* cavum cranii, *cl* cavum labyrinthicum, *fja* foramen jugulare anterius, *fnt* foramen nervi trigemini, *pc* processus clinoideus.

*Definition*—Crista cranii and sulcus olfactorius (modified from Evers & Benson, 2019, 14): 0 = distinct crista cranii present that laterally define a distinct median sulcus olfactorius (Figure 5a,b); 1 = sharp ridges present that frame a flat median surface, instead of a sulcus; 2 = frontals converge medially to form a rounded median ridge (Figure 5c).

*Comment*—This character utilizes the varying presence and definition of the crista cranii and sulcus olfactorius. The crista cranii is a pair of anteroposteriorly oriented ridges that are formed by the frontal and parietal and located between the orbits. The sulcus olfactorius is the median sulcus formed between the ridges of the crista cranii, which connects the fossa nasalis with the cavum cranii. In most trionychines, the crista cranii forms a distinct sulcus olfactorius (Figure 5a,b). In some trionychids, the crista cranii does not phrase a sulcus, but rather a flat median surface. This condition is consistently found in *Cyclanorbis elegans* and polymorphically developed in *Apalone ferox* and *Apalone spinifera*. The crista cranii converge into a rounded, median ridge in the remaining cyclanorbines, notably *Cyclanorbis senegalensis*, *Cycloderma frenatum*, *Lissemys punctata*, and *Lissemys punctata* (Figure 5c). As the three available character states do not form a clear morphocline, this is one of the few multistate characters that we do not recommend ordering.

Character 29: Nasal cavity 1

**FIGURE 6:**
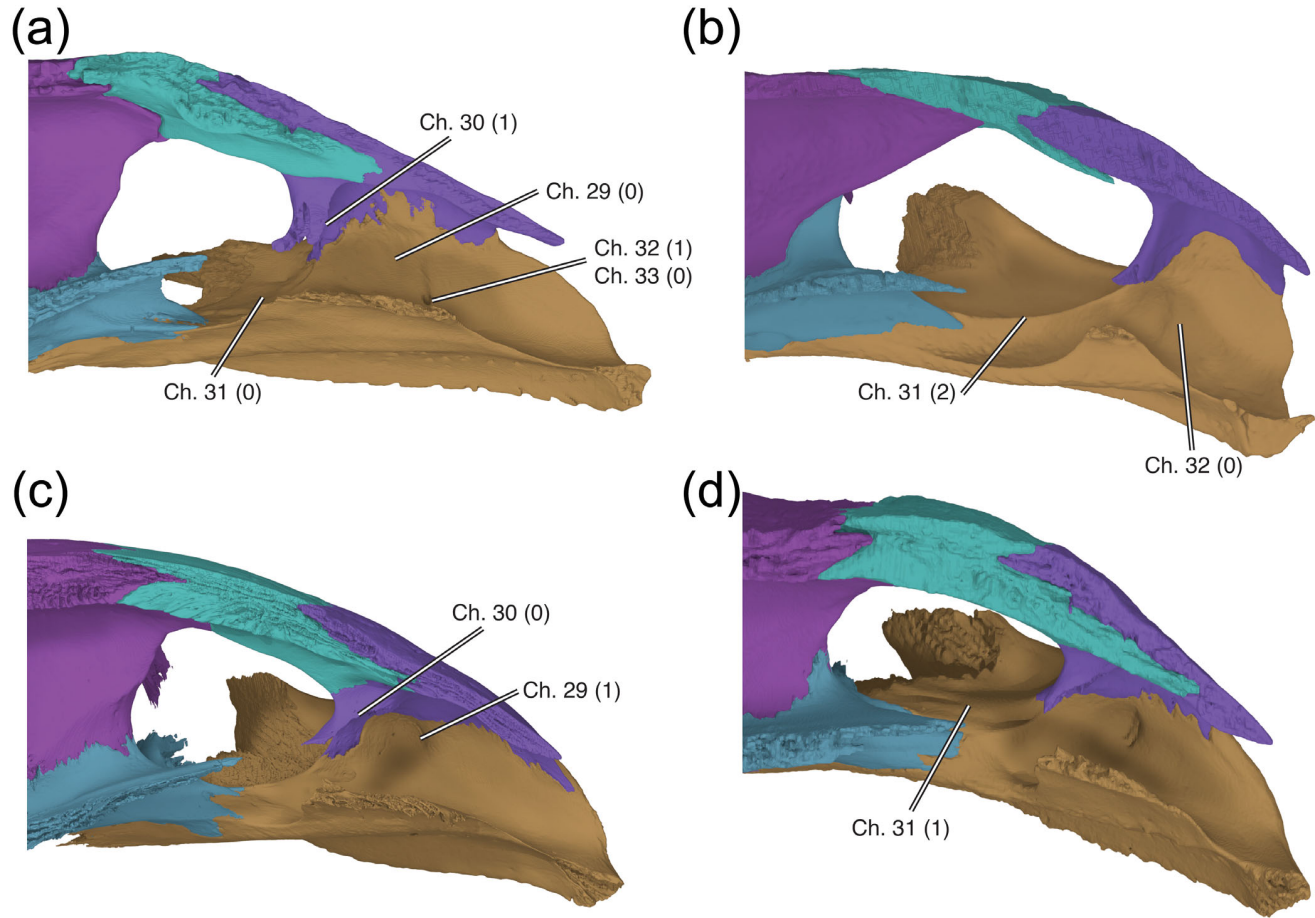
3D models of the internal anatomy of crania of select trionychid species illustrating character states with a focus on the nasal and orbital cavities in left lateral view. (a) *Trionyx triunguis* PCHP 4559, (b) *Lissemys punctata* SMF 74141, (c) *Amyda cartilaginea* FMNH 224117, *Apalone ferox* PCHP 7043 (mirrored). Images are not to scale.

*Definition*—Presence of a nasal pocket in the lateral wall of the nasal cavity (new character): 0 = absent (Figure 6a); 1 = present (Figure 6c).

*Comment*—This character addresses the varying presence of a depression in the lateral wall of the maxilla within the nasal cavity, which we name the nasal pocket (Figure 6c). The nasal pocket is notably absent in all cyclanorbines, *Chitra* spp., *Nilssonia formosa*, *Rafetus euphraticus*, and *Trionyx triunguis* but present in the remaining amydines, *Apalone* spp., and *Pelochelys* spp. Our sample is limited, as we can only observe this character in CT scans. However, this does not appear to be problematic, because we observe little polymorphism and because our observations are broadly congruent with phylogeny.

Character 30: Nasal cavity 2

*Definition*—Presence of a distinct ridge along the anterior margin of the vomerine process, medially defining a deep cavity in the roof of the nasal capsule (new character): 0 = absent (Figures 5c and 6c); 1 = present (Figures 5a,b and 6a).

*Comment*—This character documents the varying presence of a deep cavity in the roof of the nasal capsule that is created by a distinct ridge of the vomerine process of the descending process of the prefrontal of *Trionyx triunguis* (Figures 5a,b and 6a). This character is best observed with 3D models. As this character is consistently found in *Trionyx triunguis* only, we did not need to adjust the scoring for polymorphism.

Character 31: Suborbital crest 1

*Definition*—Presence and definition of the suborbital crest of the maxilla (modified from Evers et al., 2023, 98): 0 = absent (Figures 4b and 6a); 1 = present but low (Figures 4c and 6d); 2 = present and distinct (Figures 3a and 5b).

*Comment*—Evers et al. (2023) introduce a character pertaining to the variable presence of the suborbital crest, a distinct ridge formed by the maxilla in the floor of the orbit that visually separates the orbit per se from the interorbital cavity (Figures 4 and 6). As a full gradation is apparent between the two extremes, we added an additional character state that distinguishes low suborbital ridges (state 1) from sharp suborbital crests (state 2). Our primary data set is limited, as the suborbital crest is best observed in 3D models, but as polymorphism is limited, this does not seem to be a problem. Suborbital crests are mainly absent in chitrines while low and distinct ridges are distributed among the other trionychid species. This character is ordered.

Character 32: Foramen alveolare superius 1

*Definition*—Presence of the foramen alveolare superius (modified from Evers et al., 2023, 99): 0 = absent (Figure 6b); 1 = present (Figure 6a).

*Comment*—The foramen alveolare superius of trionychids is the more anterior of the two maxillary foramina found in the floor of the orbit and nasal cavity (Albrecht, 1967; Girard et al., 2024). The foramen alveolare superius is located within or anterior to the suborbital crest (Figures 1, 4, and 6; see also Character 30). Evers et al. (2023) introduced a character that variously interprets the foramen alveolare superius as being an entry foramen versus a fenestra into the canalis alveolaris superior. As we find it difficult to objectively distinguish these two character states, we combined them to refer to the simple presence of the foramen alveolare superius. The foramen alveolare superius is consistently present in trionychines (Figure 6a,c,d) and consistently absent in cyclanorbines (Figure 6b), as previously noted by Evers et al. (2023) and Girard et al. (2024). This character is best scored by reference to CT scans or cut specimen.

Character 33: Foramen alveolare superius 2

*Definition*—Location of the foramen alveolare superius (new character): 0 = anterior to suborbital crest within nasal cavity (Figure 6a); 1 = within suborbital crest (Figure 4a).

*Comment*—This character utilized the location of the foramen alveolare superius, which is the anterior‐most foramen of the pair of foramina that supply the maxilla (see Character 32 above). As this foramen is absent in cyclanorbines, they are scored inapplicable for this character. The foramen alveolar superius is located within the suborbital crest in *Apalone ferox*, *Pelochelys cantorii*, and *Rafetus* spp. (Figures 4a and 6d), and is developed polymorphically in *Pelochelys bibroni*. It is anterolaterally located in all other trionychines.

Character 34: Vomer 4

*Definition*—Contact between the vomer and prefrontal (modified from Meylan, 1987, 36): 0 = absent (Figure 4b); 1 = present, forming a slim bar (Figure 4c); 2 = present, forming a thick column (Figure 4a).

**FIGURE 7:**
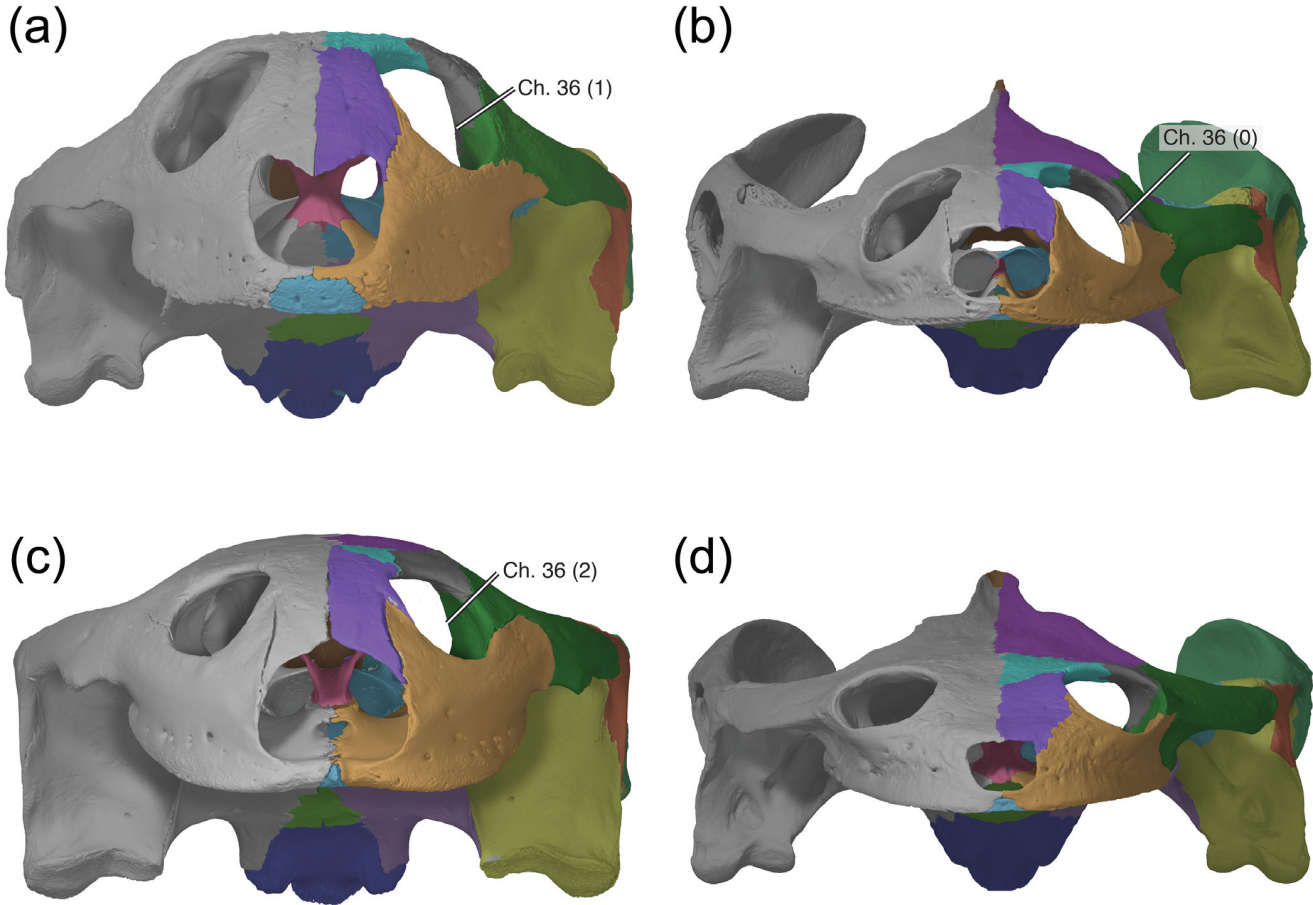
3D models of crania of select trionychid species in anterior view illustrating character states. (a) *Apalone spinifera* TMM 3817, (b) *Cycloderma aubryi* 18041, (c) *Cyclanorbis elegans* NHMW 157, (d) *Pelochelys cantorii* PCHP 4974. Images are not to scale.

*Comment*—Meylan (1987) developed a character that documents the presence or absence of a contact between the vomer and the prefrontals. This contact occurs along a dorsolaterally oriented process of the vomer contacting a medioventrally oriented process of the descending process of the prefrontals resulting in the medial enclosure of the fissura ethmoidalis. As the prefrontal obstructs the view of this contact, our figures only highlight the medioventral process of the vomer (Figure 4a–c). We added a character state to this character to evaluate the robustness of this contact. A contact of the prefrontal with the vomer is absent in *Chitra* spp., *Cycloderma* spp., and *Cyclanorbis senegalensis* (Figures 4b,d and 7b). Slim contacts are developed in the remaining cyclanorbines, while a thick contact is found in the remaining trionychines (Figure 4a,c). Some species could not be scored for this character as neither a scan nor a picture was available with the adequate angle. The only polymorphism observed for this character is found in *Palea steindachneri* in which the contact between the vomer and prefrontal is a slim bar in some specimens and a thick column in other specimens. This character forms a morphocline that can be ordered.

Character 35: Vomer 5

*Definition*—Sulcus vomeri (modified from Evers & Benson, 2019, 69): 0 = absent (Figure 4b); 1 = present, but shallow (Figure 4c); 2 = present and deep, anteriorly reaching beyond the fissura ethmoidalis (Figure 4a).

*Comment*—The sulcus vomeri is a dorsally open median groove developed on the upper surface of the vomer of turtles (Gaffney, 1972). In some species, the sulcus is particularly strong and extends anteriorly between the dorsolateral processes of the vomer to reach the nasal cavity (Figure 4a). This is notably observed in *Apalone ferox*, *Apalone mutica*, *Cyclanorbis elegans*, *Nilssonia formosa*, *Nilssonia gangetica*, *Pelochelys* spp., and *Rafetus euphraticus* (Figure 4a,e). The sulcus vomeri is absent in *Chitra* spp. and *Cycloderma* spp. (Figures 4b,d and 7b). The remaining species show an intermediate state (Figure 4c). The sample for the character is limited as it can only be scored by reference to 3D models or personal observations of specimens. We nevertheless observe polymorphism in *Dogania subplana*, which exhibits all three character states, and in *Palea steindachneri*, which shows both the shallow and deep states of the sulcus vomeri. This character can be ordered.

Character 36: Septum orbitotemporale

*Definition*—Width of the septum orbitotemporale (modified from Gaffney et al., 2006, 28): 0 = absent (Figure 7b); 1 = narrow (Figure 7a); 2 = wide (Figure 7c).

*Comment*—The septum orbitotemporale of trionychids is a median bony extension formed by the jugal and, occasionally, the postorbital that forms the lateral margin of the postorbital fenestra and separates the orbital cavity from the temporal region (Figures 1d and 7). The septum orbitotemporale is generally wide, extending medially to about half of the orbit width in frontal view, in cyclanorbines and amydines (Figure 7c) but narrow, not reaching half of the orbit width, in apalonines and chitrines (Figure 7a,d). The septum is only absent in *Cycloderma aubryi* (Figure 7b). This character forms a morphocline that can be ordered.

Character 37: Suborbital height 1

**FIGURE 8:**
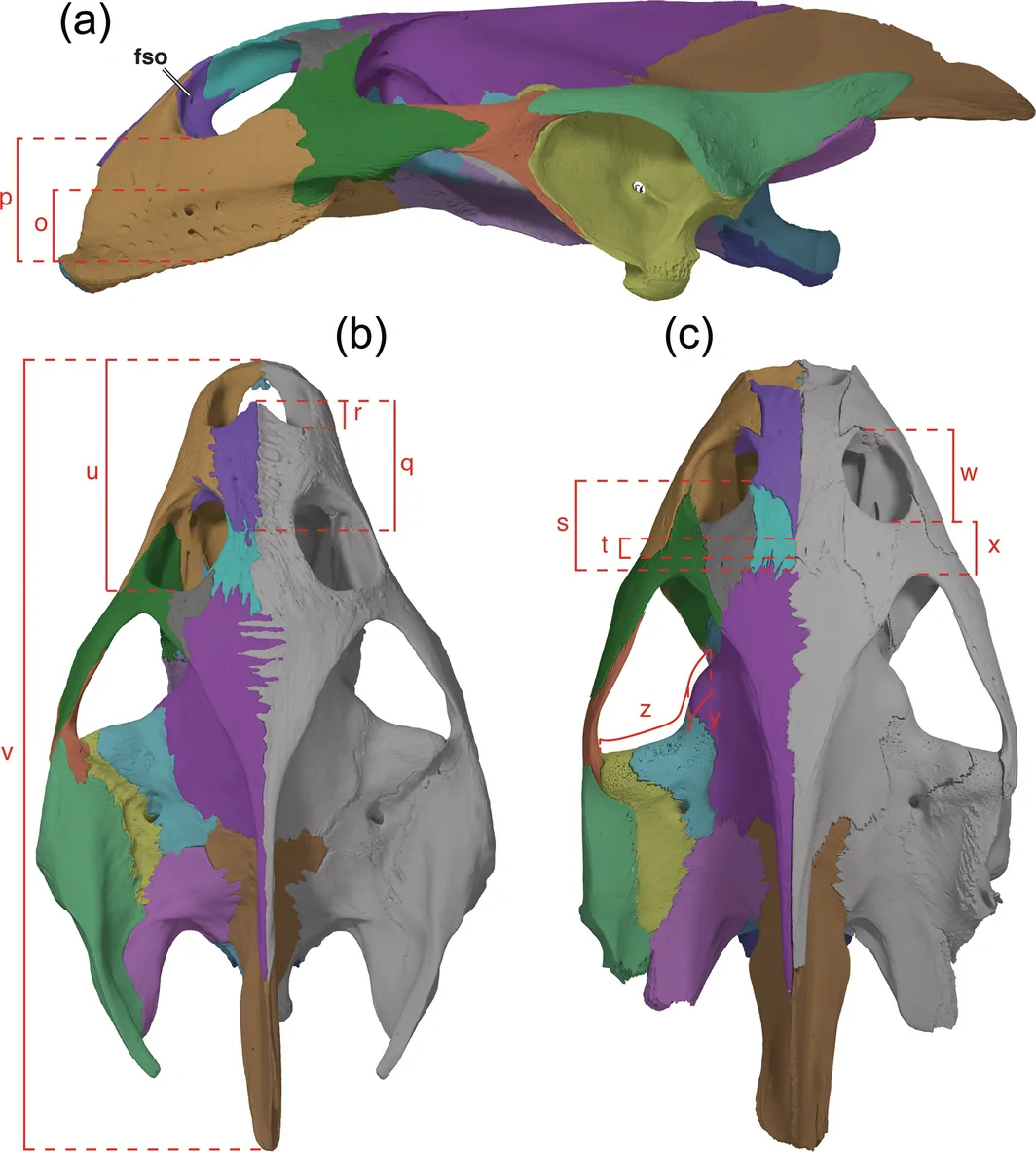
3D models of crania of select trionychid species in right lateral (a) and dorsal views (b, c) illustrating measurements used herein. (a) *Nilssonia formosa* NHMW 38631, (b) *Palea steindachneri* PCHP 7734, (c) *Cyclanorbis elegans* NHMW 157. Images are not to scale. *fso* foramen supraorbitale, *o* height of labial ridge, as defined by temporal bar and the limit of the fleshy lips, at the lowest point of the orbit; *p* height of maxilla at the lowest point of the orbit; *q* anteroposterior length of the prefrontal; *r* length of the anterior process of the prefrontal in the external nares; *s* anteroposterior length of the frontal; *t* anteroposterior length of the median contact of the frontal; *u* anteroposterior length of the face from the tip of the snout to the posterior margin of the orbit; *v* anteroposterior length of the cranium; *w* anteroposterior length of the orbit; *x* anteroposterior length of the postorbital bar; *y* parietal contribution to the anterior margin of the otic cavity; *z* length of the anterior margin of the otic cavity.

**FIGURE 9:**
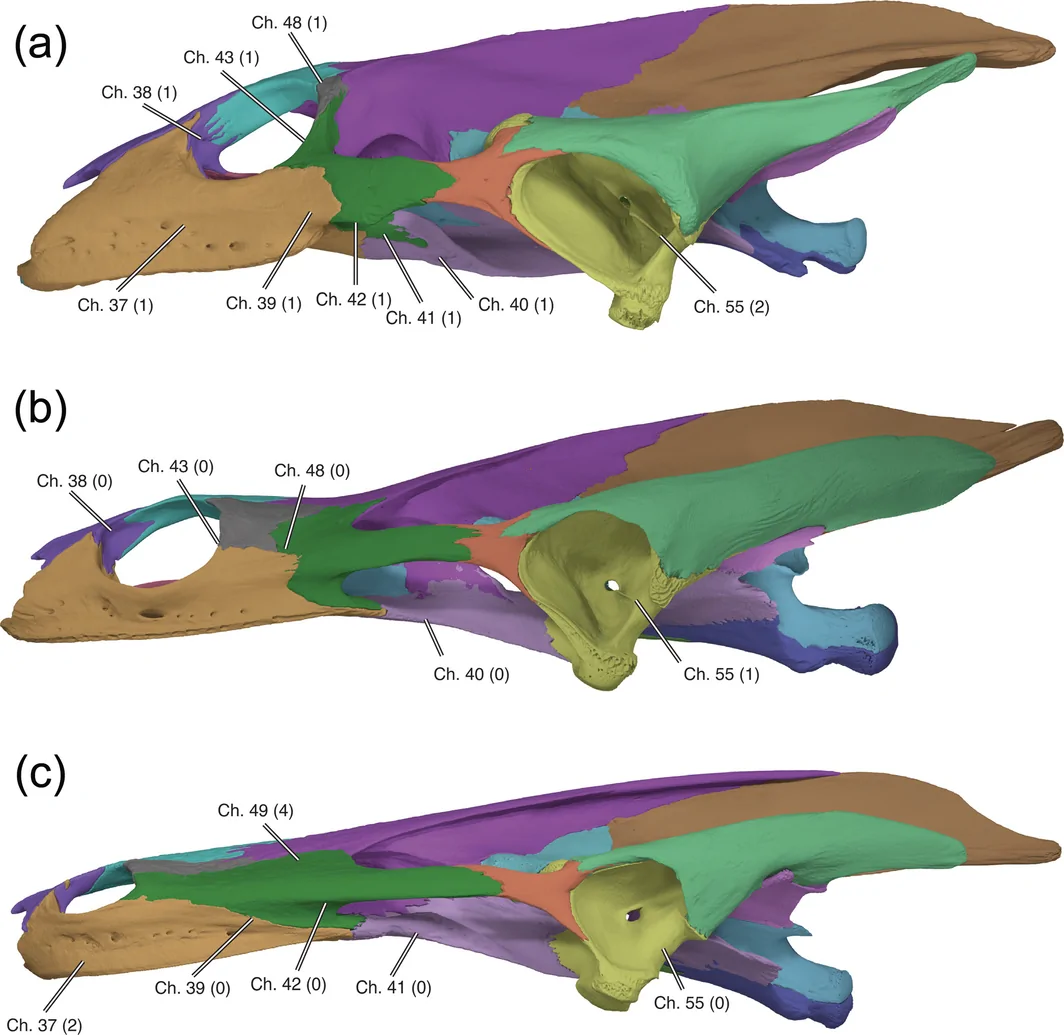
3D models of crania of select trionychid species in left lateral view illustrating character states. (a) *Dogania subplana* PCHP 3368, (b) *Cycloderma aubryi* NMB 18041, (c) *Chitra chitra* PCHP 4995. Images are not to scale.

*Definition*—Height of the labial ridge (Figure 8a) relative to the suborbital height of the maxilla (Figure 8a) (new character): 0 = <40%; 1 = 40–60% (Figure 9a); 2 = 60–80% (Figure 9c); 3 = 80–100%; 4 = >100%.

*Comment*—This morphometric character captures the relative height of the labial ridge relative to the suborbital height of the maxilla in lateral view. The labial ridge is defined for this character as the distance from the margin of the jaw to the base of the temporal bar just below the orbit (Figure 8a). The suborbital height, by contrast, is defined as the distance from the limit of the jaw to the deepest part of the orbit just below the orbit (Figure 8a). Suction feeders with long and flat skulls score notably high for this character (Figure 9b,c). *Dogania subplana* and *Rafetus swinhoei* have the lowest labial ridge while *Chitra*, and especially *Chitra indica* have the highest.

Character 38: Foramen supraorbitale

*Definition*—Foramen supraorbitale (new character): 0 = absent (Figure 9b); 1 = present (Figure 9a).

*Comment*—This character documents the variable presence of the foramen supraorbitale (Figure 8a), which, when present, is located within the prefrontal in the anterodorsal wall of the orbit (Figures 8a and 9a). We commonly observe right/left asymmetry for this character, which we score as polymorphism, and treat as separate data points (see Section 2). Caution should be utilized when using 3D models, as the visibility of this foramen is partially affected by scan and model quality. In addition, the foramen supraorbitale is sometimes present but hidden in lateral view by the anterior margin of the orbit (Figure 1g) and thus only visible in oblique views. The foramen supraorbitale is absent in *Chitra chitra* (Figure 9c), *Pelochelys* spp., and most cyclanorbines (Figure 8b,c) and found polymorphically in *Cycloderma aubryi* and *Nilssonia leithii*. It is consistently present in all other trionychines (Figures 8a and 9a).

Character 39: Jugular process 1

*Definition*—Jugular process of the maxilla (new character): 0 = absent (Figure 9c); 1 = present (Figure 9a).

*Comment*—This character addresses the variable presence of a sheet‐like, posterodorsally oriented process of the maxilla that laterally covers the jugal below or behind the orbit (Figure 9a). Much polymorphism is apparent for this character which was addressed using our standard protocols (see Section 2). A jugal process is absent in *Cycloderma aubryi* and *Chitra* spp. (Figure 9b,c), polymorphically present in *Cyclanorbis* spp. and *Pelochelys* spp., and consistently present in all other trionychids (Figure 9a).

Character 40: Vertical flange of pterygoid 1

*Definition*—Vertical flange of the pterygoid (Dryden, 1988, 18): 0 = absent (Figure 9b); 1 = present (Figure 9a).

*Comment*—The vertical flange of the pterygoid is a structure that guides the mandible during the closure of the jaw and is found in many extant cryptodires (Dryden, 1988; Joyce, 2007). In trionychids, this structure is formed by the pterygoid with a minor participation from the jugal in some species (see Character 41; Figure 9). The vertical flange is consistently absent in cyclanorbines (Figure 9b) but consistently present in trionychines (Figure 9a,c).

Character 41: Vertical flange of pterygoid 2

*Definition*—Jugal contribution to the vertical flange of the pterygoid (new character): 0 = absent (Figure 9c); 1 = present (Figure 9a).

*Comment*—This character pertains to the variable participation of the jugal to the vertical flange of the pterygoid (Figure 9). This character is scored as non‐applicable for cyclanorbines, as they lack this flange (see Character 40 above). All chitrines, *Apalone ferox*, and *Rafetus swinhoei* consistently lack a contribution of the jugal to the vertical flange of the pterygoid (Figures 1g and 9c). This character is developed polymorphically in *Apalone spinifera*, *Nilssonia gangetica*, *Nilssonia hurum*, *Nilssonia nigricans*, *Palea steindachneri*, *Pelodiscus* sp., and *Rafetus euphraticus* (Figure 9a) and is consistently present in *Amyda* spp., *Apalone mutica*, *Dogania subplana*, *Nilssonia formosa*, and *Nilssonia leithii*.

Character 42: Temporal bar 1

*Definition*—Ventral expansion of the jugal in the anterior third of the temporal bar (new character): 0 = absent (Figure 9c); 1 = present (Figure 9a).

*Comment*—The temporal bar of trionychids is mostly formed by the jugal and postorbital. This character pertains to the variable presence of a ventral expansion of the jugal (Figure 9a) that serves as the origin site for the m. zygomaticomandibularis, a muscular unit specific to Trionychia that inserts into the abductor fossa of the mandible and allows for the adduction of the lower jaw (Ogushi, 1913; Rollot et al., 2024; Schumacher, 1973; Werneburg, 2011). This expansion is absent in *Chitra* spp., *Pelochelys* spp., and *Palea steindachneri*, polymorphically present in *Apalone ferox*, *Apalone spinifera*, *Cycloderma aubryi*, *Lissemys* spp. (Figure 9b,c), and consistently present in the remainder of the sample (Figure 9a).

Character 43: Orbit 2

*Definition*—Jugal contribution to the orbit (Meylan, 1987, 67): 0 = absent, postorbital contacts maxilla along margin of orbit (Figure 9b); 1 = present (Figure 9a).

*Comment*—We here reutilize a character first developed by Meylan (1987) pertaining to the variable contribution of the jugal to the orbit margins (Figure 9). The exclusion of the jugal from the orbit sporadically occurs as a left/right asymmetry among our trionychid sample but not often enough to induce polymorphism in any species. Only *Cycloderma aubryi* shows a consistent lack of a jugal contribution to the orbit. This character is therefore reported as an autapomorphy for this species (Figure 9b).

Character 44: Face length

**FIGURE 10:**
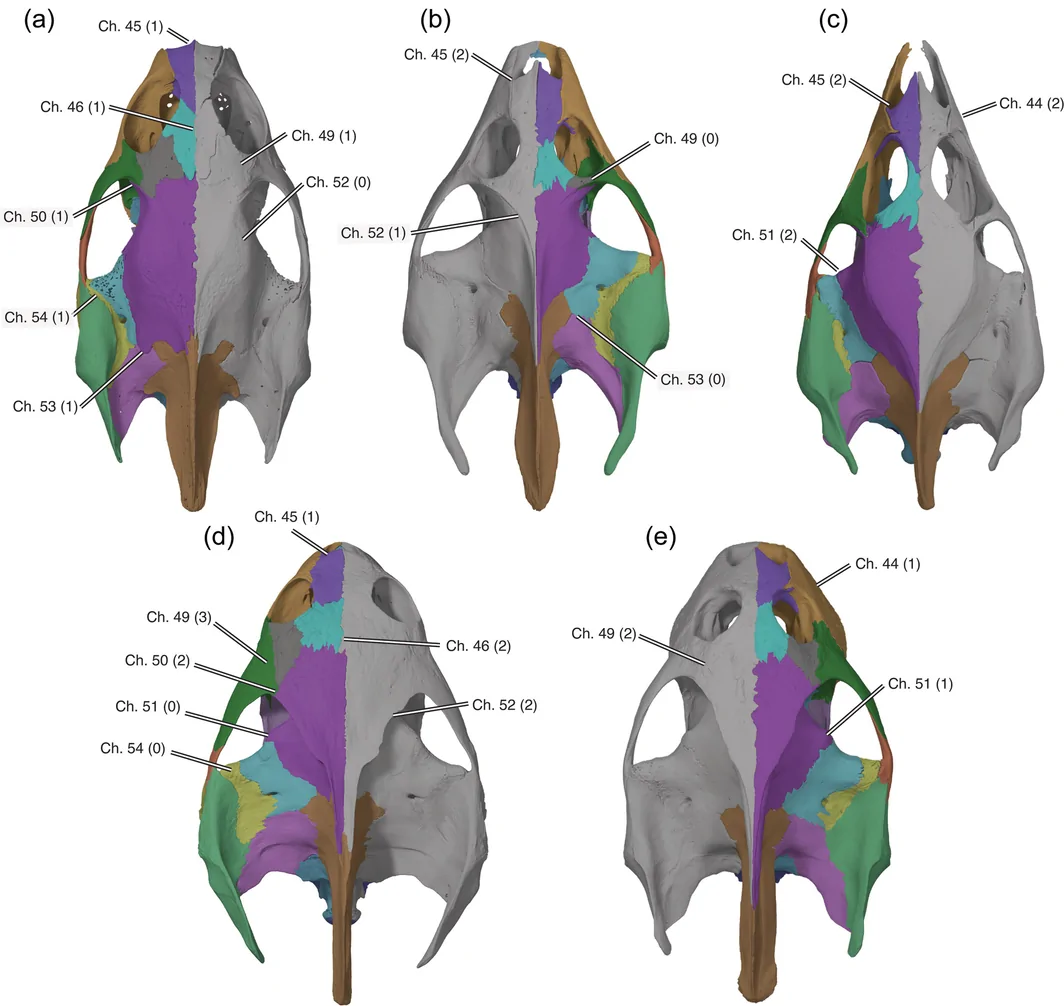
3D models of crania of select trionychid species in dorsal view illustrating character states. (a) *Lissemys punctata* SMF 74141, (b) *Dogania subplana* PCHP 3368, (c) *Apalone mutica* PCHP 2655, (d) *Pelochelys cantorii* PCHP 4974, (e) *Apalone ferox* PCHP 7043. Images are not to scale.

*Definition*—Anteroposterior length of the face (Figure 8b) relative to the anterodorsal length of the cranium in dorsal view (Figure 8b) (new character): 0 = <15% (Figure 4b); 1 = 15–30% (Figure 10e); 2 = >30% (Figure 10c).

*Comment*—This morphometric character documents the relative length of the face to the full length of the skull. The face is hereby defined as the anterodorsal length from the tip of the snout to the posterior margin of the orbit in dorsal view (Figure 8b). This character has been partially built with fossil trionychids in mind, especially *Plastomenus thomasii* and *Gilmoremys lancensis*, two plastomenids with particularly elongated snouts. Polymorphism is scored implementing our standard scoring protocol (see Section 2). *Chitra* spp. and *Cycloderma frenatum* are found to be particularly short‐snouted (Figure 4b,d). All other trionychids show an intermediate condition with some species polymorphic. Only *Apalone mutica* is clearly recovered as crossing the upper threshold and is found having a long face (Figure 10c). This character is ordered.

Character 45: Apertura narium externa

*Definition*—Length of emargination of the apertura narium externa (Figure 8b) relative to the prefrontal length (Figure 8b) (modified from Meylan, 1987, 41, 42): 0 = medial emargination present (Figure 8c); 1 = lateral emargination present, but shallow, 0–20% (Figure 10a); 2 = lateral emargination present, but deep, >20% (Figure 10b,c).

*Comment*—We herein unite and modify the original characters 41 and 42 of Meylan (1987). These morphometric characters utilize the length of the medial process of the prefrontal within the narial emargination over the total length of the prefrontal in dorsal view (Figure 8b). Additionally, we use state 0 as the medial emargination of the apertura narium externa, which among extant trionychids is autapomorphic of *Cyclanorbis elegans* (Figures 7c and 8c). Polymorphism is scored implementing our standard scoring protocol (see Section 2). The strongest narial emargination is found in *Apalone* spp. and *Palea steindachneri* (Figures 7a, 8b, and 10d). Polymorphism is reported for *Chitra chitra*, *Dogania subplana*, *Pelodiscus* sp., *Trionyx triunguis* and *Rafetus swinhoei*. All other species have a shallow emargination (Figures 7b,d and 10a,d). This character is ordered.

Character 46: Frontal 1

*Definition*—Anteroposterior length of frontal contact (Figure 8c) relative to anteroposterior length of frontals in dorsal view (Figure 8c) (new character): 0 = <40% (Figure 8c); 1 = 40–80% (Figure 10a); 2 = >80% (Figure 10d).

*Comment*—This character aims to quantify the intrusion of the posterior process of the prefrontal into the midline contact of the frontals. This character is quantified by measuring the anteroposterior length of the midline frontal contact in dorsal view on the skull surface over the total anteroposterior length of the frontal in dorsal view (Figure 8c). Polymorphism is scored implementing our standard scoring protocol (see Section 2). *Cyclanorbis elegans* has the shortest midline contact of the frontals among trionychids (Figure 10e). Indeed, in some specimens of this species, the frontals are missing a midline contact entirely, but we do not create a separate character state, as it only occurs polymorphically at a low level. On the other hand, suction feeders with long and flat skulls, such as *Chitra* spp., *Cycloderma frenatum*, and *Pelochelys* spp., tend to have a long midline contact of the frontals (Figure 10d). This character forms a morphocline that can be ordered.

Character 47: Frontal 2

*Definition*—Midline fusion of the frontals (modified from Gaffney, 1977, 6): 0 = absent; 1 = partially to fully present.

*Comment*—This character pertains to the partial or total fusion of the frontals (see Evers et al., 2023, fig. 6). Among fossil trionychids, surficial or complete fusion had previously been described for the fossils *Gilmoremys lancensis* (see Joyce & Lyson, 2011) and *Plastomenus thomasii* (see Evers et al., 2023). No extant species of trionychid shows a total fusion of the frontals, but a partial midline fusion is polymorphically found in some specimens of *Chitra indica* and *Pelodiscus bibroni*, and fusion of the frontals seems additionally common in *Apalone ferox*. This character is ordered.

Character 48: Postorbital 1

*Definition*—Postorbital (Evers et al., 2023, 97): 0 = absent; 1 = present.

*Comment*—This character scores the presence or absence of a postorbital (see Evers et al., 2023, fig. 3). This character has so far only been reported for a single specimen of *Plastomenus thomasii* (Evers et al., 2023). No other trionychid, extinct or extant, has been reported to lack the postorbital to date.

Character 49: Postorbital bar 1

*Definition*—Anteroposterior length of the postorbital bar (Figure 8c) relative to anteroposterior length of the orbit in dorsal view (Figure 8c) (modified from Meylan, 1987, 75): 0 = <20% (Figure 10b); 1 = 20–50% (Figure 10a); 2 = 50–100% (Figure 10e); 3 = 100–200% (Figure 10d); 4 = >200% (Figure 9c).

*Comment*—We herein expand the character from Meylan (1987) to include additional character states. The postorbital bar extends from the posterior edge of the orbit to the anterior limit of the upper temporal fossa and is formed by the jugal, the parietal and, when present, the postorbital. The relative length of the postorbital bar is measured by comparing the anteroposterior length of the postorbital bar from the posterior limit of the orbit to the anterior edge of the upper temporal emargination relative to the anteroposterior length of the orbit as observed in dorsal view (Figure 8c). In the available sample, *Apalone mutica* and *Dogania subplana* have the shortest postorbital bar (Figure 10b) while *Chitra* spp., *Cycloderma* spp., and *Pelochelys* spp. have the longest (Figures 9b,c and 10d). Indeed, *Chitra* spp. shows an extreme morphology where the postorbital bar is twice the length of the orbit. All other taxa fill the range in between. This character forms a morphocline that can be ordered.

Character 50: Postorbital bar 2

*Definition*—Contact between the jugal and parietal (modified from Meylan, 1987, 34, 35): 0 = absent, postorbital contributes to the margin of the upper temporal emargination; 1 = present within the fossa temporalis, postorbital contributes to the margin of the upper temporal emargination (Figure 10a); 2 = present on the surface of the skull, postorbital is excluded from the margin of the upper temporal emargination (Figure 10d).

*Comment*—We fuse two characters from Meylan (1987) that independently score for the absence or presence of a jugal‐parietal contact on the skull surface (34) and within the temporal fossa (35). These characters are interdependent, as our observations indicate that a contact on the skull surface does not occur in trionychids without an initial contact within the fossa. Meylan (1987) scored all trionychids as exhibiting contact of the jugal with the parietal within the temporal fossa, but we found a number of exceptions. We therefore fused Meylan's (1987) two characters into a single character with two derived character states that form a morphocline. A jugal‐parietal contact is lacking in the extant trionychid *Trionyx triunguis* and the fossil plastomenid *Hutchemys rememdium* (Girard et al., 2024). Otherwise, a contact between the jugal and parietal occurs in the fossa temporalis in *Amyda* spp., *Apalone spinifera*, *Nilssonia gangetica* and *Rafetus* spp. (Figures 8b and 10a,b). In most cyclanorbines, *Apalone mutica*, *Chitra* spp., and *Pelochelys cantorii*, the jugal contacts the parietal on the skull surface and excludes the postorbital from the upper temporal emargination (Figures 8c and 10c–e). The remaining species of trionychids exhibit variation at a frequency above 20% and are therefore scored polymorphic. This character is ordered.

Character 51: Otic Capsule

*Definition*—Relative contribution of the parietal to the anterior margin of the otic capsule (Figure 8c) (modified from Meylan, 1987, 78): 0 = <20% (Figure 10d); 1 = 20–40% (Figure 10e); 2 = >40% (Figure 10c).

*Comment*—Meylan (1987) developed a character that addresses variation in the contribution of the parietal to the processus trochlearis oticum using cutoffs of less than 15.6% versus more than 22.1%. As these character states were optimized for his sample, we modified this character by evenly subdividing the observed range into three character states with rounded thresholds. The processus trochlearis typically shows a different texture than the surrounding bones, but it is difficult to define its extent precisely. We, therefore, herein use the anterior margin of the otic capsule, which we medially defined by the lateral swelling of the parietal and laterally defined at the base of the temporal bar (Figure 8c). All measurements were obtained over the curve using ImageJ. Specimens approaching a threshold within 2 percentage points are scored polymorphic. Species are only scored polymorphic if more than 20% of individuals exhibit a given character state. Polymorphic individuals spanning a given threshold are hereby referred to as the majority state. Extreme outliers are disregarded. The contribution of the parietal to the anterior margin of the otic capsule is the smallest in the chitrines *Chitra* spp. and *Pelochelys* spp. (Figure 10d), the amydines *Nilssonia hurum*, *Nilssonia leithii*, and *Pelodiscus* sp., and the cyclanorbines *Cyclanorbis senegalensis* and *Lissemys punctata* (Figure 10a). The species with the highest contribution of the parietal to the anterior margin of the otic capsule are the apalonines *Apalone mutica* and *Apalone spinifera*. The remaining sample displays the intermediate condition for this character. This character is ordered.

Character 52: Parietal 1

*Definition*—Relationship of dorsal plate of the parietal relative to its vertical plate (new character): 0 = dorsal and vertical plate form a confluent, domed surface (Figure 10a); 1 = step‐like boundary separates dorsal plate from vertical plate (Figure 10b); 2 = dorsal plate partially roofs the vertical plate (Figure 10d).

*Comment*—This character attempts to capture three distinct morphotypes apparent to the development of the dorsal and vertical plates of the parietal in trionychids as either jointly forming a domed surface that accommodates for the brain (Figure 10a), an angled surface visually separating the dorsal skull roof from the upper temporal fossa (Figure 10b), or a broadened dorsal shelf that overhangs the upper temporal fossa (Figure 10d). We note in our sample a strong ontogenetic trend for this character in that more juvenile specimens tend to exhibit lower character states and more skeletally mature specimens higher character states. We therefore exceptionally reduce the polymorphism apparent to the primary dataset by scoring species by reference to the most skeletally mature individuals. *Lissemys punctata* and *Cyclanorbis senegalensis* are the only species that exhibit a domed braincase as adults (Figure 10a). Apalonines, amydines, *Cyclanorbis elegans*, and *Trionyx triunguis* exhibit as adults a step‐like boundary between the dorsal surface and the vertical process of the parietal (Figures 8, 9, and 10c,e). The dorsal plate of the parietal overhangs the temporal fossa in the long‐skulled species *Chitra* spp., *Cycloderma* spp., and *Pelochelys* spp. (Figures 9b,c and 10d). *Lissemys ceylonensis* variably shows states 1 and 2. This character is ordered.

Character 53: Parietal 2

*Definition*—Lateral expansion of the vertical plate of the parietal covering the triple junction formed between the opisthotic, prootic, and supraoccipital (new character): 0 = absent (Figure 10b); 1 = present (Figure 10a).

*Comment*—In some species of trionychids, a lateral, sheet‐like expansion of the vertical plate of the parietal dorsally covers the triple junction formed by the opisthotic, prootic, and supraoccipital (Figure 10a). This condition is found consistently in *Cyclanorbis* spp. and *Lissemys punctata*, and occurs polymorphically in *Cycloderma frenatum* and *Lissemys punctata* (Figures 8c and 10a), but is absent in all trionychines.

Character 54: Quadrate 1

*Definition*—Dorsal exposure of the quadrate within the upper temporal fossa (new character): 0 = broad (Figure 10d); 1 = notably narrow (Figure 10a).

*Comment*—This character addresses variation to the degree of the exposure of the quadrate in the upper temporal fossa. In some species, the quadrate is only exposed as a narrow band of bone, the remainder being overlapped by the squamosal. This condition is consistently observed in *Apalone mutica*, *Apalone spinifera*, our single specimen of *Lissemys scutata*, *Palea steindachneri*, and *Rafetus euphraticus* (Figure 8b) and found polymorphically (i.e., at a frequency between 20 and 80%) in *Apalone ferox*, *Cycloderma frenatum*, *Dogania subplana*, *Lissemys punctata*, *Nilssonia gangetica*, *Nilssonia hurum*, *Pelochelys cantorii*, *Pelodiscus* sp., *Rafetus swinhoei*, and *Trionyx triunguis* (Figure 10a,b). The dorsal exposure of the quadrate is consistently broad in all remaining species (Figures 8c and 10d).

Character 55: Cavum tympani

*Definition*—Bony ridge connecting the incisura columellae auris to the posteroventral margin of the cavum tympani (new character): 0 = absent (Figure 9c); 1 = present, but minor (Figure 9b); 2 = present and distinct (Figure 9a).

*Comment*—A posteroventrally oriented ridge extending within the cavum tympani from the fully enclosed incisura columellae auris to the margin of the cavum tympani is variably developed among trionychids (Figure 9). This ridge is consistently absent in all chitrines (Figure 9c) and *Apalone mutica* and polymorphically absent in *Rafetus swinhoei* (4/5 of our sample). The degree of expression of this ridge is variable in species where it is present, but it seems to be distinct in cyclanorbines and minor in trionychines (Figure 9). This character forms a morphocline that can be ordered.

Character 56: Fenestra postotica 1

**FIGURE 11:**
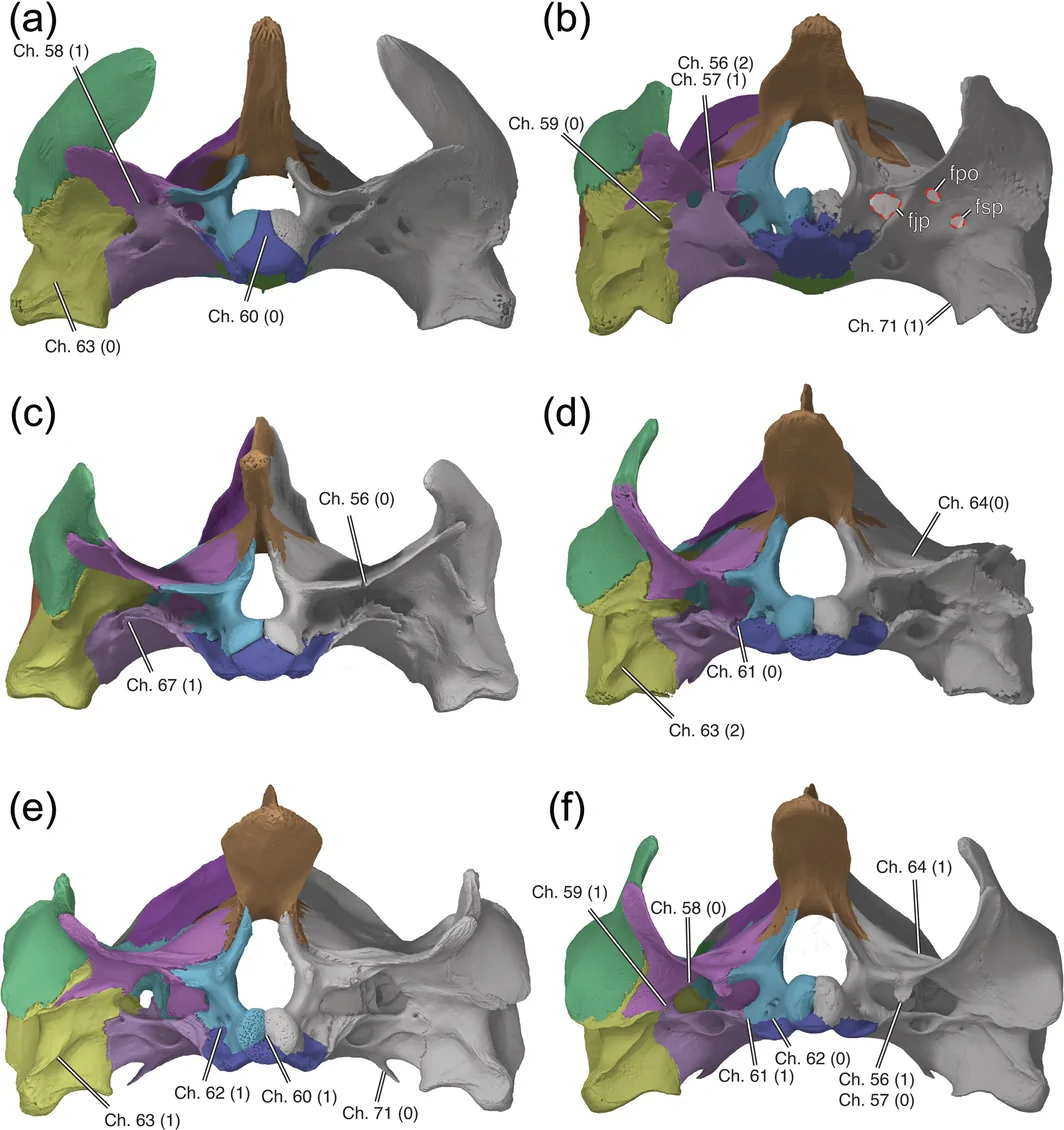
3D models of crania of select trionychid species in posterior view illustrating character states. (a) *Cycloderma aubryi* NMB 18041, (b) *Lissemys punctata* SMF 74141, (c) *Chitra chitra* PCHP 4995, (d) *Trionyx triunguis* PCHP 4559, (e) *Rafetus euphraticus* NHMW 132, (f) *Amyda cartilaginea* FMNH 224117. Images are not to scale. *fjp* foramen jugulare posterius, *fpo* fenestra postotica, *fsp* foramen stapedialis posterius.

*Definition*—Separation of the foramen jugulare posterius from the remainder of the fenestra postotica (modified from Sterli et al., 2013, 91): 0 = fully absent or only hinted at (Figure 11c); 1 = strongly hinted at by a dorsal process of the pterygoid and/or a ventral process of opisthotic/exoccipital (Figure 11f); 2 = fully separated (Figure 11b).

*Comment*—Meylan (1987) developed two characters that clarify which bones separate the foramen jugulare posterius from the fenestra postotica, while neglecting to formulate a character that unites all taxa with formed foramina jugulare posterius. We, therefore, expanded the character of Sterli et al. (2013, 91) to pertain to the full, partial, or lacking formation of this foramen (Figure 11) and address the bones that contribute to this foramen in separate characters (see Character 57 below). Polymorphism is scored implementing our standard protocols (see Section 2). While the foramen jugulare posterius is fully separated from the fenestra postotica in all cyclanorbines (Figure 11a,b), the separation of the foramen from the fenestra is only hinted at in most amydines (Figure 11f) and mostly absent in apalonines and chitrines (Figure 11c–e). This character is ordered.

Character 57: Fenestra postotica 2

*Definition*—Bones involved in the separation of the foramen jugulare posterius from the fenestra postotica (modified from Meylan, 1987, 58, 59): 0 = partial or full separation of foramen jugulare posterius undertaken from above (i.e., exoccipital and/or opisthotic) (Figure 11f); 1 = partial or full separation of foramen jugulare posterius undertaken from below (i.e., pterygoid) (Figure 11b).

*Comment*—Following up from the previous character, we combine two characters from Meylan (1987) to address which bones are involved in the separation of the foramen jugulare posterius from the fenestra postotica. Specimens lacking a fully separated foramen jugulare posterius are scored inapplicable. Polymorphism is scored implementing our standard scoring protocol (see Section 2). In our sample, the foramen jugulare posterius is separated from below in cyclanorbines (Figure 11a,b), from above in amydines and apalonines (Figure 11e,f), but variably among chitrines.

Character 58: Fenestra postotica 3

*Definition*—Separation of the foramen stapedialis posterius from the remainder of the fenestra postotica (new character): 0 = absent or hinted at (Figure 11f); 1 = fully separated (Figure 11a) (new character).

*Comment*—The foramen stapedialis posterius is a distinct foramen through which the arteria stapedialis enters the cavum acustico‐jugulare (Albrecht, 1967). Usually, this opening is confluent with the fenestra postotica and therefore indistinct from it, but a separate foramen is hinted at or fully developed in some taxa (Figure 11). Polymorphism is scored implementing our standard scoring protocol (see Section 2). *Cycloderma aubryi* is the only species to consistently have the foramen stapedialis posterius separated from the fenestra postotica (Figure 11a). This condition seems otherwise common in *Lissemys* spp. (Figures 1e and 11b) and occasionally found in C*hitra indica*. The separation of the foramen from the fenestra is otherwise hinted at in all cyclanorbines and *Nilssonia gangetica*, *Nilssonia leithii*, *Pelochelys cantorii*, and *Rafetus euphraticus*, and observed in some specimens of *Chitra chitra*, *Trionyx triunguis*, and *Rafetus swinhoei*. For all other species, the foramen stapedialis posterius is confluent with the fenestra postotica.

Character 59: Fenestra postotica 4

*Definition*—Pterygoid‐opisthotic contact lateral to the fenestra postotica (modified from Meylan, 1987, 58, 59): 0 = absent (Figure 11b); 1 = present, quadrate excluded from fenestra postotica or any of its subparts (Figure 11f).

*Comment*—The fenestra postotica of trionychids can be subdivided into three separate openings: the medial foramen jugulare posterius, the lateral foramen stapedialis posterius, and the residual fenestra postotica (Figures 1e and 11a,b). The pterygoid‐opisthotic contact that is the focus of this character does not produce one of the above‐listed subdivisions of the fenestra postotica, but rather is located lateral to the foramen stapedialis posterius portion of the fenestra postotica. The quadrate is excluded from the fenestra postotica complex by a pterygoid‐opisthotic contact in *Apalone ferox*, *Nilssonia gangetica*, and *Nilssonia nigricans* (Figure 11f). It contributes polymorphically to this opening in *Amyda* spp., *Apalone mutica*, *Apalone spinifera*, *Cyclanorbis senegalensis*, *Nilssonia formosa*, *Nilssonia hurum*, and *Pelodiscus* sp. The quadrate contributes to the fenestra postotica complex in all remaining species (Figure 11a–e).

Character 60: Exoccipital 1

*Definition*—Medial contact of the exoccipitals in the floor of the foramen magnum (Evers & Benson, 2019, 114): 0 = absent (Figure 11a); 1 = present (Figure 11e).

*Comment*—This character pertains to the absence or presence of a medial contact of the exoccipital with its counterpart in the floor of the foramen magnum, which correlates in our sample with a contact of these bones within the occipital condyle (Figure 11). Polymorphism is scored implementing our standard scoring protocol (see Section 2). Most trionychids have a medial contact of the exoccipital (Figure 11b–f). *Cycloderma aubryi* is the only species consistently lacking this contact (Figure 11a). It is polymorphically missing in *Apalone spinifera*, *Chitra* spp., and *Pelochelys cantorii*.

Character 61: Exoccipital 2

*Definition*—Contact of the exoccipital with the pterygoid (Meylan, 1987): 0 = absent (Figure 11d); 1 = present (Figure 11f).

*Comment*—This character, which pertains to a contact of the exoccipital with the pterygoid in the floor of the fenestra postotica, remains unchanged from its original definition by Meylan (1987). The basioccipital contributes to the fenestra postotica when this contact is lacking (Figure 11d). Meylan (1987) found this character to be an autapomorphy of *Trionyx triunguis*. This conclusion is supported by our sample (Figure 11d). We find no polymorphism in the scoring of this character.

Character 62: Foramina nervi hypoglossi

*Definition*—Participation of the basioccipital in the formation of the foramina nervi hypoglossi (new character): 0 = absent (Figure 11f); 1 = present (Figure 11e).

*Comment*—The foramina nervi hypoglossi, a series of three small foramina positioned ventrolaterally to the foramen magnus (Figure 1e), are variably either formed by the exoccipital alone (Figure 11f) or jointly by the exoccipital and basioccipital (Figure 11e). *Lissemys scutata* and *Pelodiscus* sp. are the only species that consistently lack a participation of the basioccipital in the formation of the foramina hypoglossi (Figure 11a,f). This condition is variably present in *Amyda* spp., *Apalone mutica*, *Cycloderma* spp., *Cyclanorbis senegalensis*, *Dogania subplana*, *Lissemys punctata*, and *Palea steindachneri*. The basioccipital consistently participates in the formation of the foramina hypoglossi in the remaining trionychids (Figure 11b–e).

Character 63: Infolding ridge of the quadrate

*Definition*—Infolding ridge on the quadrate posterodorsal to the mandibular condyles (modified from Anquetin et al., 2015, 37): 0 = absent or extremely minor (Figure 11a); 1 = present but low (Figure 11e); 2 = present and forming a massive, overhanging flange (Figure 11d).

*Comment*—This character documents the variable presence and expression of the infolding ridge of the quadrate, which, when present, is a diagonally oriented elevation located on the posterior side of the mandibular condyles (Figures 1e and 11). While the presence of this ridge can be established easily from photographs, assessing how distinctly it is developed demands manipulating specimens manually or virtually. The infolding ridge is absent in *Cycloderma* spp. and *Pelochelys* spp. (Figure 11a). The ridge is present, but low in cyclanorbines, several amydines, and *Rafetus* spp. (Figure 11b,e,f). It is well developed in *Apalone mutica*, *Chitra* spp., and *Nilssonia gangetica*. Polymorphism for state 0 and 1 is found in *Dogania subplana*, *Lissemys ceylonensis*, *Nilssonia formosa*, *Nilssonia hurum*, and *Nilssonia nigricans*. Polymorphism for state 1 and 2 is found in *Apalone ferox*, *Apalone spinifera*, *Palea steindachneri*, and *Trionyx triunguis*. This character forms a morphocline.

Character 64: Opisthotic

*Definition*—Ridge on the dorsoposterior part of the opisthotic (new character): 0 = absent (Figure 11d); 1 = present (Figure 11f).

*Comment*—In some species of trionychids, a ridge is developed within the upper temporal fossa on the posterodorsal surface of the opisthotic (Figure 11f). This ridge is present in *Cyclanorbis elegans*, *Amyda* spp., *Nilssonia gangetica*, *Nilssonia leithii*, and *Rafetus* spp. and developed polymorphically in *Apalone mutica*, *Apalone spinifera*, *Cyclanorbis senegalensis*, *Dogania subplana*, *Lissemys punctata*, *Nilssonia formosa*, *Nilssonia hurum*, *Nilssonia nigricans*, and *Pelodiscus* sp. The ridge is absent in *Apalone ferox*, all chitrines, *Cycloderma* spp., *Lissemys ceylonensis*, *Lissemys scutata*, and *Palea steindachneri*.

Character 65: Parabasisphenoid 1

**FIGURE 12:**
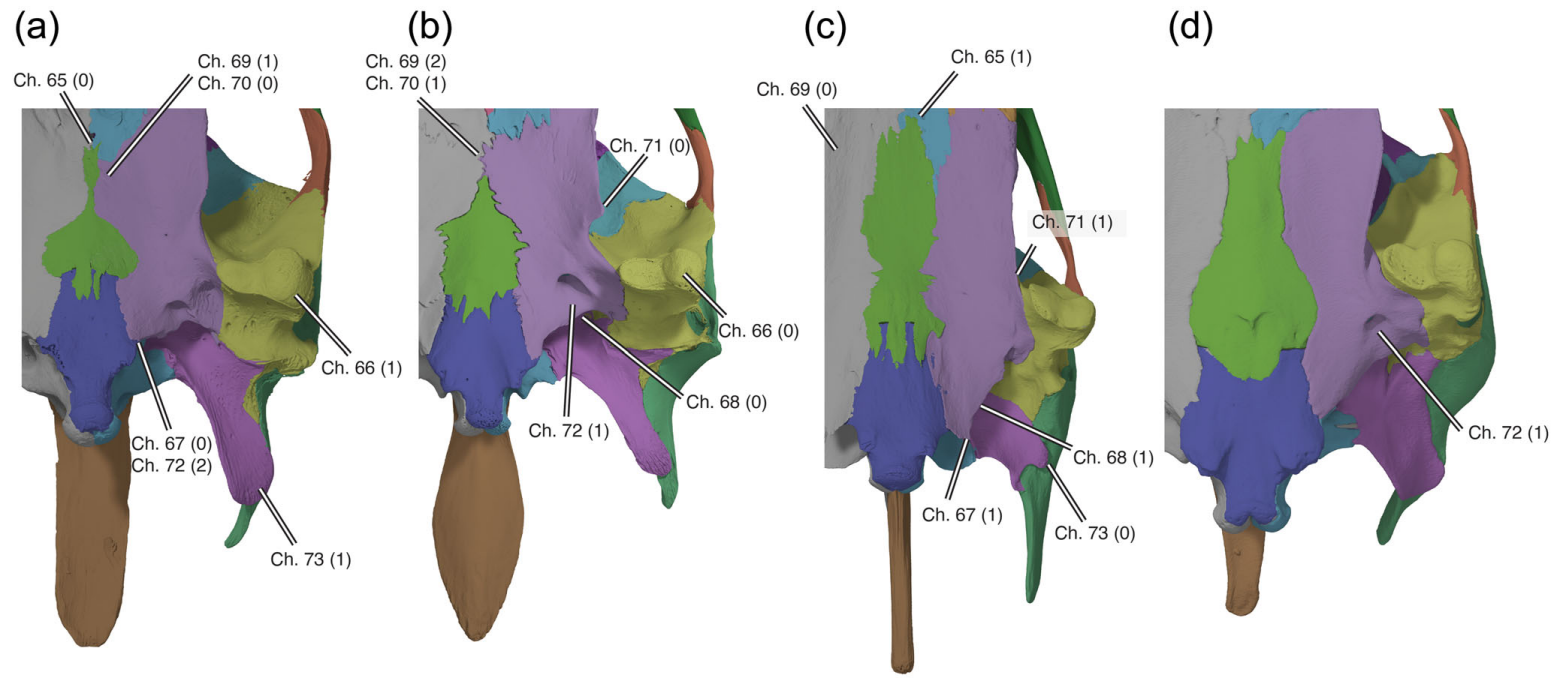
3D models of the cranium of select trionychid species in ventral view illustrating character states with a focus on the basicranium. (a) *Cyclanorbis elegans* NHMW 157, (b) *Rafetus euphraticus* NHMW 132, (c) *Chitra chitra* PCHP 4995, (d) *Apalone mutica* PCHP 2655. Images are not to scale.

*Definition*—Elongate anterior protrusion of the parabasisphenoid between the palatines (new character): 0 = absent (Figure 12d); 1 = present (Figure 12c).

*Comment*—This character describes the presence or absence of a deep anterior protrusion of the parabasisphenoid between the pair of palatines (Figure 12c). A distinct anterior protrusion of the parabasisphenoid is consistently found in *Chitra* spp. and *Pelochelys* spp. (Figures 4b and 12c) but polymorphically developed in *Amyda* spp., *Cycloderma* spp., *Cyclanorbis senegalensis*, *Dogania subplana*, *Nilssonia hurum*, *Palea steindachneri*, and *Rafetus swinhoei*. It is consistently absent in the remainder of the sample.

Character 66: Mandibular condyles 1

*Definition*—Relative size of the mandibular condyles (new character): 0 = both condyles similar in size (Figure 12b); 1 = medial condyle much smaller than lateral condyle (Figure 12a).

*Comment*—The facet of the quadrate for articulation with the mandible is either formed by equally sized medial and lateral condyles (Figures 5c and 12b–d) or by differently sized condyles, of which the medial one is significantly smaller than the lateral one (Figures 5d and 12a). The condyles are consistently of similar size in *Chitra* spp., *Apalone mutica*, *Amyda ornata*, *Cycloderma aubryi*, and *Pelochelys* spp. and consistently of different sizes in *Amyda cartilaginea*, *Apalone ferox*, *Cyclanorbis* spp., *Dogania subplana*, *Lissemys* spp., *Nilssonia hurum*, and *Rafetus swinhoei*. All other species show polymorphism.

Character 67: Pterygoid 1

*Definition*—Secondary occipital tubercles formed by pterygoid (new character): 0 = absent (Figure 12a); 1 = present (Figure 12c).

*Comment*—This character describes the presence of a posterior process formed by a ridge of the pterygoid posteriorly extending past the fenestra postotica and forming tubercles lateral to the basioccipital tubercles (Figures 11c and 12c). This condition is observed in *Chitra* spp., *Cycloderma* spp., *Lissemys* spp., and *Pelodiscus bibroni*.

Character 68: Pterygoid 2

*Definition*—Ventral flooring of the foramen posterius canalis carotici interni by accessory ridge of the pterygoid (modified from Meylan, 1987, 60): 0 = absent (Figure 12b); 1 = present (Figure 12c).

*Comment*—Meylan (1987) developed a character pertaining to the position of the foramen posterius canalis carotici interni (fpcci) relative to the lateral crest of the basioccipital tubercle. We note that a number of crests are developed by the pterygoid in this part of the skull, but their homology is not fully apparent to us. Two morphotypes are nonetheless apparent, one in which the fpcci is roofed by a crest of the pterygoid (Figure 12b), another where the fpcci is partially to fully floored by what looks to be an accessory crest of the pterygoid (Figure 12c). In our sample, the latter state is only present in *Chitra* spp. and *Pelochelys cantorii* (Figures 11c and 12c).

Character 69: Pterygoid 3

*Definition*—Presence of a medial underlap of the parabasisphenoid by the palatine and/or pterygoid (modified from Meylan, 1987, 46, 64): 0 = absent or minor (Figure 12c); 1 = present and distinct, but midline contact of pterygoids absent (Figure 12a); 2 = present and distinct, midline contact of pterygoids present (Figure 12b).

*Comment*—Meylan (1987) developed two characters that pertain to the size and shape of the ventral exposure of the parabasisphenoid. The first character addresses the shape of the parabasisphenoid as either being not medially constricted, occasionally medially constricted, or medially constricted. The second pertains to a contact of the parabasisphenoid with the palatines, which he concluded to be an autapomorphy of *Rafetus euphraticus*. We modified this character as pertaining to the ventromedial covering of the parabasisphenoid by the pterygoid as this is what controls the ventral exposure of the parabasisphenoid and its contact with the palatines. Incidentally, the medial contact of the pterygoid is superficial in nature as the palatine and parabasisphenoid are always in contact with one another (Figures 2e and 4). *Rafetus euphraticus* is the only species in which a midline contact of the pterygoid is present in about 90% of the sample. We otherwise score this contact as polymorphic for *Apalone spinifera*, *Cyclanorbis elegans*, and *Nilssonia leithii* (Figures 2b,e and 12b). A distinct underlap of the parabasisphenoid by the pterygoid is present in *Chitra vandijki*, *Cyclanorbis senegalensis*, *Nilssonia formosa*, and *Palea steindachneri*, and polymorphically present in *Cycloderma frenatum*, *Dogania subplana*, *Lissemys punctata*, *Nilssonia hurum*, *Nilssonia nigricans*, and *Pelodiscus* sp. (Figure 12a). Finally, all other species show no underlap of the parabasisphenoid (Figure 12c). *Nilssonia gangetica* shows all three character states. This character forms a morphocline that can be ordered.

Character 70: Pterygoid 4

*Definition*—Position of the medial process of the pterygoid underlapping the parabasisphenoid (new character): 0 = medial process located midway along the length of the parabasisphenoid, ventral parabasisphenoid exposure appears medially constricted or separated into two fields (Figure 12a); 1 = medial process located further anteriorly, ventral parabasisphenoid exposure elongate, anterior contact of the parabasisphenoid with palatines and/or vomer is reduced or absent (Figure 12b).

*Comment*—This character is a direct follow‐up to the previous character (see Character 69 above) and documents variation in the position of the underlap of the parabasisphenoid by the medial processes of the pterygoids. This character is scored inapplicable for species with no parabasisphenoid underlap (Character 69, state 0). The underlap is generally located anteriorly in cyclanorbines, except for *Lissemys ceylonensis*, in those apalonines for which this character is applicable, and *Nilssonia formosa*, *Nilssonia nigricans*, and *Trionyx triunguis* (Figure 12b). It is developed variably in *Amyda* spp. It is consistently located posteriorly in the remaining amydines, *Chitra vandijki*, and *Lissemys ceylonensis* (Figure 12a).

Character 71: Pterygoid 5

*Definition*—Posterior terminus of ventral pterygoid ridge (new character): 0 = ventral pterygoid ridge ends anterior to the condylus articularis creating a distinct lateral notch to the margin of the pterygoid in ventral view (Figures 11e and 12b); 1 = ventral pterygoid ridge is confluent with the condylus articularis, lateral pterygoid notch absent (Figures 11b and 12c).

*Comment*—This character documents the variable presence of a notch in the posterolateral margin of the pterygoid resulting from the termination of the ventral pterygoid ridge anterior to the condylus articularis. This ridge is associated with the M. adductor mandibulae internus Pars pterygoideus (Rollot et al., 2024; Werneburg, 2011). This character is easier to score in a ventroposterior view, as the notch is more visible (Figure 11). A ridge confluent with the articular condyle is notably found in *Chitra* spp., *Cycloderma* spp., *Cyclanorbis elegans*, *Lissemys ceylonensis*, and *Pelochelys cantorii* (Figures 11a–c and 12c). A notch is present in all other trionychids, but expressed most clearly in *Rafetus euphraticus* (Figures 11f and 12b).

Character 72: Canalis carotici interni 1

*Definition*—Position of the foramen posterius canalis carotici interni relative to the main contact between the parabasisphenoid and the basioccipital (new character): 0 = clearly anterior to contact (Figure 12d); 1 = more or less at contact (Figure 12b); 2 = clearly posterior to contact (Figure 12a).

*Comment*—This character documents variation in the position of the foramen posterius canalis carotici interni (FPCCI) relative to the main contact of the parabasisphenoid and basioccipital. As the parabasisphenoids of many taxa form highly irregular, sheet‐like processes that underlap the basioccipital (Figure 12), this character is phrased specifically to pertain to the position of the FPCCI relative to the blunt, deep contact of the parabasisphenoid with the basioccipital. This contact can often be observed in ventral view despite the sheet‐like processes (Figure 12a,c), but otherwise must be scored using CT scans. The foramen posterius canalis carotici interni is generally located posterior to the parabasisphenoid‐basioccipital contact in chitrines (Figure 12c), posterior to or at the contact in amydines and *Lissemys* spp. (Figure 12a), and variable in other groups (Figure 12b). The foramen is consistently placed anterior to the contact only in *Apalone mutica* and *Cyclanorbis senegalensis* (Figure 12d). This character is ordered.

Character 73: Opisthotic

*Definition*—Relative length of the processus paroccipitalis (new character): 0 = short, processus paroccipitalis protrudes less far posteriorly than the occipital condyle (Figure 12c); 1 = long, processus paroccipitalis protrudes further posteriorly than the occipital condyle (Figure 12a).

*Comment*—The processus paroccipitalis is the posteriorly oriented process of the opisthotic that partially underlaps the squamosal. This character documents the length of this process relative to the occipital condyle. In the long‐skulled species *Chitra* spp., *Cycloderma aubryi*, and *Pelochelys* spp., the processus paroccipitalis is short and the elongate squamosal horn remains free medially (Figure 12c). In all other species of trionychids, the processus paroccipitalis is long, protruding posteriorly far beyond the level of the occipital condyle, and the squamosal horn is supported medially by the processus paroccipitalis (Figure 12a,b).

Character 74: Abducens nerve

*Definition*—Enclosure of the canalis nervi abducentis (modified from Evers et al., 2023, 107): 0 = formed as a fully ossified canal (Figure 4a); 1 = formed as a tight, dorsally open groove (Figure 4c); 2 = no canal or groove apparent (Figure 4d).

*Comment*—This character was originally introduced by Evers et al. (2023) as they found the abducens nerve canals of the extinct trionychid *Plastomenus thomasii* to form dorsally open grooves rather than dorsally enclosed canals. We herein add a new character state for the condition where no canal or groove for the abducens nerve is apparent on the dorsal surface of the parabasisphenoid. Following our standard scoring procedure, right/left asymmetry is scored polymorphically and treated as two different data points in the final scoring of species. Our sample for this character is limited, as it can only be observed in 3D models obtained from CT scans. The most common condition for trionychines is a fully ossified canal (Figure 5a,b). The only exception is one specimen of *Chitra indica* (SMF 52769), which polymorphically exhibits a dorsally open groove on one side of the skull and a fully formed canal on the other (*Chitra indica*). Most of the variation for this character is found within *Cyclanorbinae*. While *Cycloderma frenatum* and *Lissemys punctata* have a fully formed canal, *Cycloderma aubryi* is found to have a dorsally open groove. Our specimen of *Cyclanorbis elegans* is asymmetric, having a dorsally open groove on one side and no groove or canal on the other. *Cyclanorbis senegalensis* is the only species consistently lacking a groove or canal. Finally, among our limited sample of two *Lissemys ceylonensis* specimens, one shows no apparent groove while the other is asymmetric with a canal on one side and a dorsally open groove on the other (Figure 5c). This character is ordered.

Character 75: Clinoid processes

*Definition*—Presence and expression of the clinoid processes (Figure 1b,f) (new character): 0 = absent (Figure 4d); 1 = pointed processes (Figure 4b), 2 = spatulate processes (Figure 4e).

*Comment*—The clinoid processes of the parabasisphenoid project anteriorly from the lateral edge of the dorsum sellae to overhang the anterior opening of the canalis nervi abducentis (Figure 1b,f). We distinguish between clinoid processes that are pointed (Figure 4b) and spatulate (Figure 4e). In our sample, observation was restricted to specimens documented by CT scans. Clinoid processes are generally absent in cyclanorbines, only being asymmetrically present in a single specimen of *Lissemys ceylonensis*. They are generally present in trionychines, though polymorphically absent in *Amyda cartilaginea*, *Apalone ferox*, *Dogania subplana*, and *Pelodiscus* sp. Among trionychines, clinoid processes are most commonly developed as pointed projections (Figure 5a), but the spatulate condition is consistently found in *Apalone spinifera*, *Nilssonia formosa*, *Pelochelys cantorii*, and variably in some specimens of *Apalone ferox*, *Chitra chitra*, and *Trionyx triunguis* (Figure 5b). The presence and development of the clinoid processes plausibly depend on ontogeny, but our sample size for this character is not sufficient to establish a correlation. This character is ordered.

Character 76: Foramen jugulare anterius

*Definition*—Contact of the basioccipital with the opisthotic anterior to the foramen jugulare anterius (new character): 0 = absent (Figure 5a); 1 = present (Figure 5b).

*Comment*—This character aims to document a contact between the basioccipital and opisthotic anteroventral to the foramen jugulare anterius. The contact is present in all cyclanorbines. It is absent in our sample of *Apalone mutica*, *Apalone spinifera*, *Pelochelys cantorii*, *Pelodiscus* sp., and *Rafetus euphraticus*, and polymorphic in *Apalone ferox*, *Chitra chitra*, *Palea steindachneri*, and *Trionyx triunguis*. In the remaining species of trionychines the contact is present.

Character 77: Canalis carotici interni 2

*Definition*—Dorsal fenestra in the canalis carotici interni (new character): 0 = absent, the cci is fully enclosed dorsally; 1 = present, but minor (Figure 4c); 2 = present and large (Figure 4a).

*Comment*—Observation of the internal features of the skull of trionychids through CT data shows that most trionychids have a large dorsal opening of the canalis carotici interni in the cavum labyrinthicum (Figure 5). The canalis carotici interni is fully enclosed by the pterygoid in *Cyclanorbis elegans* and some specimens of *Dogania subplana*. In most other trionychids, this opening is a large fenestra (Figure 5a,b). The exception, in which the fenestra is a minor opening, is found in *Cyclanorbis senegalensis*, *Lissemys punctata*, and *Nilssonia formosa* as well as some specimens of *Lissemys ceylonensis* and *Palea steindachneri* (Figure 5c). *Dogania subplana* shows all three character states. This character is ordered.

Character 78: Symphysis 1

**FIGURE 13:**
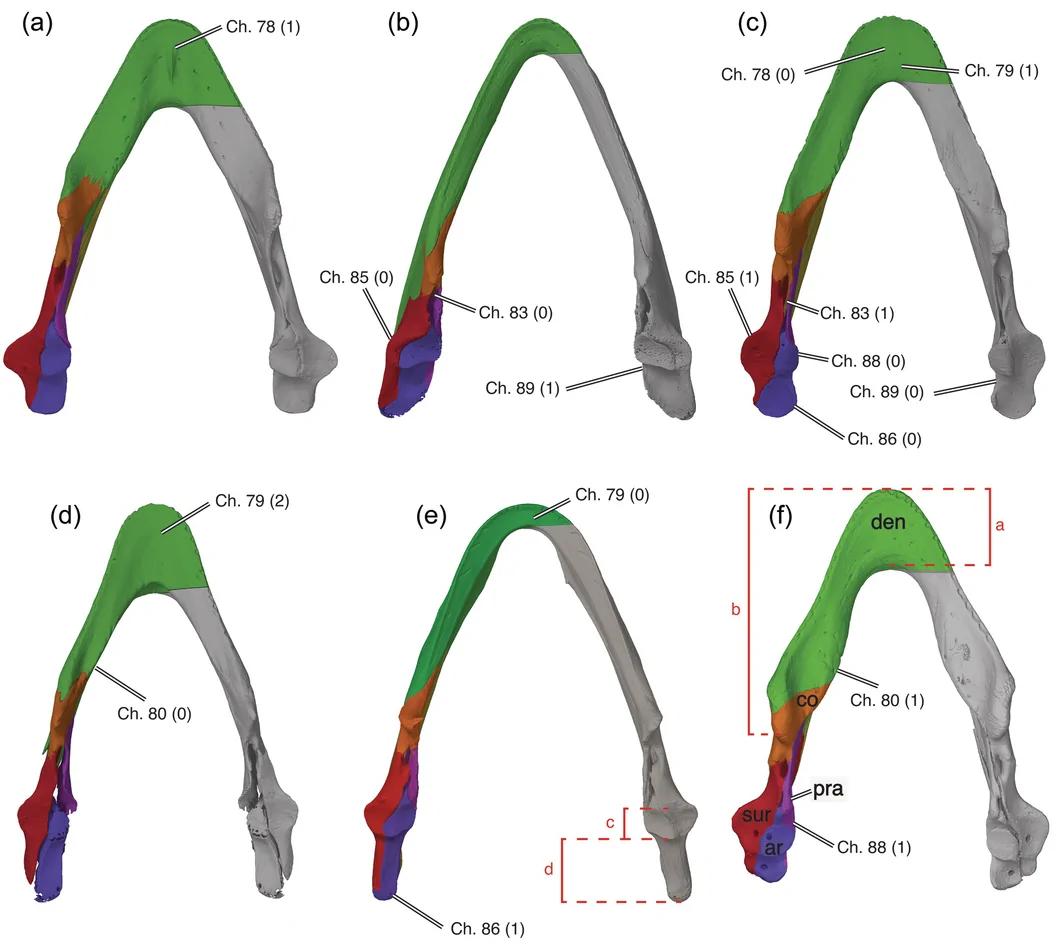
3D models of mandibles of select trionychid species in dorsal view illustrating measurements used herein. (a) *Amyda cartilaginea* FMNH 224117, (b) *Cycloderma aubryi* NMB 18041, (c) *Apalone spinifera* TMM 3817, (d) *Trionyx triunguis* PCHP 4559, (e) *Pelochelys cantorii* PCHP 4974, (f) *Lissemys ceylonensis* NKMB 2398. *ar* articular; *co* coronoid; *den* dentary; *pra* prearticular; *sur* surangular. *a* anteroposterior length of the symphysis; *b* anteroposterior length of the triturating surface; *c* anteroposterior length of area articularis; *d* anteroposterior length of the retroarticular process. Images are not to scale.

*Definition*—Symphyseal ridge (Meylan, 1987, 95): 0 = absent (Figure 13c); 1 = present (Figure 13a).

**FIGURE 14:**
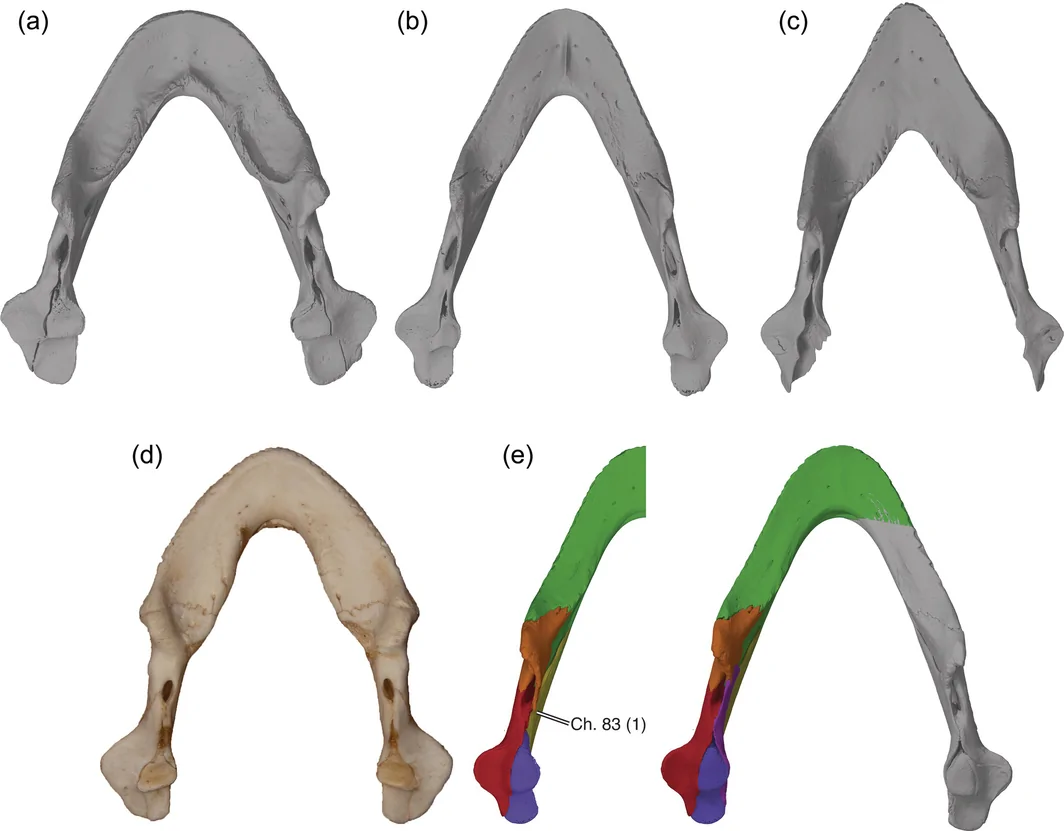
3D models (a–c, e) and photograph (d) of mandibles of select trionychid species in dorsal view. (a) *Nilssonia gangetica* SMF 52770, (b) *Nilssonia nigricans* NHMW 39090, (c) *Nilssonia leithii* NHMW 38299, (d) *Cyclanorbis elegans* PCHP 8215, (e) *Rafetus euphraticus* NHMW 132. Images are not to scale.

*Comment*—We continue using a character from Meylan (1987) that addresses the variable presence of a distinct median symphyseal ridge on the dorsal side of the dentary (Figure 13). Interestingly, even though we do not modify this character, we find a distribution different from that reported by Meylan (1987). In particular, we find a symphyseal ridge to be present in *Amyda* spp., *Nilssonia formosa*, *Nilssonia nigricans*, and polymorphically in *Nilssonia hurum* while Meylan (1987) additionally found it in all other *Nilssonia* species and *Dogania subplana*. We informally observe a correlation between the presence of a ridge and the specimen's size and therefore score species by reference to the most adult specimens. In *Nilssonia gangetica*, a short, triangular bony process is present on the posterior edge of the symphysis and extending in the midline of the triturating surface (Figure 14a). However, this condition is different from the symphyseal ridge found in the other amydines and is therefore scored absent.

Character 79: Symphysis 2

*Definition*—Anteroposterior length of the symphysis relative to the anteroposterior length of the triturating surface (Figure 13f) (modified from Hirayama, 1998, 39): 0 = <20% (Figure 13e); 1 = 20–40% (Figure 13c); 2 = >40% (Figure 13d).

*Comment*—The relative length of the mandibular symphysis varies greatly among trionychid species, including the fossil record, which documents trionychids with spatulate mandibles (Evers et al., 2023; Joyce & Lyson, 2011). We introduce a morphometric character that quantifies the anteroposterior length of the mandibular symphysis relative to the triturating surface of the mandible in dorsal view (Figure 13f). The triturating surface length is taken from the tip of the mandible to the coronoid process. Specimens are scored polymorphic if the measurement approaches a threshold within 2 percentage points. This character shows surprisingly little polymorphism. Its primary driver thus seems to be feeding mechanism and phylogeny, rather than ontogeny. The shortest mandibular symphyses are found among the suction‐feeders *Chitra* spp., *Cycloderma aubryi*, and *Pelochelys* spp. (Figure 13b,e) while the longest symphyses are found in *Apalone mutica*, *Cyclanorbis elegans*, *Nilssonia* spp., *Palea steindachneri*, *Pelodiscus* sp., and *Trionyx triunguis*. This character is ordered.

Character 80: Posteromedial flanges of the triturating surfaces

*Definition*—Posteromedial flanges of the triturating surfaces (Evers et al., 2022, 8): 0 = absent or indistinct (Figure 13d); 1 = present (Figure 13f).

*Comment*—We apply a character introduced by Evers et al. (2022), which pertains to a lingual expansion of the dentary. This expansion is located in the posterior part of the triturating surface, is medially oriented, and is formed by the dentary, often with a contribution from the coronoid (Figures 13 and 14). In trionychines, these expansions are exclusively found among amydines and clearly correlated with ontogeny in that they are mostly found in large adult specimens (see Section 3). The posteromedial flanges of the triturating surface are also found in *Cyclanorbis* spp. and *Lissemys* spp.

Character 81: Dentary 1

**FIGURE 15:**
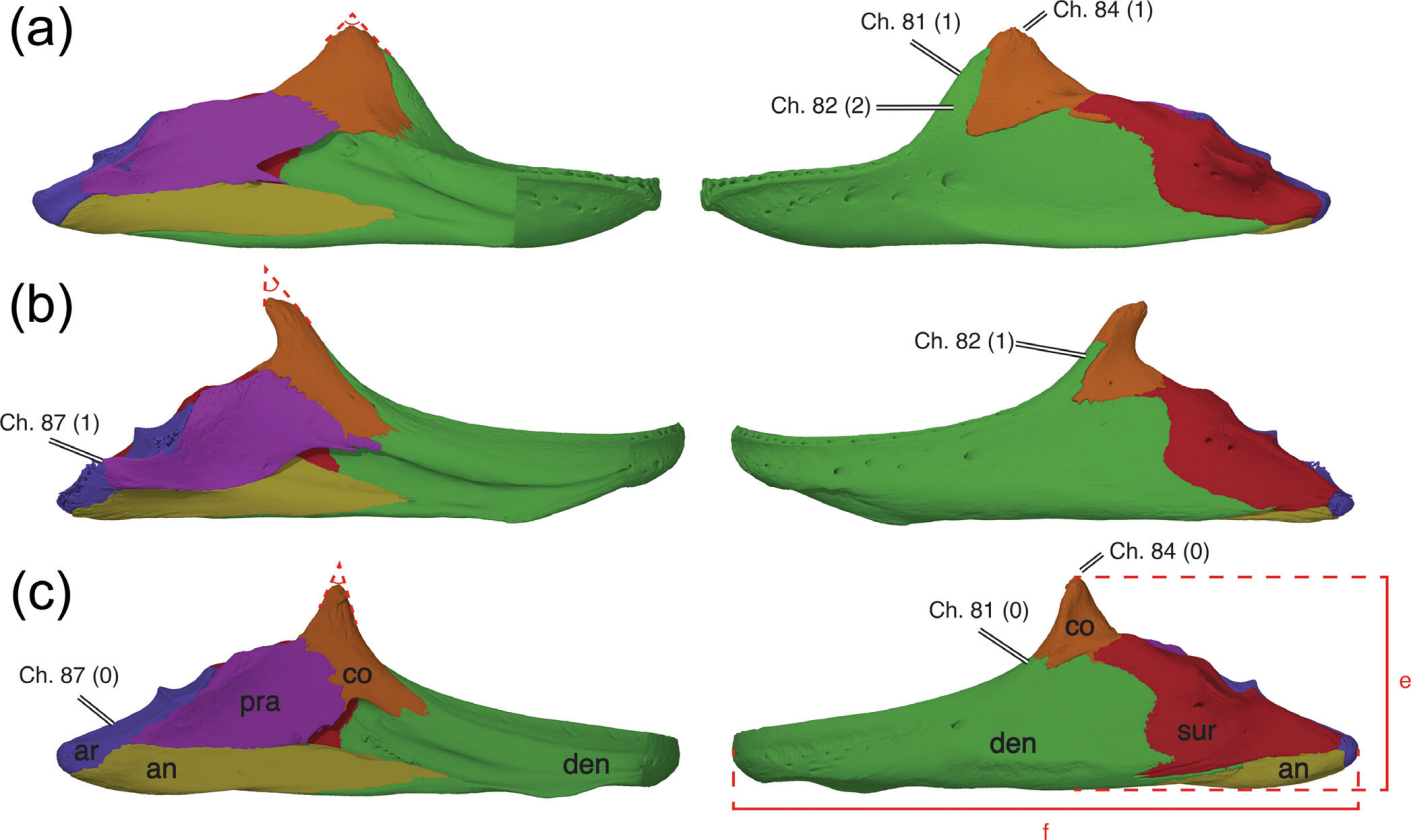
3D models of mandibles of select trionychid species in lateral and medial views illustrating character states and measurements used herein. (a) *Amyda cartilaginea* FMNH 224117, (b) *Pelochelys cantorii* PCHP 4974, (c) *Cycloderma aubryi* NMB 18041. Images are not to scale. *an* angular; *ar* articular; *co* coronoid; *den* dentary; *pra* prearticular; *sur* surangular. *e* height of the mandible at the coronoid process; *f* anteroposterior length of the mandible.

*Definition*—Dorsally ascending process of the dentary (Evers et al., 2022, 2): 0 = absent or indistinct (Figure 15c); 1 = present (Figure 15a).

*Comment*—Evers et al. (2022) recently suggested that a dorsally ascending process of the dentary (Figure 15) is an unambiguous synapomorphy of *Trionychidae*. Although we can confirm that this process is present in most trionychids (Figure 15a,b), it is indistinct in *Chitra* spp. and virtually absent in *Pelochelys* spp. (Figure 15c). No polymorphism is apparent in this character.

Character 82: Coronoid 1

*Definition*—Height of the coronoid process relative to the anteroposterior length of the mandible in lateral view (Figure 15c) (modified from Hirayama, 1985, 45): 0 = <30%; 1 = 30–40% (Figure 15b); 2 = >40% (Figure 15a).

*Comment*—Hirayama (1985) developed a discrete character capturing variation in the height of the coronoid process. Given that trionychids exhibit variation along a spectrum, we modified this character into a morphometric character that measures the height of the coronoid process relative to the length of the mandible from the anterior tip to the retroarticular process in lateral view (Figure 15c). Large amydines are found to have the proportionally highest coronoids (Figure 13a). *Apalone* spp., *Apalone mutica* especially, *Chitra* spp., and *Cyclanornbis senegalensis* have the lowest (Figure 13c). This character is ordered.

Character 83: Fossa Meckeli 1

*Definition*—Length of the coronoid in the medial wall of the fossa Meckelii (Figure 14e) (new character): 0 = absent or less than half of fossa Meckelii (Figure 13b); 1 = more than half of fossa Meckelii (Figures 13c and 14e).

*Comment*—This character documents the posterior extent of the coronoid within the medial wall of the fossa Meckelii. This character is best observed in 3D models, so our sampling is not optimal (Figure 14e). The condition in which the coronoid extends more than half of the length of the fossa Meckelii is found in *Apalone mutica*, *Cyclanorbis* spp., *Lissemys punctata*, and *Rafetus* spp. (Figure 14d,e) and is found polymorphically in *Amyda cartilaginea*, *Apalone spinifera* (Figure 13c), *Chitra indica. Dogania subplana*, *Nilssonia formosa*, *Nilssonia gangetica*, and *Lissemys ceylonensis*. It is consistently found absent in the remainder of the sample.

Character 84: Coronoid 3

*Definition*—Angle of the coronoid process in lateral view (new character): 0 = coronoid process pointed, with an angle less than 90° (Figure 15c); 1 = coronoid process blunt, with an angle greater than 90° (Figure 15a).

*Comment*—Usually, trionychids have a high, pointed coronoid process. In a few species of trionychids, this process is significantly blunter. To access this character, we measure the angle between the anterior and posterior edges of the coronoid process (Figure 15). A blunt coronoid process is found in *Apalone mutica*, *Cyclanorbis* spp., *Lissemys* spp., and *Rafetus euphraticus*. It is additionally polymorphically found in *Amyda cartilaginea* (Figure 15a), *Apalone ferox*, *Apalone mutica*, *Chitra*, *chitra*, and *Nilssonia nigricans*.

Character 85: Ectocondylar flange

*Definition*—Development of the ectocondylar flange of the surangular (modified from Meylan, 1987, 94): 0 = weakly developed, clearly smaller than the articular surface of the articular (Figure 13b); 1 = well developed, about the same size or larger than the articular surface (Figure 13c).

*Comment*—The ectocondylar flange of the surangular (Evers et al., 2022) forms the lateral aspect of the articular surface of the mandible (Figure 13). Meylan (1987) developed a character pertaining to the ectocondylar flange consisting of three character states, but as all trionychids possess a flange, we omitted the absence state. In most trionychids, the ectocondylar flange is a well‐developed surface the same size as the articular surface formed by the articular, or larger. The flange is clearly smaller than the articular surface of the articular only in *Chitra* spp. and some specimens of *Cycloderma aubryi* (Figure 13b).

Character 86: Retroarticular process 1

*Definition*—Length of the retroarticular process (modified from Meylan, 1987, 99): 0 = about the same size as the articular surface (Figure 13c); 1 = clearly longer than the articular surface (Figure 13e).

*Comment*—The retroarticular process of trionychids is formed by the articular, surangular and prearticular and is separated from the area articularis mandibularis by a raised ridge. Meylan (1987) developed a character that unites all trionychids relative to other turtles into a single character state characterized by the presence of a large retroarticular process, but we find the size of the retroarticular process to vary systematically among trionychid taxa (Figures 13, 14, 15). In most trionychids, the retroarticular process is about the same size as the articular surface, but a particularly long retroarticular process is consistently found in chitrines, *Apalone mutica*, *Cyclanorbis elegans*, and *Cycloderma* spp. (Figures 13b,d,e and 14c).

Character 87: Retroarticular process 2

*Definition*—Dorsal process of the prearticular in the retroarticular process (new character): 0 = absent (Figure 15c); 1 = present (Figure 15b).

*Comment*—This character documents the presence of a sheet‐like process ascending from the prearticular and forming a ridge on the medial edge of the retroarticular process of trionychids (Figure 15b). Among extant trionychids, this structure is unique to *Cycloderma* spp.

Character 88: Prearticular 1

*Definition*—Prearticular, contribution to area articularis surface (Evers et al., 2022, 51): 0 = absent (Figure 13c); 1 = present (Figure 13f).

*Comment*—The prearticular contributes to the area articularis in several species of trionychids. This condition is found present or polymorphic in all cyclanorbines, strictly present in *Chitra* spp., *Nilssonia formosa*, and *Rafetus swinhoei*, and polymorphic in *Amyda cartilaginea*, *Dogania subplana*, *Nilssonia gangetica*, *Nilssonia hurum*, *Nilssonia leithii*, *Pelochelys* spp., *Pelodiscus* sp., and *Trionyx triunguis*.

Character 89: Articular 1

*Definition*—Deep fossa just posterior to the area articularis (new character): 0 = absent (Figure 13c); 1 = present (Figure 13b).

*Comment*—The area articularis and retroarticular process of trionychids are often separated by a ridge. In some species, a lateromedially directed fossa is present just posterior to this ridge. This fossa is notably present in *Cycloderma* spp., *Nilssonia nigricans*, and *Palea steindachneri* and is polymorphically present in *Apalone mutica*, *Apalone spinifera*, *Cyclanorbis elegans*, *Nilssonia gangetica*, *Pelodiscus* sp., *Pelochelys bibroni*, and *Trionyx triunguis*.

Character 90: Hyoid 1

**FIGURE 16:**
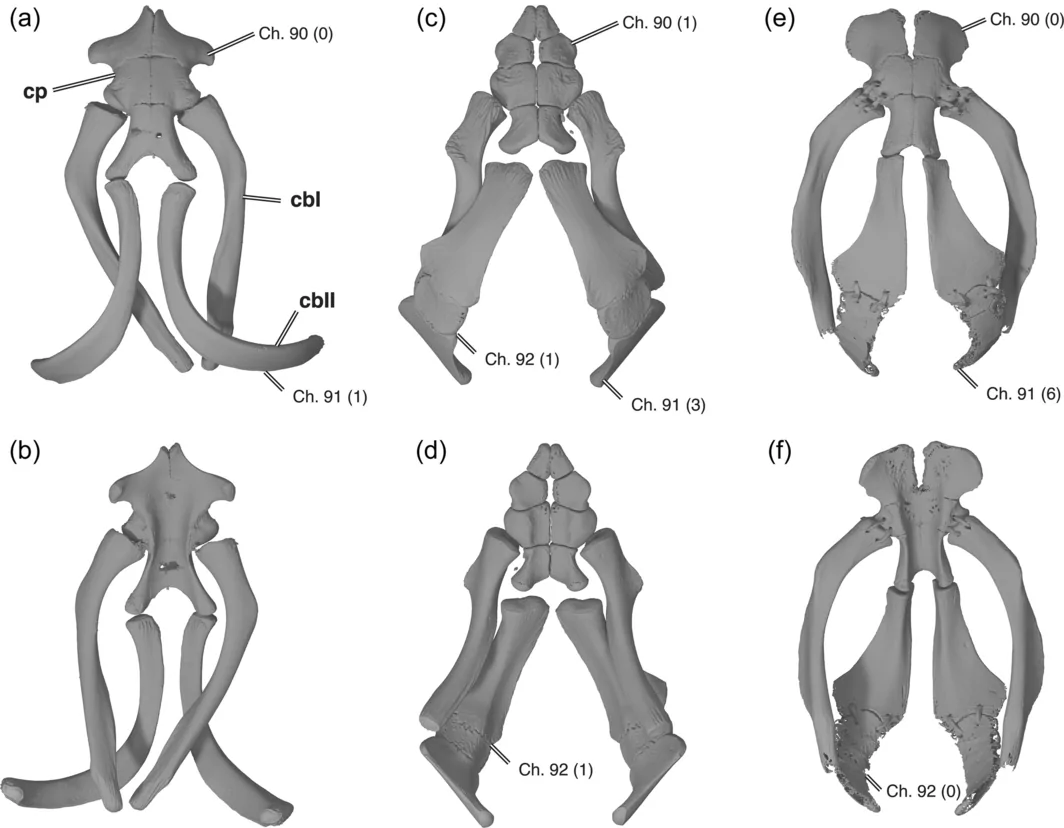
3D models of the hyoid apparatuses of select trionychid species illustrating character states. *Lissemys ceylonensis* NMB 2398 in (a) ventral and (b) dorsal view, *Pelochelys bibroni* UF H49435 in (c) dorsal and (d) ventral view, *Pelodiscus* sp. NMB 1438 in (e) ventral and (f) dorsal view. *cbI* cornu brachiale I, *cbII* cornu brachiale II, *cp* corpus hyoidis. Images are not to scale.

*Definition*—Number of ossifications in corpus hyoidis (modified from Meylan, 1987, 90): 0 = 6 (Figure 16a,e); 1 = 8 (Figure 16c).

*Comment*—We rephrase a character from Meylan (1987) by removing the state in which the corpus hyoidis is formed by a single ossification, as this condition never occurs in trionychids. Cyclanorbines, *Apalone mutica*, *Apalone spinifera*, all amydines except for *Dogania subplana*, and *Trionyx triunguis* have a corpus hyoidis formed by 6 ossifications. The remaining trionychid species possess a corpus formed by 8 ossifications.

Character 91: Hyoid 2

*Definition*—Number of ossifications in cornu branchiale II (Meylan, 1987, 91): X = X number of ossification (Figure 16).

*Comment*—We reuse a character from Meylan (1987) but modify the character states to have the character state matching the number of ossifications present in cornu brachiale II. All cyclanorbines have one single ossification in the cornu brachiale II (Figure 16a), amydines except for *Pelodiscus* sp., chitrines and *Apalone mutica* have two to seven ossifications (Figure 16b,c). *Pelodiscus* sp. and the remaining apalonines have 7 to 9 ossifications.

Character 92: Hyoid 3

*Definition*—Ossifications of cornu branchiale II broad and strongly sutured (Meylan, 1987, 92): 0 = absent (Figure 16f); 1 = present (Figure 16d).

*Comment*—This character was originally phrased by Meylan (1987). A broad and strongly sutured cornu brachiale II is exclusively found in *Chitra* spp. and *Pelochelys* spp.

### 3.2 Omitted characters

As part of this study, we scored our sample of specimens for numerous characters that were eventually omitted from the final matrix. We here briefly list some characters developed in previous studies and explain why we excluded them.

#### 3.2.1 Processus trochlearis

*Definition*—Contribution of the quadrate to the processus trochlearis oticum: 0 = <20%; 1 = 20–40%; 2 = >40% (modified from Meylan, 1987, 77).

*Comment*—Herein, we attempted to modify a character originally introduced by Meylan (1987) for turtles in general to be informative for trionychids in particular by adjusting the threshold to be situated within the range observed for this group of turtles. This character was discarded as it varies widely and apparently does not hold a phylogeny signal (i.e., shows no correlation with the currently accepted molecular consensus).

#### 3.2.2 Vomer

*Definition*—Vomer: 1 = present; 2 = absent (reworded from Meylan, 1987).

*Comment*—This character was introduced by Meylan (1987) as an autapomorphy of *Cycloderma frenatum*. However, while the vomer is highly reduced in this species, it is clearly present in all well‐preserved specimens available to us. We thus suspect that its common absence in museum specimens is a preservational artifact and remove this character from the list.

#### 3.2.3 Interorbital width

*Definition*—Interorbital width over the total width of the orbits: 0 = <20%; 1 = 20–30%; 2 = 30–40%; 3 = >40%.

*Comment*—We measured the interorbital width over the total width of the orbits to form a character pertaining to the interorbital space. This character shows strong intraspecific variation; however, resulting in many instances of polymorphism. In addition, no meaningful phylogenetic signal appears to be present.

#### 3.2.4 Epipterygoid

Meylan (1987) described much inter‐ and intraspecific variation in the trigeminal region of trionychids and developed six characters for the epipterygoid, of which three utilize scaled coding (i.e., character states are defined by the presence of polymorphism). We omitted all characters pertaining to this character complex for a number of reasons. First, the epipterygoid is often fused to the pterygoid in trionychids, reducing the number of specimens that can be scored in the first place. Second, the trigeminal area of most specimens is insufficiently documented in the photographs to which we had access, thus further limiting our sample to the few specimens for which we have access to CT scans. We were thus left with a poor sample for a very polymorphic character complex. Finally, even though Meylan's (1987) original sample was better than ours, none of his epipterygoid characters appear to yield a meaningful phylogenetic signal, perhaps because polymorphism is too rampant. For these reasons, we decided to disregard all characters relative to the epipterygoid and the trigeminal area.

#### 3.2.5 Hyoid characters

We retained three characters from Meylan (1987) related to the hyoid apparatus (see above), but disregarded one, as we could not replicate his observations. Additionally, we explored the study of Jorgewich‐Cohen et al. (2024), who developed a long character list for the hyoid apparatus of all turtles. We initially scored those characters reported by Jorgewich‐Cohen et al. (2024) to be variable for trionychids but ultimately discarded the full list for several reasons. First, several characters pertain to the presence or absence of cartilage or the degree of ossification of cartilaginous structures. As our matrix is aimed at resolving the phylogenetic relationships of fossil taxa, these characters were removed, as they cannot be scored for fossils. Second, we systematically could not reproduce the scoring of osteological characters presented by Jorgewich‐Cohen et al. (2024), likely because we used a different sample. This raises the possibility that all characters are affected by considerable polymorphism, thus questioning their utility. And third, it is our impression that many of the characters phrased by Jorgewich‐Cohen et al. (2024) are controlled by ontogeny, but our sample is far too incomplete to allow evaluating this claim. We therefore decided not to include any further hyoid characters in our matrix.

### 3.3 Analysis of character performance

We conducted several analyses that explore the effect of coding strategies upon phylogenetic reconstruction using four variations to our matrix. Our approach mirrors that of Joyce et al. (2025) for shell characters by comparing the results of an analysis to the emerging molecular consensus as a measure of phylogenetic accuracy. As a metric we utilize average quartet distances of all MPTs resulting from an analysis (Smith, 2019). The *MaxPoly* matrix includes the full range of polymorphism observed for a species in contrast to the *RedPoly* matrix, which omits polymorphism with a frequency below 20%. In the *Size60* matrix, polymorphism is further reduced relative to the *RedPoly* matrix by omitting observations from specimens with less than 60% of the maximum size of its species, while the *Size70* matrix omits polymorphism observed in specimens less than 70% of the maximum size of its species. These last two matrices aim to explore the impact of polymorphism resulting from ontogeny.

**FIGURE 17:**
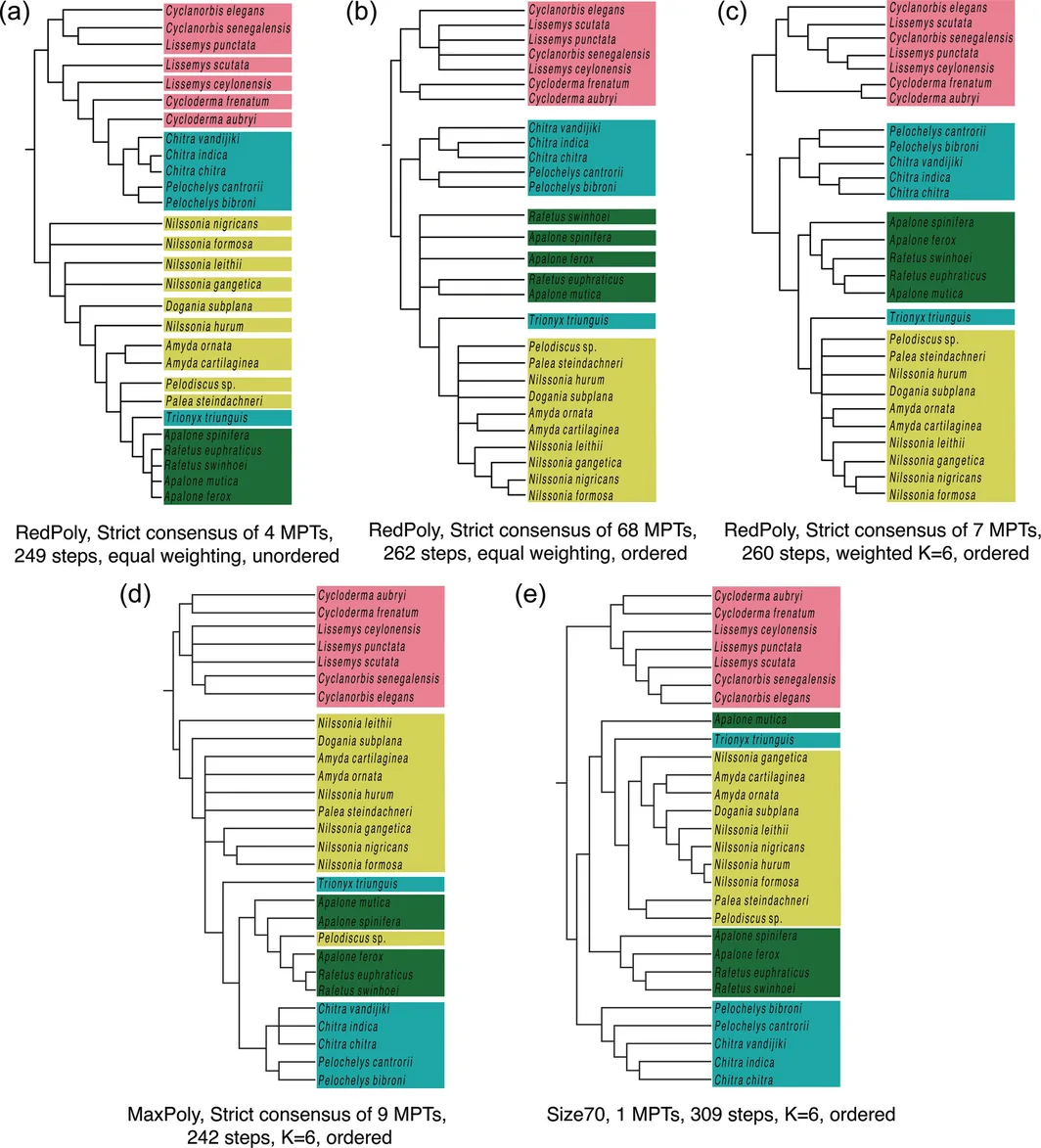
Trees resulting from the parsimony analyses. (a–c) strict consensus trees resulting from analyses of the *RedPoly* matrix; (d), strict consensus tree obtained from the *MaxPoly* matrix; and (e) single MPT resulting from analyses of the *Size70* matrix. The most important settings and results are listed below each tree.

Following Joyce et al. (2025) we ran an initial set of analyses using the *RedPoly* Matrix that vary in the ordering of characters and the choice of weighting scheme. These analyses did not include an outgroup. Instead, all trees were manually rooted to create a monophyletic *Cyclanorbinae* and *Trionychinae*. The first analysis using unordered characters and equal weights yielded 4 MPTs with a length of 249 steps. The average *Strict Joint Assertion* (*sja*) value of all trees is 62.0% (Table 1). The strict consensus (Figure 17a) only recovers *Apalonini* as monophyletic. *Cyclanorbinae* and *Amydini* are recovered as paraphyletic. *Chitrini* is polyphyletic as *Trionyx triunguis* is not recovered as sister to the remaining chitrines.

**TABLE 1:** Main metrics resulting from comparison analyses using the Quartet package (Smith, 2019) in R Studio.

Matrix	Outgroup	Settings	MPTs	Steps	Q	x̄s	x̄d	x̄sja
*RedPoly*	Manually rooted	EW, unordered	4	249	20,475	12,712	7762	62%
*RedPoly*	Manually rooted	EW, ordered	68	262	20,475	17,543	2931	85.6%
*RedPoly*	Manually rooted	K3, ordered	22	263	20,475	17,253	3221	84.2%
*RedPoly*	Manually rooted	K6, ordered	7	260	20,475	17,930	2545	87.5%
*MaxPoly*	Manually rooted	EW, unordered	601	218	20,475	17,419	3055	85.0%
*MaxPoly*	Manually rooted	EW, ordered	372	241	20,475	17,379	3095	84.8%
*MaxPoly*	Manually rooted	K6, ordered	9	242	20,475	17,930	2545	75.9%
*Size60*	Manually rooted	K6, ordered	1	294	20,475	16,962	3513	82.8%
*Size70*	Manually rooted	K6, ordered	1	309	20,475	17,485	2990	85.3%
*RedPoly*	*Carettochelys insculpta*	K6, ordered	1	332	20,475	17,050	3425	83.2%
*RedPoly*	*Perochelys lamadongensis*	K6, ordered	1	311	20,475	17,119	3356	83.6%
*RedPoly*	*Petrochelys kyrgyzensis*	K6, ordered	4	312	20,475	17,214	3260	84.0%

*Note*: Detailed results, the list of MPTs and scripts for the quartet analyses are reported in Data S7.

Abbreviations: Q, the total number of possible quartets for the trees; x̄s, the average number of quartets that are resolved identically in the MPTs and reference tree; x̄d, the average number of quartets that are resolved differently in the MPTs and reference tree; x̄sja, the average proportion of quartets resolved identically to the reference tree in percentage.

For the second analysis, all settings were left the same, but all characters that form morphoclines were set to be ordered (i.e., characters 4, 6–11, 13, 17, 21, 28, 31, 34–37, 44–46, 49–52, 55, 56, 63, 69, 72, 74, 75, 77, 79, 82, 91, using a regular counter starting at 1). This analysis resulted in 68 MPTs with 262 steps. The average *sja* value rises significantly relative to the unordered analysis from 62.0% to 85.6% (Table 1). The strict consensus (Figure 17b) resolves *Cyclanorbinae* and *Amydini* as monophyletic, *Apalonini* as paraphyletic, and *Chitrini* as polyphyletic. *Trionyx triunguis* again is not resolved as sister to the remaining chitrines. The significant increase in average *sja* value strongly suggests that the *RedPoly* matrix benefits from the ordering of morphoclinic characters. This parallels the result from Joyce et al. (2025) for trionychid shell characters.

For the third analysis, we retained use of ordered characters, but implemented a weighting factor of K = 3 and K = 6. The K = 3 analysis yielded 22 MPTs with 263 steps, while the K = 6 analysis yielded 7 MPTs with 260 steps (Table 1). Relative to the ordered analysis with equal weights, the average *sja* value is reduced to 84.2% using K = 3, but increases slightly to 87.5% using K = 6. This suggests for the present matrix that moderate weighting with a factor of K = 6 is slightly beneficial, but that stronger weighting has slightly negative effects. This result, too, parallels the results of Joyce et al. (2025). The strict consensus using this setting resolves *Amydini*, *Apalonini*, and *Cyclanorbinae* as monophyletic, but *Trionyx triunguis* is still not found as sister to the remaining chitrines (Figure 17c). While use of moderate weighting with a factor of K = 6 is apparently advantageous, we cannot make a recommendation for future analyses that expand upon this dataset, as the weighting scheme should always be adjusted to matrix size (Ezcurra, 2024; Goloboff et al., 2018). Unfortunately, as the present matrix should best be run using a molecular backbone (see further below), establishing an optimal K‐value will be difficult in the future, as the method we chose herein (i.e., unconstrained analyses compared to the molecular consensus) will not be applicable anymore. Future researchers will thus have to resort to recommendations from the literature (e.g., Ezcurra, 2024; Goloboff et al., 2018).

We replicated three of the above analyses (i.e., unordered, ordered, and ordered + K = 6) using the *MaxPoly* matrix to explore the impact of reducing polymorphism. The analyses retrieve 601, 372, and 9 MPTS, respectively, significantly more than using the *RedPoly* matrix (see Table 1). The number of steps, however, is reduced to 218, 241, and 242, respectively, suggesting that fewer character changes are needed to explain the trees. The average *sja* values are somewhat difficult to interpret at first glance. The analysis using unordered characters yielded an average *sja* value of 85%, which is significantly better than that obtained by the *RedPoly* matrix using the same settings (62%). The ordered analysis yielded a similar result at 84.8%, which is slightly poorer than the result using the *RedPoly* matrix (85.6%). The analysis using a weighting factor of K = 6, finally, yielded an average *sja* of 75.9%, which is not only poorer than the *RedPoly* matrix using the same setting, but also the *MaxPoly* matrix using other settings (see Table 1 for summary). The *MaxPoly* matrix thus appears to yield the best results if the analysis is run unordered and with equal weights, which opposes the conclusion we reached above for the *RedPoly* matrix. Using the mapping function of TNT, we are not able to explain these differences by changes to the number of informative characters, as there seems to be none. This contrasts with the results of Joyce et al. (2025) for the shell of trionychids, where the reduction of polymorphism increases the number of informative characters. We are thus left to wonder if our proposed reduction of polymorphism in combination with the ordering of characters and use of moderate weights is futile. We resolve this issue by noting that the *RedPoly* matrix apparently does not yield a better phylogenetic signal than the *MaxPoly* matrix, but does yield fewer trees, which, in return, yield a better resolved strict consensus tree (compare Figure 17c with Figure 17d). In addition, the reduced number of character transitions demanded by the *MaxPoly* matrix relative to the *RedPoly* matrix suggests that character step information is lost that is consistent with the tree (hence no loss in accuracy) and that might be useful in placing fossils in the tree. We thus conclude that the reduction of polymorphism by omitting frequencies below 20% is beneficial to the matrix, much as shown by Joyce et al. (2025) for trionychid shell data.

In contrast to Joyce et al. (2025), we were not able to further reduce polymorphism a priori by first establishing which characters are ontogenetically controlled and then omitting juvenile observations from affected characteristics, because most cranial characters influenced by ontogeny show diverging trends for each species (i.e., positive, neutral, or negative correlations with relative skull size) and because our sample is insufficient to address ontogenetic trends with confidence at the species level. As an alternative, we built two matrices that further reduce polymorphism relative to the *RedPoly* matrix by omitting observations obtained from individuals below a certain size threshold. Using the same settings, the *Size60* and *Size70* matrices both yield a single MPT with 294 and 309 steps, respectively, significantly more steps than the seven MPTs with 260 steps demanded by the *RedPoly* matrix using the same settings (i.e., ordered characters and K = 6). The average *sja* values are 82.8% and 85.3%, respectively, which are slightly lower, but comparable to the 87.5% obtained from the *RedPoly* matrix. The reduction of polymorphism resulting from the exclusion of character information obtained from smaller individuals yields fewer trees with slightly less accuracy (compare Figure 17c with Figure 17e) but far more character transitions. Although an increase in tree length may be considered undesirable in a parsimony context, we conclude that more steps imply more known character transformations across the trees, instead of leaving character states unresolved due to localized polymorphism. The available data thus yield more character evidence for the placement of fossils, much as the initial reduction in polymorphism by omitting frequencies below 20% did (see above). We thus recommend the use of the *Size70* matrix, as it yields good accuracy in combinations with optimal use of the available character information. However, as the only available tree obtained from this matrix still differs from the emerging molecular consensus, we strongly suggest running this matrix in a total evidence framework or with a molecular backbone.

**FIGURE 18:**
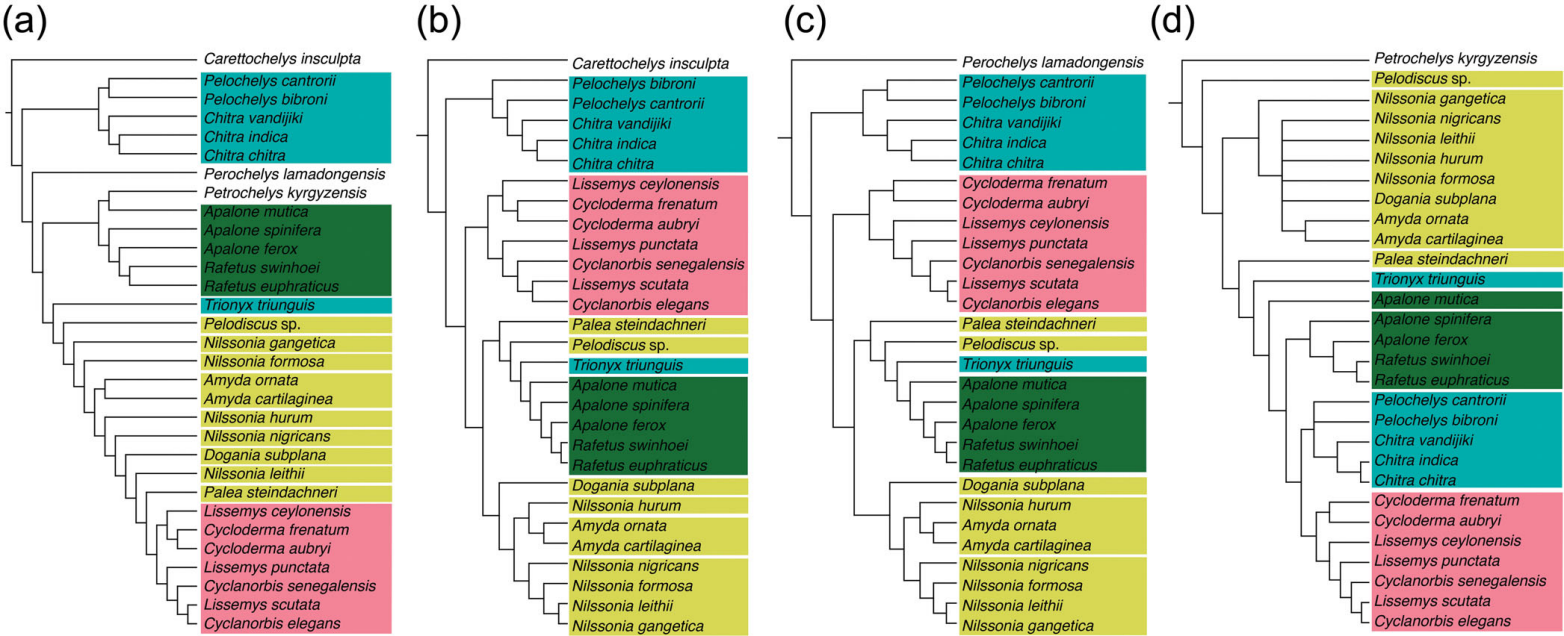
Trees resulting from the analyses including outgroup taxa, using the same settings as the analyses using the *Size70* matrix, and including additional taxa used as outgroups. (a) strict consensus of 1 tree using *Carettochelys inscuplta* as outgroup and including *Perochelys lamadongensis* and *Petrochelys kyrgyzensis*, (b) strict consensus of 1 tree using *Carettochelys inscuplta* as outgroup, (c) tree resulting from the analysis using *Perochelys lamadongensis* as the outgroup, (d) tree resulting from the analysis using *Petrochelys kyrgyzensis* as the outgroup.

A long‐standing issue in trionychid systematics is the negative impact of outgroups, which not only pull particular taxa to the base of the tree, but also change tree structure overall (see summary in Joyce, 2025). We explore the impact of outgroups on the present matrix by adding three taxa to our *Size70* matrix, in particular *Carettochelys insculpta*, which is universally agreed to be the extant sister of *Trionychidae*, and *Perochelys lamadongensis* (scored from Li, Joyce, & Liu, 2015) and *Petrochelys kyrgyzensis* (scored from Vitek et al., 2018), the two earliest known (i.e., Early Cretaceous) fossil pan‐trionychids with cranial remains. Using the above discovered optimal settings for the matrix (i.e., ordered character, K = 6), analyses using each of these three taxa as an outgroup yielded similar results to the manually rooted *Size70* matrix (see Table 1), also in regard to accuracy, which is 83.2%, 83.6%, and 84.0%, as opposed to 85.3%. The inclusion of all three potential outgroups thus has no negative impact on the overall accuracy of the analysis. However, none of the outgroups is able to retrieve a monophyletic *Cyclanorbinae* relative to a monophyletic *Trionychinae* (see Figure 18). Indeed, while *Carettochelys insculpta* and *Perochelys lamadongensis* pull *Chitra* + *Pelochelys* to the base of the tree, *Petrochelys kyrgyzensis* pulls amydines, in particular *Pelodiscus* sp., to the base of the tree. These results differ from Joyce et al. (2025) by concluding that the skulls of potential outgroups have no negative impact on overall topology, but agree in that outgroups are not able to correctly polarize the tree. However, as polarity is essential for correctly placing fossils, especially basal taxa, we are left to conclude, like Joyce et al. (2025), that polarity is best elucidated by either including additional fossils from the base of the tree or in a Bayesian framework that takes stratigraphy into account. As we are unaware of additional fossil material, we recommend the use of a Bayesian framework for the moment.

In conclusion, we find evidence that the present matrix yields the most accurate phylogenetic results if polymorphism is reduced by omitting frequencies below 20% and observations from juvenile to subadult individuals below a maximum size of 70% in addition to the ordering of multistate characters that form morphoclines and light weighting. Although the available outgroups have no negative impact upon overall topology, none are able to correctly polarize the base of crown *Trionychidae*. We thus suggest running expanded versions of this matrix that include fossils within a Bayesian framework that takes stratigraphy into consideration, ideally using a total evidence approach or a molecular backbone.

### 3.4 Sources of polymorphism among trionychid turtles

In the following section we evaluate how polymorphism in our matrix is controlled by sampling and if our present sample is sufficient to correctly document polymorphism. To do so, we calculate the level of polymorphism observed for each species in three select matrices (*MaxPoly*, *RedPoly*, and *Size70*) by dividing for each species the number of polymorphic characters by the number of coded characters. Use of the number of coded characters, not the total number of characters, is necessary as 8 out of 28 terminal taxa in our *MaxPoly* and *RedPoly* matrices and 12 out of 28 taxa in our *Size70* matrix are not coded for all characters due to low sampling (e.g., *Lissemys scutata*, *Chitra vandijki*) and/or a lack of the CT scan data needed to score characters related to the internal anatomy of the skull (e.g., *Rafetus swinhoei*, *Nilssonia hurum*, *Amyda ornata*). The number of sampled specimens is cut from 303 to 162 in the *Size70* matrix relative to the other two matrices, as specimens are disregarded smaller than 70% of the maximum size of each species. As a result, the average sampling size is 10.8 specimens per taxon for the *MaxPoly* and *RedPoly* matrices and 5.7 for the *Size70* matrix. We observe, on average, 32.5% polymorphic characters per species in the *MaxPoly* matrix, 14.9% in the *RedPoly* matrix, and 16.5% in the *Size70* matrix (Data S8). The latter number is surprising at first glance, as one would expect a reduction in specimens to yield less polymorphism, but some polymorphism reemerges in the *Size70* matrix relative to the *RedPoly* matrix, as many species do not have enough specimens anymore to disregard polymorphism under 20% frequency.

The least polymorphic taxa are ones for which we only have one available specimen, in particular *Chitra vandijki* and *Lissemys scutata*. The low level of polymorphism present in these species is a result of morphometric measurements being close to threshold values. The most polymorphic taxa in the *MaxPoly* and *Size70* matrices are *Pelodiscus* sp. (55.4%, *N* = 24 versus 33.6%, *N* = 11) followed by *Dogania subplana* (52.2%, *N* = 12 versus 30.4%, *N* = 6) and *Apalone spinifera* (50%, *N* = 14 versus 28.2%, *N* = 6). Meanwhile, in the *RedPoly* matrix, the most polymorphic species is *Dogania subplana* (28.5%, *N* = 12) followed by *Pelodiscus* sp. (24.8%, *N* = 24) and *Apalone spinifera* (24.8%, *N* = 14) (see Data S8 for details).

**FIGURE 19:**
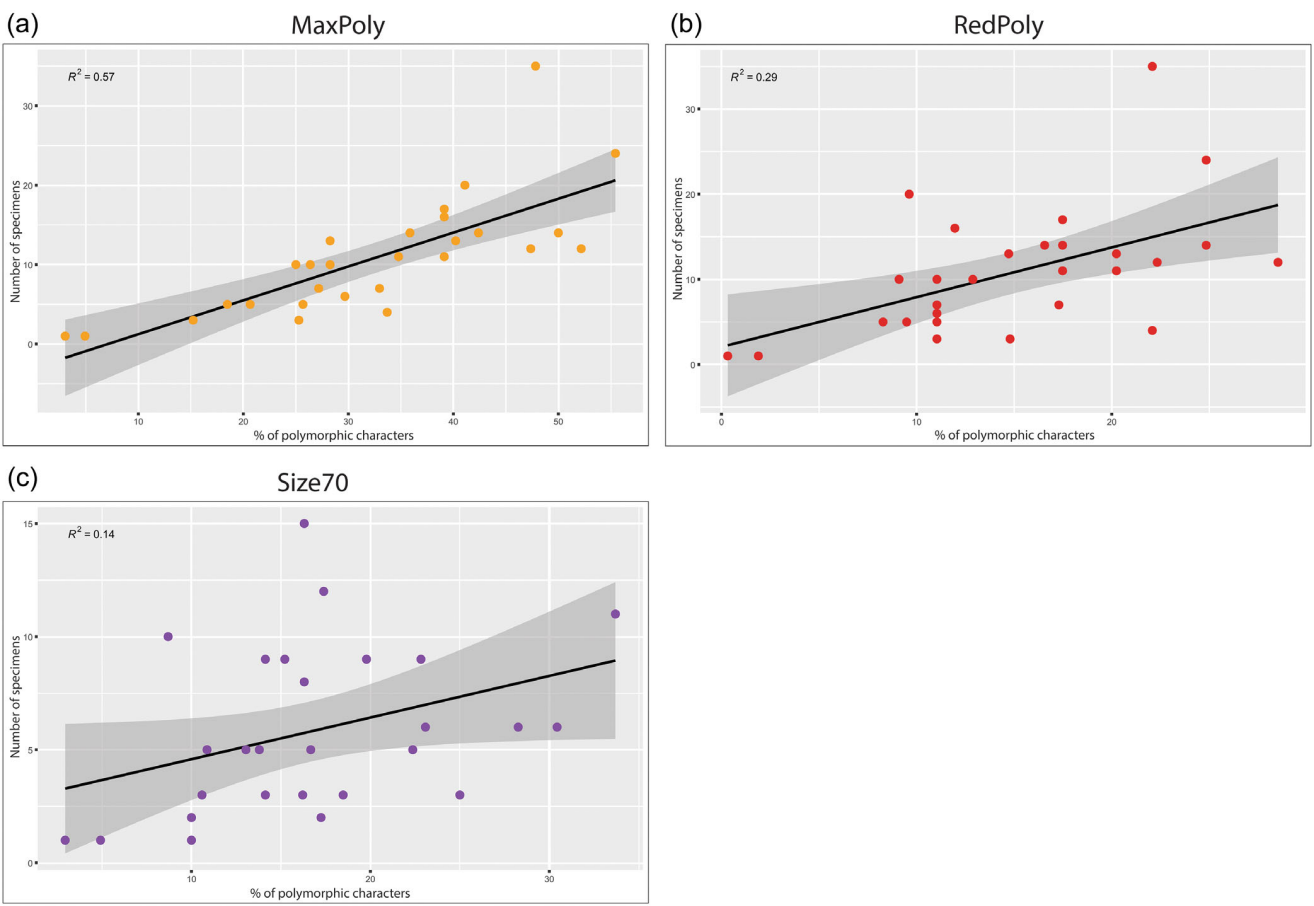
Graphic of the level of polymorphism in a species in percentage in relation to the absolute number of specimens per species in the (a) *MaxPoly*, (b) *RedPoly*, and (c) *Size70* matrices, matrix, with the linear trendline.

We calculated linear and logarithmic correlation coefficients for the relationship between sample size and observed levels of polymorphism to explore the effect of sampling upon polymorphism (Figure 19). The *R* ^2^ values for a linear trend for the *MaxPoly*, *RedPoly*, and *Size70* matrices are 0.57, 0.29, and 0.14, respectively, showing a decreased relationship between sample size and observed polymorphism when polymorphism is reduced by disregarding frequencies below 20% and juvenile morphotypes. These numbers suggest that levels of polymorphism observed in the *MaxPoly* matrix are strongly correlated with sample size. Additional sampling is thus expected to further increase polymorphism for this matrix, even for species with good sampling. The situation is strongly different for the *Size70* matrix, where levels of polymorphism only correlate poorly with sample size, thus suggesting that the reduction of polymorphism related to low‐frequency variations and ontogeny lowers the need for additional sampling. Our present sample thus appears overall sufficient. The heterogeneity in the *Size70* data, however, demands explanation, as some species are apparent with good sampling combined with low polymorphism, while others show low sampling and high polymorphism. We will explore possible causes of this variation below.

In the *Size70* matrix, *Apalone ferox* ranks 1st in regards to sample size (*N* = 15), but 13th in regards to polymorphism (16.3% of characters are polymorphic). This suggests that the available population is disproportionally homogeneous relative to other species. We suspect that this is related to the fact that *Apalone ferox* is a species with a relatively restricted spatial distribution (TTWG, 2025) across a flat, subtropical landscape that likely eases gene flow. We are unaware of studies that investigate genetic diversity for this species, but suspect it to be low. This situation contrasts with *Apalone spinifera*, which ranks 10th in regards to sample size (*N* = 6), but 3rd in regards to polymorphism. This species thus shows disproportionally high levels of polymorphism. The five specimens with locality data originate from western Texas (TMM M7158), central Texas (TMM M3817), Louisiana (YPM R10893), and southern Ontario (ROM R9385, ROM R9386) and therefore represent up to four currently recognized subspecies (TTWG, 2025). This is likely related to the fact that *Apalone spinifera* is distributed across much of the temperate humid parts of North America and that both morphological and molecular studies suggest strong regional partitions associated with distinct river basins that likely diverged from one another during the late Neogene (McGaugh et al., 2008; TTWG, 2025). So, while we are confident that we correctly sampled polymorphism for *Apalone ferox*, it may be desirable in the future to better capture variability for *Apalone spinifera* by sampling subspecies separately.

A special case is *Pelodiscus* sp., which ranks 3rd in regards to sampling (*N* = 11) and 1st in regards to polymorphism (33% of characters). As late as the late 1980s, the genus *Pelodiscus* was believed to consist of only a single species, *Pelodiscus sinensis*, with a wide range across much of eastern Asia (Ernst & Barbour, 1989), but a series of more recent molecular analyses have recognized several distinct molecular lineages with divergences reaching back to the Miocene that are now typically recognized as distinct species of *Pelodiscus* (Farkas et al., 2019; Fritz et al., 2010; Gong et al., 2022; Tang, 1997; Zhou et al., 1991). The TTWG (2025) currently recognizes six distinct species of *Pelodiscus* while noting that some of these species might be recognized as subspecies in the future as some studies find evidence of mixing (Xiong et al., 2019). As the osteology of all newly recognized lineages has not yet been documented, all dry specimens of *Pelodiscus* in our sample are cataloged in the collection under the name *Pelodiscus sinensis*, but it is likely that our sample of 22 specimens includes more than one species. The vast majority of *Pelodiscus* in our sample are specimens obtained from markets, plausibly in China and Japan, and thus likely represent farmed hybrids with different levels of admixture from two or more species (Gong et al., 2018). The high levels of polymorphism we observe for this taxon may thus be partially related to broad geographic sourcing of the animals in our sample in combination with early effects of domestication. This issue can only be addressed by a full‐scale research program aimed at broadly sampling wild‐caught specimens that are confirmed to represent a particular lineage using molecular data and then μCT scanned to obtain the osteological data. This would clarify the coding of various *Pelodiscus* species in morphological matrices and enable elucidating the phylogenetic relationships of fossil *Pelodiscus* (Chow & Yeh, 1958; Yeh, 1963).

## 4 CRANIAL, MANDIBULAR, AND HYOID CHARACTERISTICS OF EXTANT TRIONYCHIDS

In the following section, we succinctly describe the main characteristics of the different groups and species of extant trionychids. We do not provide an exhaustive list of autapomorphies for a given taxon but focus on general, external features that help in the identification of trionychids. While many trionychid species bear many skull and hyoid features that can help identify them to the species level, these observations should ideally be completed with features of the shell (see Joyce, 2025) in combination with locality data.

### 4.1 Cyclanorbinae

The skulls of cyclanorbines are distinct from those of trionychines in several morphological traits. Cyclanorbines most notably have short faces (i.e., the orbits are set close to the external nares) and long postorbital bars. The maxillae are strictly excluded from the foramina jugulare posterius, which are often subdivided into two or more small foramina. The foramina alveolare superius and the vertical flange of the pterygoid are absent. Cyclanorbines have short bony palates with small, anteriorly positioned intermaxillary foramina. The length of the intermaxillary contact is accordingly short. The internal choanae of cyclanorbines are elongated and their anterior limits are anteriorly positioned. The occipital area of cyclanorbines is distinct from other trionychid species in that the foramen jugulare posterius is consistently separated from the fenestra postotica. This separation is always undertaken by the pterygoid. The hyoid apparatus of cyclanorbines is composed of a corpus hyoidis of six ossifications and a cornu brachiale II that is formed by a single ossification and that is longer than cornu brachiale I.

### 4.2 Cyclanorbis

*Cyclanorbis* skulls have several distinctive traits, in particular the presence of a ridge on the dorsal surface of the opisthotic and the covering of the triple junction of the otic capsule by the parietal. The processus paroccipitalis of *Cyclanorbis* is wide and begins just lateral to the opisthotic‐exoccipital contact that separates the foramen jugulare posterius from the fenestra postotica, which also creates a narrow space between the processus paroccipital and the supraoccipital crest. The processus trochlearis oticum in most specimens exhibits a distinct, medial bump that is formed by the parietal and that creates an angle of almost 90° between the medial and mid portion of the processus trochlearis oticum. The coronoid process is low and blunt.

*Cyclanorbis elegans* is the only species of trionychids with a medial emargination of the external nares. The frontals usually have only a short or lacking medial contact as the posteromedial processes of the prefrontal on the skull surface. The frontal only contributes little to the orbit compared to other trionychids. The palatal area consists of a broad triturating surface, a tight palatal groove, and a greatly reduced intermaxillary foramen to which the premaxilla does not contribute. The triturating surfaces are particularly elongate, posteriorly reaching the level of the otic capsule, and delineated by a lingual ridge that strongly thickens anteriorly. In about half of the specimens, the pterygoids medially contact one another and thus prevent contact between the palatine and parabasisphenoid in ventral view. The mandible is tall, but robust and the coronoid process is blunt. The ectocondylar flange is wide and reaches far anteriorly. The fossa Meckelii is short.

The skull of *Cyclanorbis senegalensis* has an ovoid general shape. The face is short and wide. The orbits are dorsally positioned and the interorbital bar is narrow. The dorsal surface of the skull is mainly formed by the parietals. The vomer does not contact the prefrontal. The external nares are particularly wide in frontal view. The nasal bar is about half the width of the orbits. The intermaxillary contact is short to absent. The vomer always contributes to the posterior limit of foramen intermaxillare. The triturating surface is narrow but and the internal choanae are wide. The palatines posteriorly reach behind the level of the anterior margin of the processus trochlearis oticum. The parietals are generally inflated to form a domed structure. The pterygoid forms a notch anterior to the articular condyle of the quadrate. The anteromedial flanges of the mandible are well‐developed. The coronoid process is short and blunt. The contact of the prearticular and surangular over the fossa Meckelii is long and the fossa Meckelii is therefore positioned anteriorly.

### 4.3 Cycloderma

*Cycloderma* skulls are flattened and elongated. The face is short, the postorbital bar is longer than the anteroposterior length of the orbit, and the labial ridge is relatively high. The premaxillae are reduced to absent and the free medial flange of the maxilla extends into the internal choanae. The highly reduced vomer forms a thin rod of bone that is easily damaged.

*Cycloderma frenatum* can be distinguished from *Cycloderma aubryi* by having a more rounded snout with more anteriorly positioned orbits and a smaller interorbital length. *Cycloderma frenatum* has a more thickened triturating surface that widens posteriorly, while those of *Cycloderma aubryi* narrow posteriorly. In *Cycloderma frenatum*, the pterygoid participates in the triturating surface. The free medial flanges of the maxilla are more developed in *Cycloderma aubryi* than in *Cycloderma frenatum* and clearly constrict the anterior part of the internal choanae. In *Cycloderma aubryi*, the jugal is excluded from the orbit margin and there is no medial contact of the exoccipital. In lateral view, the profile of *Cycloderma aubryi* is particular in that the dorsal limit of the orbit is higher than the anterior half of the cavum cranii, giving the skull a constricted shape behind the orbits. In *Cycloderma aubryi*, foramen stapedialis posterius is separated from fenestra postotica more frequently than in *Cycloderma frenatum*.

The mandible of *Cycloderma* is elongated. The rami are narrow, the posteromedial flanges are minor to absent. The retroarticular process forms a deep notch and a medial process ascends from the retroarticular process. The mandible of *Cycloderma aubryi* is generally slimmer and narrower than that of *Cycloderma frenatum*. *Cycloderma aubryi* has a high and pointed coronoid process while this process is notably short in *Cycloderma frenatum*.

### 4.4 Lissemys

General features of *Lissemys* species include a long skull with a short snout, large orbits close to the external nares, and weak narial emargination that gives the snout a “square” outline. The frontals are elongated and contribute little to the orbit. The suborbital crest is well defined. The intermaxillary foramen and internal choanae are elongated. The triturating surfaces are wide. The palatal groove is tight and deeply set. The palatine reaches far posteriorly, contributes much to the roof of the choanae, and forms a deep vaulted narial passage. The coronoid process is short.

*Lissemys punctata* generally has longer orbits, shorter postorbital bars, and domed parietals compared to the other species of *Lissemys*. The parietal covers the triple junction of the supraoccipital, opisthotic, and prootic in *Lissemys punctata* and *Lissemys scutata*. In ventral view, the pterygoid overlaps the parabasisphenoid in some specimens of *Lissemys punctata*. *Lissemys punctata* has a particularly blunt coronoid process. In *Lissemys ceylonensis*, there is no notch in the pterygoid anterior to the articular condyle and the ridge of the M. pterygoideus is confluent with the condyle.

### 4.5 Chitrini

*Chitrini* is a heterogeneous group of trionychids mostly united by molecular data. The skull exhibits a low to absent suborbital crest, no participation of the jugal in the vertical flange of the pterygoid, an absence of a ridge within the cavum tympani, and retroarticular processes that are longer than the articular surface.

### 4.6 Chitra

The skull of *Chitra* can reach a length of more than 30 centimeters. The cranium is flat, has a notably short face, and the orbits are located far anteriorly. The postorbital is at least two times as long as the orbit. The processus trochlearis oticum is elongated, giving the temporal fossa an elongated, lenticular shape. The triturating surface exhibits accessory ridges. The choanae are almost completely covered by a posterior expansion of the maxilla. The palatines develop several foramina palatinum accessorium. Due to the elongation of the skull, the parabasisphenoid and pterygoids are elongated. The parabasisphenoid protrudes deeply between the palatines. The pterygoid forms a bony lamina that floors the foramen posterius canalis carotici interni and develops secondary basioccipital tubercules. The general shape of the mandible is elongated. The symphysis is notably short. No posteromedial flanges are developed. A deep groove runs along the triturating surface to accommodate the accessory ridges of the maxilla. A ridge runs anteroposteriorly over the elongate retroarticular process, approximately along the articular–surangular suture. The coronoid process is short. The corpus hyoidis is formed by eight ossifications. The cornu brachiale II is composed of three broad, strongly sutured segments. The distal end of the cornu brachiale II is rounded.

The different species of *Chitra* are difficult to differentiate from the skull anatomy alone. We are unaware of apomorphic features for *Chitra vandijki*, mostly because our sample only includes a single, damaged specimen. The observed difference between *Chitra indica* and *Chitra chitra* is subtle but consistent. In *Chitra indica*, the ridge originating from the temporal bar reaches above the ventral limit of the orbit while in *Chitra chitra* the ridge reaches just to the level of the ventral limit of the orbit.

### 4.7 Pelochelys

The skull of *Pelochelys* is flat, but broad. The snout is short and triangular. The orbits are large and widely spaced. The postorbital bar is longer than the orbit but never more than twice the orbit length. The processus trochlearis oticum is anteroposteriorly oriented rather than horizontal, but the temporal fossa is not elongated. The palate is notably wide and flat. A lingual ridge or palatal groove is absent. The intermaxillary foramen is relatively small. The canalis intrapalatinus is uniquely reduced to a foramen. A ridge formed by the pterygoid floors the foramen posterius canalis carotici interni. The mandibular groove accommodating the accessory ridge of the maxilla is absent in *Pelochelys*. The dentary lacks a coronoid process. The hyoid apparatus of *Pelochelys* is very similar to *Chitra*, but the distal end of the cornu brachiale II has a pointed process in *Pelochelys*.

We find two characters that distinguish *Pelochelys bibroni* from *Pelochelys cantorii*, but both characters show some polymorphism. The basisphenoid of *Pelochelys bibroni* typically reaches far anterior between the palatines where it polymorphically contacts the vomer. The parabasisphenoid of *Pelochelys cantorii*, by contrast, does not reach as far anteriorly, and thus consistently does not contact the vomer. In addition, the postorbital of *Pelochelys bibroni* forms an elongate posterior process that polymorphically contributes to the margin of the temporal emargination. This process is consistently shorter in *Pelochelys cantorii* and thus never contributes to the temporal emargination. Our sample does not include the skull of *Pelochelys signifera*, so we cannot comment on apomorphic characters for this species.

### 4.8 Trionyx triunguis

*Trionyx triunguis* has an elongated snout, deeply emarginated external nares, and notably elongated prefrontal bones. The intermaxillary foramen is larger than the internal choanae. The intermaxillary contact is long. The internal choanae are thus located far posteriorly. The contact between the exoccipital and pterygoid is autapomorphically absent for *Trionyx triunguis*, as previously noted by Meylan (1987). In addition, a deep notch is uniquely formed by a ridge extending from the descending process of the prefrontal. Finally, large postorbitals that often completely prevent contact between the jugal and parietal and extend ventrally to almost reach the palatine are autapomorphically developed for this taxon. The mandible is spatulate, the rami are slim, but lack posteromedial flanges, and the retroacticular processes are longer than the articular surface. The corpus hyoidis is formed by six ossifications and the cornu brachiale II is made of several small ossifications.

### 4.9 Amydini

Amydines share a number of cranial and mandibular features that distinguish them from other trionychids, but individual species are difficult to distinguish from one another using characters from the skull and hyoid. General features of amydines include a long snout, an extended intermaxillary contact, intermaxillary foramina that are smaller than the internal choanae, and a short postorbital bar. The vomer usually forms a flat surface posterior to the bony palate. In most species, foramina are present on the flat surface formed by the vomer. The pterygoid participates in the posterior portion of the triturating surface and the jugal generally participates in the vertical flange of the pterygoid. They have a process formed by the opisthotic that partially or fully separates the foramen jugulare posterius from the fenestra postotica. Amidines have the highest mandible among trionychids. The hyoid apparatus of amydines is similar to that of *Trionyx triunguis*, in that cornu brachiale II is composed of several small, round elements that do not exceed 7. In *Pelodiscus* sp. the most distal ossification is large and flat.

In *Amyda*, the posterior limit of the bony passage of the internal choanae is the most posteriorly located among trionychids. *Amyda* has a well‐defined suborbital crest. *Amyda* has, with *Dogania subplana*, the shortest mandibular symphysis length among amydines. *Amyda* exhibits a well‐defined symphyseal ridge within a depression. The differences between *Amyda cartilaginea* and *Amyda ornata* are subtle and inconsistent.

*Dogania subplana* has a slender, elongated snout and greatly reduced postorbital bar, the shortest among trionychids. In addition, the dorsal surface of the skull, which is mostly formed by the parietals, is greatly reduced. *Dogania subplana* has a tight palatal groove. There is no symphyseal ridge on the mandible. Its symphysis is the shortest among amydines.

*Nilssonia* species generally have robust skulls and moderately wide palatal grooves. A groove connects the foramen palatinus anterius to the foramen supramaxillare. *Nilssonia hurum* has the tightest palatal groove but the most widely spaced orbits among *Nilssonia* species. The symphyseal ridge is faint. *Nilssonia gangetica* stands out by having a broad skull, a relatively short and rounded snout, robust triturating surfaces, the broadest palatal groove among *Nilssonia* species, a high suborbital height, and the tallest mandible among trionychids. The symphyseal ridge is quite distinct from other amydines, by being developed on the posterior margin of the symphysis and by being associated with well‐defined posterolateral facets. *Nilssonia leithii* has the widest triturating surfaces among *Nilssonia* species. The symphyseal ridge is relatively faint. *Nilssonia nigricans* and *Nilssonia formosa* have well‐defined symphyseal ridges.

*Palea steindachneri* and *Pelodiscus* sp. are small species with a long and slender snout and face. The mandibles have elongated symphyses and are spatulate in shape. *Palea steindachneri* has notably strong narial emargination, and the palatines contribute minorly to the roof of the narial passage. The triturating surface widens greatly posteriorly. Within our sample, we observe much variation for *Pelodiscus* sp., but as we cannot assign particular specimens to species, we cannot assess if any of this variation is diagnostic for particular subclades or species.

### 4.10 Apalonini

*Apalonini* is easily recognized by the presence of a large intermaxillary foramen, the largest among extant trionychids, and large choanae, giving them a particularly short bony palate, and a wide palatal groove. The maxillae exhibit little to no midline contact, leaving the vomer exposed in dorsal view. As a result, the vomer forms the posterior limit of the intermaxillary foramen. The anteromedial flange of the mandible is absent or minor. The hyoid consists of a cornu brachiale II made up of a greater number of ossifications than in other trionychids.

### 4.11 Apalone

*Apalone* skull has an elongated skull with closely spaced orbits. *Apalone* has the strongest narial emargination among trionychids and the lowest participation of the palatine to the roof of the internal narial passage. The foramen jugulare anterius is often located within the secondary wall of the braincase. *Apalone ferox* is significantly larger than the other *Apalone* species, and some large specimens develop enlarged triturating surfaces. The internal choanae of *Apalone ferox* are the widest among trionychids. The internal choanae of *Apalone mutica* and *Apalone spinifera*, while large, are elongated in contrast to the rounder shape of the internal choanae in *Apalone ferox*. *Apalone spinifera* and *Apalone mutica* have notably small postorbital bars. The mandible of *Apalone mutica* has slender rami, lacks posteromedial flanges, and has a notably spatulate‐shaped symphysis.

### 4.12 Rafetus

*Rafetus* has a shorter snout than *Apalone* and a weaker narial emargination. *Rafetus*, especially *Rafetus swinhoei*, has a rounder snout than *Apalone*. In *Rafetus*, as well as *Apalone ferox* the foramen palatinus anterius is located within the suborbital crest. The vomer of *Rafetus*, contrary to *Apalone*, extends far posteriorly between the palatines, and in most specimens of *Rafetus euphraticus*, it reaches the parabasisphenoid and separates the palatines completely in ventral view. *Rafetus swinhoei* is larger than *Rafetus euphraticus*. *Rafetus euphraticus* has a particularly deep notch in the pterygoid anterior to the articular condyles. Both *Rafetus* species have ridges on the dorsal surface of the opisthotic. The mandible of *Rafetus* has a moderately long symphysis, moderate height and a blunt coronoid process.

## AUTHOR CONTRIBUTIONS

**Léa C. Girard:** Conceptualization; investigation; writing – original draft; formal analysis; data curation; writing – review and editing; visualization; methodology; software; validation. **Walter G. Joyce:** Conceptualization; investigation; writing – original draft; funding acquisition; writing – review and editing; methodology; supervision; validation; visualization.

## FUNDING INFORMATION

This project was funded by a grant from the Swiss National Science Foundation to WGJ (SNF 200021_207377).

## CONFLICT OF INTEREST STATEMENT

The authors declare no conflicts of interest.

## Supporting information


**Data S1.** List of specimens and associated morphometric measurements.

**Data S2.** Specimen matrix.
**Data S3**. *MaxPoly* matrix.
**Data S4**. *RedPoly* matrix.
**Data S5**. *Size60* matrix.
**Data S6**. *Size70* matrix.

**Data S7.** Complete list of MPTs and associated quartet analyses results derived from the parsimony phylogenetic analysis.

**Data S8.** Excel sheet reporting the level of polymorphism within the different matrices.

## Data Availability

The CT data and 3D models presented in this study are available in open access at the online repository MorphoSource (https://www.morphosource.org/projects/000739536) and at the NHMW Data Repository (https://doi.org/10.57756/gtse5e) for NHMW specimens. The photographs presented in this study are available in open access on the data depository Dryad (https://doi.org/10.5061/dryad.jq2bvq8m2).
